# A Randomised, Placebo-Controlled, First-In-Human Study of a Novel Clade C Therapeutic Peptide Vaccine Administered *Ex Vivo* to Autologous White Blood Cells in HIV Infected Individuals

**DOI:** 10.1371/journal.pone.0073765

**Published:** 2013-09-17

**Authors:** Akil Jackson, Henrik N. Kløverpris, Marta Boffito, Amanda Handley, Mark Atkins, Peter Hayes, Jill Gilmour, Lynn Riddel, Fabian Chen, Melanie Bailey-Tippets, Bruce Walker, Jim Ackland, Mark Sullivan, Philip Goulder

**Affiliations:** 1 St Stephen’s AIDS Trust, Chelsea and Westminster Hospital, London, United Kingdom; 2 Department of Paediatrics, University of Oxford, Oxford, United Kingdom; 3 Medicines Development, Melbourne, Victoria, Australia; 4 Imperial College, London, United Kingdom; 5 Department of Genitourinary Medicine, Northampton General Hospital, Northampton, United Kingdom; 6 Department of Sexual Health, Royal Berkshire Hospital, Reading, United Kingdom; 7 INC, Oakleigh, Victoria, Australia; 8 Ragon Institute of Massachusetts General Hospital, Massachusetts Institute of Technology and Harvard, Boston, Massachusetts, United States of America; 9 Global Biosolutions, Craigieburn, Victoria, Australia; 10 Department of International Health, Immunology and Microbiology, University of Copenhagen, Copenhagen, Denmark; 11 KwaZulu-Natal Research Institute for Tuberculosis and HIV, K-RITH, University of Kwazulu-Natal, Durban, South Africa; Rush University, United States of America

## Abstract

**Background:**

Preclinical studies of overlapping 15mer peptides, spanning SIV, SHIV or HIV, pulsed on autologous PBMC ex vivo have demonstrated high level, virus-specific T cell responses and viral suppression in non-human primates (NHP). Opal-HIV-Gag(c) consists of 120 synthetic 15mer peptides spanning Clade C, consensus Gag, manufactured to current good manufacturing practice; having been evaluated in a good laboratory practice toxicology study in *Macaca mulatta.* We evaluated the safety and preliminary immunogenicity of such peptides administered intravenously after short-duration *ex vivo* incubation, to HIV-positive adults on suppressive antiretroviral therapy.

**Methods and Findings:**

A first-in-human, placebo-controlled, double-blind, dose escalation study was conducted. Twenty-three patients with virus suppressed by antiretroviral therapy were enrolled in four groups 12 mg (n = 6), 24 mg (n = 6), 48 mg (n = 2) or matching placebo (n = 8). Treatment was administered intravenously after bedside enrichment of 120 mL whole blood for white cells using a closed system (Sepax S-100 device), with ex vivo peptide admixture (or diluent alone) and 37°C incubation for one hour prior to reinfusion. Patients received 4 administrations at monthly intervals followed by a 12-week observation post-treatment. Opal-HIV-Gag(c) was reasonably tolerated at doses of 12 and 24 mg. There was an increased incidence of temporally associated pyrexia, chills, and transient/self-limiting lymphopenia in Opal-HIV-Gag(c) recipients compared to placebo. The study was terminated early, after two patients were recruited to the 48 mg cohort; a serious adverse event of hypotension, tachycardia secondary to diarrhoea occurred following a single product administration. An infectious cause for the event could not be identified, leaving the possibility of immunologically mediated product reaction.

**Conclusions:**

A serious, potentially life-threatening event of hypotension led to early, precautionary termination of the study. In the absence of a clearly defined mechanism or ability to predict such occurrence, further development of Opal-HIV-Gag(c) will not be undertaken in the current form.

**Registration:**

ClinicalTrials.gov NCT01123915; EudraCT: 2008-005142-23

## Introduction

HIV remains a significant global health problem, despite the availability of a range of antiretroviral treatments and strategies. An estimated 2.7 million people were newly infected with HIV and approximately 1.8 million died from HIV/AIDS in 2011 [1]. The use of combination highly active antiretroviral therapy (HAART) has significantly improved prospects for HIV infected individuals and has lowered transmission rates. However, the treatment regimens are complex, expensive and may be associated with treatment-limiting side effects and the emergence of drug resistant viral strains. These factors remain critical barriers to the management of HIV/AIDS, particularly in economically disadvantaged communities. The availability of an immunotherapy, which either delays the introduction of HAART or complements treatment by HAART, would be an important advance in treating HIV.

The induction of HIV-specific T-cell responses is critical to effective control of viraemia and delaying subsequent progression to AIDS [2]–[4]. Cytotoxic T-lymphocyte responses to the HIV structural protein Gag have been consistently associated with low viral load [5], [6], with evidence that viral escape from Gag-specific T-cell responses occurs at the expense of viral fitness [5], [7]–[9]. This suggests that Gag-specific cellular immune responses may be an appropriate target antigen for an HIV therapeutic vaccine.

Overlapping peptide-pulsed autologous lymphocytes (OPAL) is a novel immunotherapy being developed for the treatment of HIV. The therapy involves pulsing autologous peripheral blood mononuclear cells (PBMCs), enriched white blood cells (WBCs) or whole blood *ex vivo*, with a mixture of synthetic 15mer peptides overlapping by 11 amino acids. This approach, used in non-human primates, induced high-frequency, broad, polyfunctional CD4+ and CD8+ SIV-specific T cell responses [7]–[10] that resulted in a sustained, 10-fold reduction in SIV viral load in vaccinated animals [10], [11].

The vaccine for clinical evaluation, Opal-HIV-Gag(c), was manufactured to match the Gag clade C Durban consensus sequence [6]. The clade C subtype circulates in Southern Africa, India, and China, and is responsible for over 50% of all HIV infections worldwide [12]. Since Gag is highly conserved across clades, it was reasoned that Opal-HIV-Gag(c) would have broad cross-clade reactivity [11], [13]–[15].

This phase I study was the first step to determine whether the immunotherapy Opal-HIV-Gag(c) might have utility as a treatment for HIV when administered *ex vivo* to enriched WBCs. The aim was to evaluate the safety of the study vaccine, Opal-HIV-Gag(c) compared to placebo, at three dose concentrations. The secondary aim was to evaluate the immunogenicity in T-cells and to assess the impact of Opal-HIV-Gag(c) on HIV infection. The study was terminated early for safety reasons.

## Methods

The protocol for this trial and supporting CONSORT checklist are available as supporting information; see Checklist S1 and Protocol S1.

### Ethics Statement and Regulatory Approvals

The protocol, designated Opal-HIV-1001 and titled “A phase 1, dose escalating, single centre, double blind study of the safety and immunogenicity of Opal-HIV-Gag(c) in HIV-1 positive subjects” was sponsored by Medicines Development, Melbourne, Australia. The study was conducted in compliance with the International Conference on Harmonisation Good Clinical Practice, the Declaration of Helsinki, and was registered with EudraCT 2008-005142-23 prior to enrolment of participants. Ethical approval was granted by the The Royal Marsden Ethics Committee of the National Research Ethics Service, UK. All study participants voluntarily provided written informed consent before any study procedures were undertaken.

### Objectives

The primary objective was to assess the safety of Opal-HIV-Gag(c) at three dose concentrations compared to placebo in HIV-1 individuals receiving stable HAART, while the secondary objectives were to evaluate the immunogenicity and impact of Opal-HIV-Gag(c)) treatment on HIV-1 infection.

### Study Design and Participants

This was a phase I, first-in-human, double blind, placebo controlled, randomised, dose escalation study conducted at a single centre, Chelsea & Westminster Hospital, London, UK from May 2010 to October 2011. Participants were recruited from the ambulatory clinics at Chelsea & Westminster Hospital, London UK, Royal Berkshire Hospital, Reading UK and Northamptonshire General Hospital, Northampton, UK. After discussion and written confirmation of informed consent, eligibility was confirmed according to the following major criteria: aged 18–60 years, inclusive; HIV-1 infected; receiving stable antiretroviral therapy with at least 3 active drugs for a minimum of 2 months and with undetectable viral load for 6 months prior to planned study baseline; CD4+ T-cell counts >350 cells/mm^3^ with a nadir >100 cells/mm^3^, and a positive *ex vivo* or 10 day cultured IFNγ ELIspot assay to Opal-HIV-Gag(c) peptides. Exclusion criteria included: infection with hepatitis B or C; an AIDS defining condition within 42 days of Baseline; having received any immunomodulatory agents/vaccine within 60 days or any blood products within 6 months of Screening. The full inclusion/exclusion criteria for the study can be found in protocol S1.

The study protocol planned to enrol a total of up to 27 patients in three sequentially completed, ascending-dose cohorts of 9 patients each. Within each cohort, patients were randomised to receive Opal-HIV-Gag(c) (n = 6) or placebo (DMSO, n = 3) and stratified by Clade. In addition, a sentinel cohort of one Opal-HIV-Gag(c) and one placebo patient were dosed prior to the balance of the cohort. The randomisation code was computer generated and prepared by an independent statistician. Participants received doses at 4 weekly intervals on Day zero and weeks 4, 8 and 12 followed by a 12 week post-treatment follow-up safety period. Dose escalation was permitted after all participants at each dose level completed at least 2 doses and the safety data were reviewed by the independent Data Safety Monitoring Board. Patients were monitored throughout the study for adverse events, concurrent medications, physical findings, vital signs, immunological markers, viral load, CD4+ T cell counts, and safety bloods.

Clinical trial monitoring was performed by AptivSolutions, Hemel Hempstead, UK; biochemistry, haematology, viral load and T-cell enumeration was conducted by The Doctor’s Laboratory, London, UK; HLA genotyping was conducted the University of Oklahoma Health Sciences Centre, OK, USA; data management and statistical analysis for the study was performed by iNC Research, Oakleigh, Australia, and; immunogenicity was evaluated at Imperial College, London, UK and Oxford University, Oxford, UK.

### Interventions and Vaccines

Opal-HIV-Gag(c) consists of 120 peptides of 15 amino acids in length overlapping the preceding proceeding peptide by 11 amino acids. The 120 peptides span the HIV-1 Durban 2005 Clade C Gag protein consensus sequence and were manufactured to current Good Manufacturing Practice as defined by United States Part 21 Code of Federal Regulations by CS Bio, Inc. (San Mateo, CA). All 120 peptides were mixed in equal weight quantities, lyophilised and terminally gamma irradiated. A repeat dose Good Laboratory Practice toxicology study was completed in non-human primates prior to initiation of this study.

Opal-HIV-Gag(c) was reconstituted prior to administration in dimethyl sulfoxide (DMSO) United States Pharmacopoea (WAK Chemie GmbH, Germany). DMSO at the identical concentration was used as the placebo.

Opal-HIV-Gag(c) and diluent were stored frozen at −20°C±5°C in an entry restricted and temperature-monitored facility at the study site and thawed immediately prior to use, with doses prepared individually.

Opal-HIV-Gag(c) or placebo were administered by drawing 120 mL of whole blood, enriching the white blood cells into a resultant 20 mL volume using a Sepax S-100 cell separation device (Biosafe SA, Switzerland) adding Opal-HIV-Gag(c) and were incubated at 37°C for one hour, prior to intravenous re-infusion. The concentration of DMSO in the reinfusion was 4% for both Opal-HIV-Gag(c)) or placebo.

The clinical doses were selected based on the non-human primate efficacy and safety studies. In the repeat dose toxicology study in non-human primates, the maximum dose was 5 doses of 18.5 mg Opal-HIV-Gag(c), which is the equivalent of 74 mg/square metre (m^2^) of Body Surface Area (BSA) (assumes 0.25 m^2^ BSA for a monkey of 3 kilograms [kg]), or 6.2 mg/kg at each of the 5 administrations and this was also determined to be the no observable adverse effect level (NOAEL). The clinical starting dose was 12 mg or 7.4 mg/m^2^ (0.2 mg/kg, assuming 1.62 m^2^ BSA for a 60 kg human) at each of four administrations and the maximum dose proposed in the clinical trial was 48 mg or 29.6 mg/m^2^ (0.8 mg/kg). Hence, the first-in-human starting dose for the study was approximately 10 times lower than the NOAEL in non-human primates (based on the USA Food and Drug Administration Guidance for Industry and Reviewers Estimating the Safe Starting Dose in Clinical Trials for Therapeutics in Adult Healthy Volunteers [9]). Though the patients recruited to this study were infected with HIV-1, the eligibility criteria were suitably defined such that their health status (on stable antiretroviral therapy) could otherwise reasonably be classified as healthy adult participants.

### Immunogenicity

The induction of T-cell immunogenicity was assessed by using *ex vivo* ELISpot responses using Opal-HIV-Gag(c), Tat, Rev, Nef, a mock as a negative control and cytomegalovirus peptides to stimulate T-cells in accordance with the laboratories’ standard procedures.

### Sample Size and Statistical Design

The methodology for reporting study data was detailed in the Statistical Analysis Plan (SAP). Appropriate descriptive statistics for the data were determined using SAS (version 9.2 - SAS Institute Inc., Cary, North Carolina, USA). Adverse events were coded according to the Medical Dictionary for Regulatory Activities (MedDRA) (version 12.0). Concomitant medications were coded using the latest version (Quarter 2, 2010) of the World Health Organisation (WHO) Drug coding dictionary. All data compiled for participants prior to the point of discontinuation has been used for analyses with all withdrawals being included in analysis up to the time of withdrawal regardless of duration of treatment. No substitutions were made for missing data. All analyses were based on available data, unless otherwise stated. Blinded interim reviews of immunogenicity data (to W14) were conducted after the 12 mg and 24 mg Cohorts were completed.

All statistical analyses were carried out using two sided tests at the 5% level of significance. In cases where the parameters did not follow a normal distribution, log transformations were used. If the log transformed data was not normally distributed, a non-parametric test (Kruskal-Wallis) was used to analyse the difference in population medians.

The sample size for the study was selected based on industry guidance and Phase I study design [13]–[16] and, as such, no formal sample size calculation was performed for this study.

## Results

### Study Population

Twenty three patients satisfied the inclusion and exclusion criteria and were randomised to receive 12 mg (n = 6), 24 mg (n = 6), 48 mg (n = 2) or placebo (n = 9, with only 8 being analysed after receiving intervention). Five participants withdrew from the study: one patient, allocated to receive placebo, due to equipment failure prior to treatment administration (this patient was replaced); 1 receiving 48 mg withdrew due to a serious adverse event (SAE) leading to early study termination; and three patients (n = 1 48 mg, n = 2 placebo) were required to withdraw when the study was terminated. In addition, one patient with elevated ALT due to concurrent therapy withdrew from treatment but remained on the study. Despite the small number of patients, the demographic characteristics of the cohorts were not markedly different (Table S1).

### Impact of OPAL-HIV-Gag(c) on HIV Viral Load and Absolute CD4 Counts

HIV-1 viral load remained well controlled, without any result, confirmed by repeat, above 50 copies/ml for all study patients throughout the study period (not shown), indicating an absence of viral rebound. In all cohorts, CD4+ cell count varied around a stable plateau, consistent with the pattern of variability seen in normal clinical follow-up and with no temporal association with administration of Opal-HIV-Gag(c)) or placebo (data not shown).

### Safety Analyses

Opal-HIV-Gag(c) was generally well tolerated. The most commonly occurring AEs (ranked on total number of participants in all treatment cohorts experiencing AE) are presented in Table S2. At the 12 mg or 24 mg cohorts, there was no evidence of an increasing incidence or severity of adverse events with increasing dose and there was a similar number of events in patients receiving Opal-HIV-Gag(c) or placebo. Body temperature increases were temporally associated with Opal-HIV-Gag(c), regardless of dose, and there was an increased incidence of rigors, chills and transient lymphopenia temporally associated with Opal-HIV-Gag(c) but not placebo treatment.

The second patient randomised to receive 48 mg Opal-HIV-Gag(c) experienced a treatment and study-terminating SAE. The event was comprised of hypotension, tachycardia, diarrhoea, and anuria. The patient, a 53-year-old male originally from Kenya, was infected with HIV-1 clade B/D virus since 2006 and had achieved virologic suppression with tenofovir, emtricitabine and efavirenz since shortly after diagnosis. Pre-treatment, he reported good health with no clinically relevant abnormalities detected on full physical examination or in baseline laboratory values. Within 2 hours of completion of the first infusion, he began to experience cramping, abdominal discomfort and subsequently passed large volume, watery bowel motions on 3 occasions within the ensuing hour, with simultaneous vomiting on two occasions. There was no blood present in either stool or vomit, and examination revealed a quiescent abdomen following evacuation with no evidence of rash or angioedema. Though afebrile during this period, the fluid losses resulted in a hypovolaemic state with a drop in systolic blood pressure, tachycardia and tachypnoea. No specific therapy was administered to manage any presumed cause of this event, with only intravenous fluid replacement and low molecular weight heparin to prevent venous thrombosis. The patient made a rapid and full recovery and was discharged from hospital on day 5 after administration of investigational product, with resolution of hypotension, tachycardia, diarrhoea and anuria.

Stool cultures were negative for cryptosporidia, *C. difficile*, *E.coli* 0157, *Salmonella*, *Shigella*, *Campylobacter*. No ova, cysts or parasites were detected on microscopic examination and ELISA for viruses (adenovirus, norovirus and rotavirus) were negative. There was no growth from blood or urine cultures and no plasmodium were visible on blood film on examining for the presence of malaria. Both polymerase chain reaction (PCR) for rotavirus and norovirus on stool sample and *C.perfringens* enterotoxin test on stool were also negative. There was no rise from baseline to 24 hours post admission in mast cell tryptase thus reducing the likelihood of diagnosis of an anaphylactoid-type reaction. Lymphopenia was reported 4 hours post-dose, but had resolved to within normal reference range within 2 days.

### Immunogenicity

Comparisons between treatment groups for immunogenicity by *ex vivo* IFN-γ ELISpot showed no overall difference for any of the parameters tested (Opal-HIV-Gag(c), Mock, Rev, Tat and Nef) compared to Baseline or placebo. There was an apparent response in area under the curve (AUC) for Rev (p = 0.012 before correction for multiple tests) for the 12 mg Opal-HIV-Gag(c) group compared with pooled placebo participants (n = 8) (data not shown; these data are described more fully in a separate manuscript), but this was not significant after Bonferroni correction for multiple tests. Individually, there were two participants, one in each of the 12 mg and 24 mg Opal-HIV-Gag(c) cohorts, who responded at W14 (2 weeks post-treatment) compared to Baseline (data not shown; these data are described more fully in a separate manuscript).

## Discussion

This was a first-in-human Phase I, double-blind, placebo-controlled, dose-escalation study of the safety and immunogenicity of Opal-HIV-Gag(c) at 3 dose levels in patients with well controlled HIV-1 infection. The initial clinical trial program planned for the Opal vaccine was in two initial steps. The current study was designed as a safety evaluation in patients with well-controlled viral replication on antiretroviral therapy and without an interruption to therapy. Subsequent to this study, it was intended to evaluate efficacy in a Phase I/II study in an adult and then in a paediatric population where efficacy could be established in a treatment interruption model.

The study escalated through 12 mg and 24 mg before being terminated due to a SAE in the 48 mg cohort. Although the sentinel patient allocated to receive the active product had tolerated 48 mg without any notable side effects, the second patient’s hypotension and anuria secondary to diarrhoea and vomiting occurring approximately 2 hours after dosing led directly to Sponsor and Investigator-agreed clinical hold. The thorough investigation included the vaccine, administration method, and the patient’s recent and past medical history. An identifiable cause for the event in standard areas of evaluation (infectious agent/food poisoning, co-morbidities, medical cause other than the study product, study product not meeting specification and or the study procedures not being followed) could not be identified. The incidence of gastrointestinal findings in all other patients between Opal-HIV-Gag(c) and placebo recipients was similar, and there was no evidence of immunotoxicity in the GLP toxicology study conducted in *Macaca mulatta*.

As a result of the failure to identify an alternative causative agent or to identify methods of ameliorating the event should it occur again in other patients, the study was terminated as a safety precaution.

In all other patients, Opal-HIV-Gag(c) was well tolerated. Consistent with many phase I studies, headache was the most commonly reported AE in this study and occurred at similar rates in patients receiving either Opal-HIV-Gag(c) or placebo. This may be associated with the study requirements for fasting and caffeine withdrawal. Consistently observed, was an increased incidence of fever, rigor, headache and transient, self-limiting lymphopenia in patients receiving Opal-HIV-Gag(c) at any dose but not placebo recipients. The temporal association of these events with Opal-HIV-Gag(c) is consistent with an innate immune response and provides evidence of a biological response to the peptides rather than the *ex vivo* administration method or diluent [16], [17].

Adverse events frequently reported in the literature for DMSO (e.g. sedation, headache, facial flushing [17]–[19], nausea, vomiting, abdominal cramps, dizziness [18]–[20] and a taste of garlic or onion [20]–[22] were not observed in this study.

*Ex vivo* white blood cell enrichment conducted bed-side in the closed system Biosafe Sepax S-100 device was employed as a more practical alternative to a laboratory based PBMC separation methodology. Prior to the conduct of this study, a separate pilot study was conducted in 6 patients to evaluate the equipment. In clinical use, the equipment failed on a number of occasions, limiting its potential for use as a real-time tool for WBC enrichment.

The translation from animal models to humans remains problematic in HIV vaccinology with this study non-predictive for immunogenicity, safety and efficacy between non-human primates and humans [21], [22]. The absence of a clear immunogenic signal in this study is in marked contrast to the significant T-cell immunogenicity observed in studies in *Macaca nemestrina* with the Opal vaccination methodology [1], [10]. A GLP, repeat dose, non-human primate (Macaca mulatta) toxicology study was conducted to evaluate the safety and immunotoxicity of Opal HIV Gag(c). Eighteen animals were randomly allocated to receive 1.85 or 18.5 milligram (mg) of cGMP Opal HIV Gag(c) in DMSO or DMSO only (n = 6 per group). Treatment was added to whole blood ex vivo, incubated for 1 hour at 37 degrees (°) Celsius (C), and reinfused on 5 separate occasions. There were no clinically relevant adverse findings in either Opal HIV Gag(c) or control animals with all animals remaining healthy throughout the study. There were no treatment related changes in haematology, serum chemistries or urinalysis, and no relevant histological findings upon necropsy.

The exposure to peptides, and the number of cells exposed to peptides ex vivo were both within the range or greater than that used in the *Macaca nemestrina* studies (0.035 to 0.07 mg peptides per million WBC in non-human primates compared to 0.018 to 0.285 mg peptides per million WBC in this study). In determining the adequacy of the clinical dose for immunogenicity purposes, both the amount of Opal HIV Gag(c) and the number of PBMCs exposed have been taken into account. Allometric scaling on body surface area (BSA) was performed to determine the blood volume required in the clinic to yield a proportionally similar number of PBMCs to that shown to be effective in macaques. The blood volume ranged from 29.5 mL to 120 mL, and thus 120 mL of venous blood was drawn from each patient. From the non-human primate non-clinical studies, it was estimated that 9×106 to 1.8×107 PBMCs were exposed to peptides at each administration for each macaque. Allometrically scaling this to humans (based on BSA) yielded an ideal PBMC count of 5.9×107 to 1.2×108 for Opal treatment per person, the range achieved in the clinical trial. Finally, a range of 0.035 mg to 0.07 mg of peptide per million PBMCs was shown to be efficacious in the non-human primate model. Extrapolating this to humans with the expected PBMC yield from 120 mL of whole blood requires a range of minimum doses of 4.2 to 16.8 mg per administration. The clinical doses evaluated span this range with the minimum dose of 12 mg and the maximum dose of 48 mg.

In conclusion, in this double blind, placebo controlled, dose escalation study, Opal-HIV-Gag(c)) was generally well tolerated in adults with well-controlled HIV-1 infection at doses of 12 and 24 mg. There was an increased incidence of pyrexia, chills, rigor, and transient (and self-limiting) lymphopaenia in Opal-HIV-Gag(c) recipients compared to placebo. There were no clear differences in dose on the incidence of laboratory abnormalities or the nature, incidence or severity of adverse events. There was no evidence of a treatment effect on T-cell responses, measured by *ex vivo* ELISpot, after administration of Opal-HIV-Gag(c).). An SAE of life-threatening hypotension at a dose of 48 mg lead to early, precautionary termination of the study. Further development of OPAL will not be undertaken in the current form.

## Supporting Information

Table S1 **Patient characteristics.**
(DOCX)

Table S2 **Summary of common adverse events.**
(DOCX)

Protocol S1 **Trial protocol.**
(PDF)

Diagram S1 **CONSORT Flow diagram.**
(PPTX)

Checklist S1 **CONSORT checklist.**
(DOCX)
