# Supplementary material for: A Randomised, Placebo-Controlled, First-In-Human Study of a Novel Clade C Therapeutic Peptide Vaccine Administered Ex Vivo to Autologous White Blood Cells in HIV Infected Individuals
Source: PLoS One. 2013 Sep 17;8(9):e73765. doi: 10.1371/journal.pone.0073765 (PMC3775760; doi:10.1371/journal.pone.0073765)
Supplement: Table S1 — Patient characteristics. (DOCX) [file pone.0073765.s001.docx]

Table S1. Patient characteristics

| **Parameter** | **Active [Opal-HIV-Gag(c)]** | | | **Placebo**  n = 8 |
| --- | --- | --- | --- | --- |
|  | **12mg**  n = 6 | **24mg**  n = 6 | **48mg**  n = 2 |  |
| **Age** in years, **median** (*min, max)* | **43.5** *(32,56)* | **35.0** *(28,43)* | **50.0** *(46,54)* | **46.0** *(25,56)* |
| **Gender** male:female | 4:2 | 4:2 | 2:0 | 5:3 |
| **Ethnicity** (n) |  |  |  |  |
| American Hispanic | 0 | 0 | 0 | 0 |
| Asian | 0 | 1 | 0 | 1 |
| Black | 4 | 2 | 1 | 4 |
| White | 1 | 2 | 1 | 4 |
| Other | 1 | 1 | 0 | 0 |
| **Weight** in kg, **mean** (*standard deviation*) | **77.3** (*12.1*) | **90.5** (*33.9*) | **118.1** (*7.5*) | **80.0** (*9.3*) |
| **Median** (*min, max*) | **81.7** (*55.2, 86.8*) | **77.1** (*58.4, 150.8*) | **118.1** (*112.8, 123.4*) | **76.9** (*69.8, 100.4*) |
| **HIV clade subtype of infection** |  |  |  |  |
| HIV clade C | 3 | 3 | 1 | 5 |
| HIV clade B | 1 | 3 | 0 | 3 |
| Other | 2 (B/F,B/D & A) | 0 | 1 (B/D) | 0 |
| **Years since HIV diagnosis** in years, **median** (*min, max*) | **7.0** (*3.0,9.0*) | **7.0** (*2.0,12.0*) | **11.0** (*5.0, 17.0*) | **8.5**  (*5.0, 12.0*) |
