# Supplementary material for: A Randomised, Placebo-Controlled, First-In-Human Study of a Novel Clade C Therapeutic Peptide Vaccine Administered Ex Vivo to Autologous White Blood Cells in HIV Infected Individuals
Source: PLoS One. 2013 Sep 17;8(9):e73765. doi: 10.1371/journal.pone.0073765 (PMC3775760; doi:10.1371/journal.pone.0073765)
Supplement: Table S2 — Summary of common adverse events. (DOCX) [file pone.0073765.s002.docx]

Table S2: Summary of common adverse events.

|  | Opal-HIV-Gag(c) | | | |
| --- | --- | --- | --- | --- |
| Treatment Emergent Adverse Events | 12 mg n=6 | 24 mg n=6 | 48mg n=2 | Placebo n = 8 |
|  | Number of subjects (number of events) | | | |
| Headache | 4 (9) | 4 (9) | 0 (0) | 3 (8) |
| Pyrexia | 2 (5) | 1 (1) | 2 (4) | 0 (0) |
| Diarrhoea | 0 (0) | 0 (0) | 1 (1) | 3 (3) |
| Lethargy | 1 (1) | 2 (3) | 0 (0) | 1 (1) |
| Myalgia | 1 (2) | 2 (4) | 0 (0) | 1 (1) |
| Tachycardia | 1 (1) | 1 (1) | 2 (2) | 0 (0) |
