# Supplementary material for: A Randomised, Placebo-Controlled, First-In-Human Study of a Novel Clade C Therapeutic Peptide Vaccine Administered Ex Vivo to Autologous White Blood Cells in HIV Infected Individuals
Source: PLoS One. 2013 Sep 17;8(9):e73765. doi: 10.1371/journal.pone.0073765 (PMC3775760; doi:10.1371/journal.pone.0073765)
Supplement: Protocol S1 — Trial protocol. (PDF) [file pone.0073765.s003.pdf]

Clinical Study Protocol

**Study Title:**	A phase 1, dose escalating, single centre, double blind study of the safety and immunogenicity of Opal-HIV-Gag clade C in Human Immunodeficiency Virus-type 1 (HIV-1) positive subjects.			
**Sponsor:**	Medicines Development Limited<br>Level 1, 18 Kavanagh St<br>Southbank, Victoria, 3006, Australia<br>Phone: +61 3 9629 6111<br>Fax: +61 3 9629 8718			
**Protocol ID:**	Opal-HIV-1001			
**Medical Monitor:**	Dr Mark Atkins MB BS, BSc, MSc, FRCPath			
**Protocol Version / Date:**	Final:	Final	Date:	18 Sep 2009
	Amendment	one	Date:	09 Feb 2010
	Amendment	two	Date:	18 Aug 2010
	Amendment	three	Date:	28 June 2011

CONFIDENTIALITY STATEMENT

This study is being performed in compliance with the guidelines of Good Clinical Practice (GCP) and all essential documents are being archived.

The information contained in this document, particularly unpublished data, is the property of, or under the control of Medicines Development Limited (“MDL”), and is provided to you in confidence as an Investigator, potential Investigator, or consultant, for review by you, your staff, and an applicable Institutional Review Board or Independent Ethics Committee. The information is only to be used by you in connection with authorised clinical studies of the investigational medicinal product described in the protocol. You will not disclose any of the information to others without written authorisation from MDL, except to the extent necessary to obtain informed consent from those persons to whom the investigational medicinal product may be administered.

MEDICINES DEVELOPMENT LIMITED

STUDY ACKNOWLEDGEMENT

A phase 1, dose escalating, single centre, double blind study of the safety and immunogenicity of Opal-HIV-Gag clade C in HIV-1 positive subjects

Opal-HIV-1001, Version final incorporating Amendment 3, dated 28 Jun 2011

This protocol has been approved by Medicines Development Limited. The following signature documents this approval.

Mark Sullivan	
Name (Printed)	Signature
	29 Jun 2011
	Date (dd mmm yyyy)

INVESTIGATOR STATEMENT

I have read the protocol, including all appendices, and I agree that it contains all necessary details for me and my staff to conduct this study as described. I will conduct this study as outlined herein and will make a reasonable effort to complete the study within the time designated.

I will provide all study personnel under my supervision with copies of the protocol and access to all information provided by Medicines Development Limited. I will discuss this material with them to ensure that they are fully informed about the investigational medicinal product and the study.

Principal Investigator's Name (Printed)	Signature
Site number	Date (dd mmm yyyy)

1 STUDY SYNOPSIS

**Protocol No.:**	Opal-HIV-1001
**Study Title:**	A phase 1, dose escalating, single centre, double blind study of the safety and immunogenicity of Opal-HIV-Gag clade C in HIV-1 positive subjects.
**Name of Study Product:**	Opal-HIV-Gag(c)
**Indication:**	Human Immunodeficiency Virus (HIV)
**Development Phase:**	Phase 1
**Rationale:**	There is a need for therapeutic approaches that induce or enhance T-cell immunity to control HIV disease. While antiretroviral therapy (ART) has reduced morbidity and mortality of HIV/Acquired Immunodeficiency Syndrome (AIDS), cost, development of resistance and side effects remain limitations. Most therapeutic HIV vaccine studies to date have been hampered either by inefficient induction of relevant immune responses and/or a requirement for extensive and logistically difficult cell manipulations *ex vivo*.<br>Overlapping Peptide-pulsed Autologous Cells (Opal) is a technique where autologous peripheral blood mononuclear cells (PBMC) or whole blood is pulsed with sets of overlapping 15 amino acid long (15mer) peptides spanning whole proteins of HIV. This technique:<br>• has stimulated broadly reactive, high level CD4 positive (CD4+) and CD8 positive (CD8+) T-cell immune responses in macaques<br>• is simple to administer (no manipulation or selection of antigen presenting cells is required)<br>• is well tolerated in macaques<br>• is not predicated on prior knowledge of Major Histocompatibility Complex (MHC) genotype of the host or T-cell epitopes of the pathogen.
**Primary objective:**	Assess the safety of Opal-HIV-Gag(c) at 3 concentrations compared to placebo in HIV-1 infected subjects.
**Secondary objectives:**	Evaluate the immunogenicity of Opal-HIV-Gag(c), and preliminarily assess the impact of Opal on HIV-1 infection.
**Study design:**	Randomised, double blind, placebo-controlled, dose escalation study.
**Number (n) of participants:**	27
**Number of centres:**	1 centre
**Inclusion criteria:**	• Provision of written informed consent<br>• Documented laboratory diagnosis of HIV-1 infection (either positive Enzyme-Linked ImmunoSorbent Assay (ELISA) HIV-1 antibody test confirmed by Western Blot, p24 assay, HIV ribonucleic acid (RNA), or culture)<br>• Documented HIV subtype (clade) of infection<br>• 18 - 60 years of age, inclusive<br>• Stable ART regimen containing at least 3 active ART agents for at least 2 months prior to Baseline<br>• Plasma HIV-RNA <400 copies/millilitre (mL) for 6 months up to and including Screening. Subjects on stable ART with a single value ≥400 copies/mL (i.e. the result is unconfirmed by subsequent testing) within this timeframe may be included at the discretion of the Investigator<br>• CD4+ T-cell count ≥350 cells/cubic millimetre (mm^3) at screening (with nadir ≥100 cells/mm^3)<br>• A positive immunogenic response when stimulated with HIV-1 Gag clade C peptides at Screening<br>• Male or female. Women of child-bearing potential must be using two effective methods of contraception and agree to continue to do so from

	Screening, throughout study medication dosing and for 28 days after the last dose of study medication.
**Exclusion criteria:**	• Any serious or active medical or psychiatric illness which, in the opinion of the Investigator, would interfere with treatment, assessment, compliance with the protocol, or subject safety. This would include any active, clinically significant renal, cardiac, pulmonary, vascular, or metabolic (thyroid disorders, adrenal disease) illness, or malignancy<br>• Hepatitis B surface antigen (HBsAg) or both Hepatitis C Virus (HCV) antibody and HCV RNA positive at Screening<br>• Female subjects who are lactating and those of reproductive potential with a positive urine beta human chorionic gonadotropin (β-HCG) pregnancy test at either Screening or Baseline visit<br>• A new AIDS-defining condition diagnosed within 42 days prior to Baseline visit<br>• Known or suspected allergy to Dimethyl Sulfoxide (DMSO). History of allergy or reaction to medications (including peptide or protein containing agents) or history of severe allergy that, in the opinion of the Principal Investigator (PI), might compromise the subject's participation in any way<br>• Moderate or severe asthma, defined as at least chronic moderate symptoms which frequently interfere with daily activities and require anti-asthma/anti-inflammatory agents<br>• Have received immunomodulating agents (including immunosuppressive agents, interferon or other immune or cytokine-based therapies), immunisation, and/or systemic chemotherapeutic agents within 60 days of Screening or expected to receive these agents during the course of the study<br>• Recipient of live attenuated vaccines within 60 days of Screening<br>• Recipient of whole killed, toxoid or sub-unit vaccines (e.g. influenza, pneumococcus, tetanus, hepatitis B) within 42 days prior to Baseline<br>• Ever received an HIV prophylactic or immunotherapeutic vaccine (does not apply to subjects who have written documentation of receiving placebo or adjuvant only)<br>• Recreational and/or therapeutic drug or alcohol use that, in the opinion of the PI, might compromise the subject's participation in any way. Subject's urine will be tested for illicit drugs at Screening and entry into the study will be at the discretion of the PI (see Section 5.4)<br>• Medical or psychiatric condition or occupational responsibilities that may preclude compliance with the protocol<br>• Laboratory blood values:<br>o Haemoglobin <11.0 grams/decilitre (g/dL) for men and <10.0 g/dL for women<br>o Neutrophil count $<800/mm^3$<br>o Platelet count $<50,000/mm^3$<br>o Aspartate aminotransferase (AST) or alanine aminotransferase (ALT) >2.5 times the upper limit of normal (ULN)<br>o Lipase >2.5 times ULN<br>o Amylase >1.5 times ULN (unless serum lipase is ≤1.5 times ULN)<br>o Subjects with an estimated creatinine clearance of <80 mL/minute (min)<br>• Recipients of blood products or immunoglobulins within 6 months prior to Screening, or loss of 450 mL or more of blood during the three months prior to Screening<br>• Recipients of experimental or investigational agents within 30 days prior to Screening<br>• Previous participation in this study
**Study product:**	Opal-HIV-Gag(c) manufactured to current Good Manufacturing Practice (cGMP). Opal-HIV-Gag(c) is a white lyophilised powder with 60 mg of powder in each vial. Opal-HIV-Gag(c) is reconstituted with DMSO – European

	Pharmacopoeia (Ph Eur)/ United States Pharmacopeia (USP).
**Comparator:**	Diluent only (DMSO)
**Duration of treatment per participant:**	Screening period: 14 to 42 days prior to Baseline
	Primary Treatment period: 12 weeks
	Continued Treatment period: To Week (W) 24
**Study procedures:**	Subjects will provide voluntary written informed consent and be screened for eligibility. Eligible subjects will return at Baseline (Day [D] 0) to be randomised to receive their first treatment.<br>Subjects will be required to remain in the clinic for twenty four hours after the administration of IMP at W0 and 4. Two further treatments will be given, one each at W8 and 12. Subjects will remain in the clinic for a minimum of 8 hours after administration of IMP.<br>Subjects will be followed up through clinic visits at each of the assessment visits at the end of D3 and W2, 6, 10, 13, 14, 16 and 24.<br>Adverse events (AEs), clinical laboratory parameters (haematology, chemistry) and concurrent medications will be recorded throughout the study.<br>Immunogenicity will primarily be assessed at W14 with plasma and PBMC stored at selected clinic visits.
**Contraindications to further dosing:**	Treatment related Grade 3 or 4 toxicity.
**Safety parameters:**	Refer to Table 1
**Clinical procedures / assessments:**	Refer to Table 1
**Specialised analyses:**	T-cell immunogenicity will be primarily assessed by interferon gamma Enzyme-Linked Immunosorbent Spot assay (ELISpot) at Baseline and W13 and 14 (1 and 2 weeks after the last vaccination). Peptides assessed will be pools of 15mers overlapping by 11 amino acids and spanning C clade Gag Durban Consensus sequence.<br>Other exploratory immunological assays may be performed (such as viral suppression and intracellular cytokine staining).
**Sample size determination:**	This is a first in human phase I study with the primary objective of assessing safety. As such, no formal statistical hypothesis will be tested. Twenty seven subjects will be included in the study, including 18 active and 9 placebo. A total of 18 subjects receiving active treatment will give at least reasonable power to detect severe treatment related toxicities at rates of 20% or greater.
**Statistical analyses:**	This is a first-in-man phase I study and, as such, no formal statistical hypothesis will be tested.<br>Safety will be examined through AEs, vital signs and routine laboratory screening (haematology and clinical chemistry).<br>Immunogenicity will be assessed through T-cell immunogenicity.
**Committees / guidelines:**	A Data Safety Monitoring Board (DSMB) will provide independent review of the safety data in this study.
**Special protocol requirements / issues:**	None

Table 1: Table of Assessments

Assessment	Screening	Baseline			On-Study Period																Exit
	D–42 to D–14	D0 to D1* Dose			D3	W2	W4* Dose			W6	W8* Dose			W10	W12* Dose			W13	W14	W16	W24
		Pre	Post	Discharge			Pre	Post	Discharge		Pre	Post	Discharge		Pre	Post	Discharge				
Informed Consent	X																				
Medical History	X																				
Eligibility determination	X	X																			
Randomisation		X																			
Full Physical Examination	X	(X)	(X)	(X)	(X)	(X)	(X)	(X)	(X)	(X)	(X)	(X)	(X)	(X)	(X)	(X)	(X)	(X)	(X)	(X)	X
Targeted Physical Examination		X	X	X	X	X	X	X	X	X	X	X	X	X	X	X	X	X	X	X	
Demography, Height	X																				
Body weight	X	X			X	X	X			X	X			X	X			X	X	X	X
Vital Signs	X	X	X	X	X	X	X	X	X	X	X	X	X	X	X	X	X	X	X	X	X
Electrocardiogram**	X	X	X				X	X			X	X			X	X					
Urine β-HCG pregnancy test	X	X					X				X				X					X	X
Urine drug screen	X	X					X				X				X						
HIV#/Hepatitis C virus/Hepatitis B surface antigen serology	X																				
Haematology and biochemistry	X	X	X	X		X	X	X	X	X	X	X		X	X	X		X	X	X	X
Adverse event review		X	X	X	X	X	X	X	X	X	X	X	X	X	X	X	X	X	X	X	X
Concurrent Medications review	X	X	X	X	X	X	X	X	X	X	X	X	X	X	X	X	X	X	X	X	X
Admission to the clinic		X					X				X				X						
24 hour stay		X					X														
8 hour stay											X				X						
Administration of investigational medicinal product		X					X				X				X						
HIV viral load and CD4+ T-cell count	X	X					X				X				X					X	X
Immunogenicity testing		X												X	X			X	X	X	
Response to clade C peptides	X																				
Human Leukocyte Antigen typing		X																			

(X) As clinically indicated * Visit conducted via telephone on D5, 7, 31, 59 and 87 for adverse event review and concurrent medications ** Including continuous cardiac monitoring on treatment days
If required

Table of Contents

1 STUDY SYNOPSIS III
2 INTRODUCTION....................................6
2.1 Background....................................6
2.2 Therapeutic Vaccination for HIV 7
2.3 Opal-HIV-Gag(c)....................................7
2.3.1 *In vitro* activity 8
2.3.2 *In vivo* activity 8
2.3.3 Non-clinical Toxicology....................................9
2.3.4 Metabolism and pharmacokinetics 10
2.3.5 Method of Administration 10
2.4 Clinical trials of Opal-HIV-Gag(c)....................................10
2.4.1 Opal-HIV-1002 protocol 10
2.5 Rationale for study....................................11
2.5.1 Rationale for doses selected....................................12
3 OBJECTIVES 13
4 STUDY DESIGN 14
4.1 Study Design....................................14
4.2 Dosing Regimens....................................14
4.3 Study Sites 15
4.4 Estimated Duration of the Study....................................15
5 SUBJECT POPULATION....................................16
5.1 Selection and Number of Subjects....................................16
5.2 Inclusion Criteria 16
5.3 Exclusion Criteria 16
5.4 Other Study Eligibility Criteria Considerations....................................17
6 SCHEDULE OF ASSESSMENTS AND PROCEDURES....................................19
6.1 Study Schedule of Evaluations 19
6.2 Study Procedures / Assessment Periods....................................19
6.2.1 Screening visit 19
6.2.2 Day 0 - Day 1—Baseline evaluations, randomisation, and study medication administration 20
6.2.2.1 Pre-Dose....................................20
6.2.2.2 Study medication administration....................................20
6.2.2.3 Post-dose 20
6.2.3 Day 5, 7, 31, 59 and 87 safety visits conducted by telephone 21
6.2.4 Week 4 in-patient study medication administration visit 21
6.2.4.1 Pre-dose....................................21
6.2.4.2 Study medication administration....................................21
6.2.4.3 Post-dose 21
6.2.5 Week 8 and 12 in-patient study medication administration visits 22
6.2.5.1 Pre-dose....................................22
6.2.5.2 Study medication administration....................................22
6.2.5.3 Post-dose 22
6.2.6 On-study clinic visits Day 3 and Weeks 2, 6, 10, 13, 14 and 16 23
6.2.7 Exit evaluation (Week 24) 23
6.3 Details on Scheduled Assessments 24
6.3.1 Demographic Data, Medical History, Physical Examination, Vital Signs 24
6.3.2 Electrocardiograms....................................24
6.3.3 Blood and Urine samples for laboratory tests....................................25
6.3.4 Handling and processing of biological specimens....................................25
6.3.5 Time of blood incubation and infusion activities 25

6.4 Visit windows 26
6.5 Fasting....26

7 STUDY PRODUCTS....27
7.1 Randomisation process 27
7.2 Blinding 27
7.3 Treatment Allocation 27
7.4 Method of Unblinding....27
7.4.1 Medical emergency....27
7.4.2 End of study....27
7.5 Investigational medicinal products 28
7.5.1 Formulation 28
7.5.2 Supply, packaging and labelling, storage and handling....28
7.5.3 Dosage and administration of investigational medicinal product....29
7.5.3.1 Dose of investigational medicinal product....29
7.5.3.2 Administration of investigational medicinal product....29
7.5.4 Dispensing and accountability....31

8 CONCOMITANT MEDICATIONS AND TREATMENTS....32
8.1 Special Dietary Requirements....32
8.2 Concomitant Medications / Treatments Not Permitted....32
8.2.1 Prior to study entry 32
8.2.2 During the study dosing period....32

9 ADVERSE EVENTS AND TOXICITY MANAGEMENT....33
9.1 Safety Parameters....33
9.2 Adverse Events 33
9.2.1 Assessment of Adverse Events....33
9.3 Serious Adverse Events 34
9.3.1 Clarification of Serious Adverse Events....34
9.3.2 Serious adverse event reporting requirements....35
9.3.2.1 All serious adverse events....35
9.3.2.2 Investigator reporting requirements for serious adverse events 35
9.4 Follow up of Serious and Non-serious Adverse Events....35
9.5 Clinical Laboratory Abnormalities and Other Abnormal Assessments as Adverse Events or Serious Adverse Events 36
9.6 Guidance for toxicity management....36
9.7 Warnings and Precautions....37
9.8 Risks for Women of Childbearing Potential or during Pregnancy 37
9.9 Procedures to be followed in the Event of Pregnancy 37

10 DATA SAFETY MONITORING BOARD REVIEW 38

11 SUBJECT COMPLETION / WITHDRAWAL 39
11.1 Subject Completion....39
11.2 Criteria for Premature Withdrawal from Treatment or the Study....39
11.3 Withdrawal of subjects from Study Product....39
11.4 Withdrawal of Subjects from the Study....40
11.5 Replacement of withdrawn subjects 40
11.6 Premature termination of the study....40

12 STATISTICAL ANALYSIS....41
12.1 Hypothesis 41
12.2 Sample Size Determination....41
12.3 Randomisation 41
12.4 Criteria for evaluation of study objectives....41
12.4.1 Definition of evaluation of study objective(s) 41
12.4.2 Analysis populations....42

12.4.2.1 Group comparability 42
12.4.2.2 Data analysis methods 42
12.5 Statistical analysis 42
12.6 Analyses 42
12.6.1 Week 14 Interim Analysis 42
12.6.2 Final Analysis 43
12.6.3 Analysis of demographics 43
12.6.4 Analysis of safety 43
12.6.4.1 Incidence of adverse events 43
12.6.4.2 Clinical laboratory parameters 43
12.6.4.3 Vital signs 43
12.6.5 Analysis of efficacy 43
12.6.6 Other Efficacy Analyses 43
12.6.7 Exploratory Analyses 43

13 GENERAL STUDY ADMINISTRATION 44
13.1 Ethical Aspects 44
13.1.1 Local regulations / Declaration of Helsinki 44
13.1.2 Informed consent 44
13.1.3 Premature withdrawal 44
13.1.4 Institutional Review Boards or Ethics Committees 44
13.1.5 Conditions for modifying the protocol 44
13.1.6 Conditions for terminating the study 45
13.2 Study Documentation, Case Report Forms and Record Keeping 45
13.2.1 Investigator's files / Retention of documents 45
13.2.2 Background data 45
13.2.3 Inspections 46
13.2.4 Case Report Forms 46
13.3 Monitoring the Study 46
13.4 Confidentiality of Trial Documents and Subject Records 46
13.5 Publication of Data and Protection of Trade Secrets 47
13.6 Anticipated Subject Accrual and Duration of the Study 47

14 REFERENCES 48

APPENDIX A AIDS CLINICAL TRIAL GROUP (ACTG) GRADING SCALE 50

APPENDIX B WORLD MEDICAL ASSOCIATION DECLARATION OF HELSINKI 67

APPENDIX C AMENDMENT 1 71

APPENDIX D AMENDMENT 2 75

LIST OF TABLES

Table 1: Table of Assessments vi
Table 2: Laboratory Parameters 25
Table 3: Dose of investigational medicinal product in 20 mL WBC-enriched blood component 29
Table 4: Grading Scale where AIDS Clinical Trial Group Toxicity Grades Do Not Apply 34
Table 5: Toxicity management. 36

LIST OF FIGURES

Figure 1: Study design 14

GLOSSARY OF ABBREVIATIONS AND DEFINITION OF TERMS

Abbreviation	Definition
15mer	15 amino acid long synthetic peptide
≥	Equal to or greater than
<	Less than
>	Greater than
β-HCG	beta human chorionic gonadotropin
µg	Microgram
µL	Microlitre
µmol	Micromole
ACTG	AIDS clinical trial group
AE	Adverse Event
AIDS	Acquired Immunodeficiency Syndrome
ALT	Alanine aminotransferase
ANC	Absolute neutrophil count
APC	Antigen presenting cell
ART	Antiretroviral therapy
AST	Aspartate aminotransferase
AV	Atrioventricular
BMD	Bone mineral density
BP	Blood pressure
BSA	Body Surface Area
°C	Degrees Celsius
cc	Cubic centimetre
CD4+	CD4 positive
CD8+	CD8 positive
CE	Conformité Européenne
cm	Centimetre
CPD	Citrate Phosphate Dextrose
CPMP	Committee for Proprietary Medicinal Products
cGMP	Current Good Manufacturing Practice
cm^2	Square centimetre
CRF	Case Report Form
CRO	Contract Research Organisation
CTnI	Cardiac troponin I
CTnT	Cardiac troponin T
D	Day
d	Day (refers to units in laboratory testing)
dL	Decilitre
DMSO	Dimethyl Sulfoxide
DNA	Deoxyribonucelic acid
DSMB	Data Safety Monitoring Board
ECG	Electrocardiogram
ELISA	Enzyme-Linked ImmunoSorbent Assay
ELISpot	Enzyme-Linked Immunosorbent Spot assay
EU	European Union
FDA	Food and Drug Administration
FEV	Forced Expiratory Volume
g	Gram
GCP	Good Clinical Practice
GLP	Good Laboratory Practice
h	Hour (refers to units in laboratory testing)
HAART	Highly Active Antiretroviral Therapy
HBsAg	Hepatitis B surface antigen
HCV	Hepatitis C Virus
HIV	Human Immunodeficiency Virus
HIV-1	Human Immunodeficiency Virus type 1
HLA	Human Leukocyte Antigen
HPF	High power field (microscope)
IB	Opal immunoterhapy Opal-HIV-Gag(c) Clinical Investigator's Brochure
ICH	International Conference on Harmonisation
IEC	Independent Ethics Committee
IMB	Inter-menstrual bleeding
IMP	Investigational medicinal products
IRB	Institutional Review Board
kg	Kilogram
L	Litre
LDL	Low-density lipoprotein
m^2	Meters squared or square metre
MDL	Medicines Development Limited
MedDRA	Medical Dictionary for Regulatory Activities
mEq	milliequivalents
mg	Milligram
MHC	Major Histocompatibility Complex
min	Minute
mL	Millilitre
mm^3	Cubic Millimetre
mmHg	Millimetre of mercury
mmol	Millimole
MNC	Mononuclear cells
MVA	Modified vaccinia Ankara virus
n	Number (typically refers to subjects)
NA	Not applicable
ng	Nanogram
NOAEL	No observable adverse effect level
OECD	Organisation for Economic Co-operation and Development
Opal	Overlapping Peptide-pulsed Autologous Cells
Opal-SIV-All	Overlapping peptides from entire SIV genome
Opal-SIV-Gag	Overlapping peptides from SIV gag protein
Opal-HIV-Gag	Overlapping peptides from HIV gag protein
PBMC	Peripheral Blood Mononuclear Cell
Ph Eur	European Pharmacopoeia
pH	Potential of Hydrogen
PI	Principal Investigator
RBC	Red blood cell
RNA	Ribonucleic acid
SAE	Serious Adverse Event
SAP	Statistical analysis plan
SD	Standard deviation
SHIV	Simian Human Immunodeficiency Virus
SIV	Simian Immunodeficiency Virus
SOP	Standard Operating Procedure
ULN	Upper limit of normal
UNAIDS	Joint United Nations Programme on HIV/AIDS
USA	United States of America
USP	United States Pharmacopeia
v/v	Volume for volume
W	Week
WBC	White blood cell
WHO	World Health Organisation

2 INTRODUCTION

2.1 Background

The December 2008 report of the Joint United Nations Programme on HIV/AIDS (UNAIDS) presented data that an estimated 33 million people (range 30 – 36 million) worldwide were living with HIV at the end of 2007. An estimated 2.5 million of these people became newly infected with HIV and a total of 2.1 million died from HIV/ AIDS in 2007[1]. The availability of a number of antiretroviral medications has enabled the use of combination therapy, known as Highly Active Antiretroviral Therapy (HAART), which has significantly improved prospects for HIV infected individuals and lowered transmission rates. However, the existing therapies for HIV infection have significant shortcomings. The treatment regimens are complex, with limited affordability, and may be associated with treatment-limiting side effects and the emergence of drug resistant viral strains. These factors remain critical barriers to the management of HIV/AIDS, particularly in economically disadvantaged communities. The availability of a simple intermittent immunotherapy, which either delays the introduction of HAART or compliments treatment by HAART, would be a quantum advance in treating HIV.

The induction of HIV-specific T-cell responses is widely seen as critical to maintaining effective control of viral load and subsequent progression to AIDS[2-4], but promising HIV immunotherapeutics that were designed to elicit T-cell responses have generally failed to reduce viral load.

Vaccine candidates will need to augment an immune response that will reduce the loss of CD4+ T-cells and, by reducing viral load and restoring immune responses against HIV, delay disease progression to AIDS. Most therapeutic HIV vaccine studies to date have been hampered either by inefficient induction of relevant immune responses and/or the requirement for extensive and logistically difficult cell manipulations *ex vivo*[5].

An effective therapeutic HIV-1 vaccine will be required to generate specific CD4+ and CD8+ T-cell responses, as early vaccine studies suggest that an antibody response simply places selection pressure on the virus to encourage viral escape and thus renders the specific antibody response ineffective. In contrast, escape from the T-cell mediated immune response may come at the expense of viral fitness[6,7].

Live-attenuated HIV virus vaccines generate potent cellular immune responses, but are likely to be contraindicated in HIV patients. A strategy that has been extensively explored in HIV vaccine research is the delivery of HIV antigens through deoxyribonucleic acid (DNA) or viral vectors. While initially promising, recent studies have suggested that these vaccines fail to be immunogenic in humans or that inappropriate immune responses against the delivery vector are generated. Particle and recombinant whole protein-based vaccines, although well tolerated, favour the generation of antibodies that may encourage the emergence of resistant virus. However, a method that has the potential safety advantage of inert proteins that can also efficiently induce appropriate T-cell responses may be to infuse autologous cells coated with sets of overlapping peptides spanning the target antigen.

Opal is a novel immunotherapy being developed for the treatment of HIV infection. The therapy involves the mixing of Opal with autologous PBMC, enriched white blood cells (WBCs) or whole blood *ex vivo* to generate broad cellular immune responses against the virus. Opal consists of 15mer synthetic peptides that span the HIV structural protein Gag, with each peptide overlapping by 11 amino acids. T-cell responses to the Gag HIV protein have been consistently associated with low viral load[8,9] and there is increasing evidence that viral escape from these T-cell responses occurs at the expense of viral fitness[8]. This suggests that Gag-specific cellular immune responses may limit the emergence of viral escape and thus Gag may be an appropriate target antigen for an HIV therapeutic vaccine. Overlapping 15mer peptides would be expected to cover all CD4+ and CD8+ T-cell epitopes of Gag, regardless of MHC restriction[10], and eliminate the need to include multiple defined peptide

sequences specific to the various human leukocyte antigen (HLA) types. In addition, a substantial degree of cross-clade activity is seen between Gag-specific T-cell responses[11].

2.2 Therapeutic Vaccination for HIV

Unlike non-specific immune boosting treatments, HIV therapeutic vaccines aim to selectively activate the immune system against specific HIV proteins. Although individuals infected with HIV already have circulating viral proteins, it is hypothesised that by presenting these proteins to the immune system in a different way, specific immune responses may be augmented. Therefore, therapeutic vaccination for HIV-1 that leads to the generation of T-cell responses has the potential to at least partially control HIV-1 replication without the need for daily treatment. It is also complimentary to ART, given the T-cell mediated mode of action, and has the potential to preserve future antiretroviral treatment options. Trials of therapeutic vaccines in both animals and humans have generally resulted in increased T-cell responses, as intended. Long term clinical efficacy has not been demonstrated and the specific epitopes within the known HIV proteins that are responsible for viral control are yet to be identified.

The world of HIV immunotherapies is overshadowed by the early failures of Remune (whole, inactivated HIV), Gag virus-like particles and recombinant gp160 protein vaccines. Further studies with canary pox (ALVAC) and fowlpox delivery vectors elicited specific T-cell responses, but overall did not seem to offer any reduction in viral load following HAART interruption when compared to placebo controls[12-14]. The Modified Vaccinia Ankara (MVA) vector is a highly attenuated vaccinia virus vector that takes advantage of the ease with which foreign genes can be inserted into the vaccinia genome, while allaying the accompanying safety concerns of using vaccinia vectors. MVA vaccine candidates have been extensively tested in the clinic but tended to elicit weak primary T-cell responses[15]. These vaccines are currently undergoing evaluation as a boost, following priming with DNA expressing similar HIV antigens, where they still show some promise[16-18]. Various other therapeutics such as a DNA vaccine expressing Gag/Pol/Env protein, a DNA prime expressing Gag/Pol/Env/Rev/Tat followed by MVA boost expressing Gag/Pol/Env and a Vesicular stomatitis virus-HIV-1 vaccine are envisaged to enter Phase I studies during the next year. However, as has recently been seen with the STEP trial conducted by Merck that assessed the safety and immunogenicity of an adenovirus vector with the gag, pol and nef HIV genes[19], the pre-existing immune response in humans to the vector can complicate both the safety and efficacy of vaccines employing a delivery vector.

The administration of a peptide vaccine poses reduced safety concerns. All clinical studies have reported that peptide vaccines were well tolerated with minimal side effects. Peptide vaccines such as Vacc-4x or Vacc-5q have yielded promising immunogenicity results, including a slower decline in CD4+ T-cell numbers once HAART was removed after vaccination compared with unvaccinated controls[20,21]. Autologous dendritic cells pulsed with defined peptides have given varied results, with some studies reporting significant T-cell responses and a tendency towards higher CD4+ T-cell counts, but no effect on viral load[22,23], and some reporting no beneficial effect at all[24].

2.3 Opal-HIV-Gag(c)

Opal-HIV-Gag(c) is a novel peptide-based therapeutic vaccine consisting of 120 small (15mer) peptides, each of which overlaps the proceeding peptide by 11 amino acids. The peptides in Opal-HIV-Gag(c) span the entire region of the HIV-1 protein Gag (a structural protein) of the HIV-1 clade C Durban consensus sequence. The Gag protein is a ubiquitous component of HIV infection.

Opal immunotherapy with Opal-HIV-Gag(c) has been developed using the clade C Durban consensus sequence. The clade C subtype circulates in Southern Africa, India, and China, and is responsible for over 50% of all HIV infections worldwide[25]. It is suspected that Opal-HIV-Gag(c) will have cross clade reactivity, particularly with the dominant clade present in Europe and North America (clade B). Broad cross clade reactivity for CD8+ T-cell medicated responses have been described previously for

the highly conserved regions of the Gag protein[11]. Overlapping peptides spanning proteins are used as they can induce CD4+ and CD8+ T-cells responses without specific MHC matching for subjects to known epitopes.

Opal-HIV-Gag(c) is presented as a sterile white powder which is reconstituted in DMSO (Ph Eur / USP). Opal-HIV-Gag(c) is a lyophilised mixture of equal weight of each of the constituent 120 peptides. Opal-HIV-Gag(c) was manufactured by CS Bio, Inc (Menlo Park, United States of America (USA)) to cGMP standards.

2.3.1 *In vitro* activity

Opal-HIV-Gag(c) is believed to activate T-cells via presentation on antigen presenting cells (APCs) and causes cytokine secretion and proliferation. The experimental systems in which this has been established include fresh and frozen PBMCs (human HIV positive donors).

2.3.2 *In vivo* activity

There have been six non-Good Laboratory Practice (GLP) preclinical studies conducted *in vivo* in pigtail macaques (*Macaca nemestrina*) assessing the efficacy and safety of single and multiple doses of a variety of Opal formulations administered via autologous infusion of PBMC or whole blood. The duration of studies ranged from 11 to 72 weeks and they were conducted at the University of Melbourne by Professor Stephen Kent.

The studies were:

1. A non-GLP macaque study (n=6 *Macaca nemestrina*) demonstrated that the infusion (given 3 times over 20 weeks) of autologous PBMC pulsed with large overlapping sets of Simian immunodeficiency virus (SIV) 15mer peptides (Opal) was well tolerated. Furthermore, both an *in vivo* killing assay and direct immunogenicity assays revealed that infusion of Gag or Pol peptide-pulsed cells boosted the immune response in Simian-Human Immunodeficiency virus (SHIV)-challenged animals previously immunized with SIV Gag/Pol[5].

2. A non-GLP study extended the previous observations to assess the ability of Opal immunotherapy (administered to PBMCs *ex vivo*) to increase T-cell responses during chronic SHIV infection (n=2). In this study, the Opal immunotherapy (administered 4 times over a 55 week period to SHIV-infected macaques) was immunogenic and well tolerated with no clinically relevant signs of toxicity during or after therapy. Enhanced immunogenicity was demonstrated by a sharp increase in SHIV-specific CD4+ and CD8+ T-cell numbers in the SHIV-infected animals following Opal therapy[5].

3. A non-GLP study designed to evaluate the safety and efficacy of Opal immunotherapy was carried out on whole blood rather than isolated PBMC in three (n=3) macaques with controlled long term SHIV infection. Re-infusion of peptide-pulsed cells into the macaques was well tolerated with no clinically relevant safety findings[26].

4. A non-GLP study was performed in 12 animals infected with SHIV to examine the safety and immunogenicity of multiple administrations of whole blood Opal therapy with peptides covering a majority of the viral proteins. This 60 week study confirmed that multiple infusions of Opal were well tolerated with no evidence of toxicity: body weight, food consumption, haematology and clinical chemistry were unaffected throughout the study. The Opal therapy (3 infusions administered monthly from study week 47) also provoked a markedly enhanced T-cell response after each infusion[26].

5. A non-GLP study was conducted to evaluate the efficacy and safety of Opal-SIV-Gag and Opal-SIV-All immunotherapy in 36 *Macaca nemestrina* (n=12 per group) infected with

SIV_{mac251} and receiving ART. Opal-SIV-Gag and Opal-SIV-All were pulsed onto autologous PBMC and both were highly immunogenic, with peak T-cell responses occurring between 12 and 14 weeks after SIV infection (2 weeks post final Opal treatment), but then declining to similar levels as controls by the end of the study at W36. However, when ART was interrupted following Opal, the viral load rebound was less in Opal treated *Macaca nemestrina* than in control animals. In addition, Opal-SIV-Gag induced stronger Gag-specific CD4+ and CD8+ T-cell responses compared to Opal-SIV-All, but the breadth of response was much larger when peptides derived from all SIV proteins were used. This study was subsequently extended to W48 to include 3 further boosts with Opal immunotherapy in order to extend these observations on viral load. Again, Opal-SIV-All immunotherapy elicited a broader T-cell response than Opal-SIV-Gag immunotherapy. There was a mean reduction of HIV RNA of 0.6 log_{10} copies/mL in Opal treated animals compared to controls after 24 weeks and 1.14 log_{10} after 48 weeks. This study was extended to 73 weeks and included 3 further boosts with either Opal-SIV-Gag or Opal-SIV-All. The reduction in viral load of approximately 1 log_{10} copies/mL in treated animals compared to controls was maintained throughout the study. ART was not recommenced during these study extensions. No treatment related adverse effects were observed from Opal throughout the study and extensions[27 28].

6. A non-GLP study was conducted to evaluate the delivery of Opal-SIV-Gag immunotherapy delivered by whole blood or PBMC. Opal treatment, administered as 3 doses 2 weeks apart, was immunogenic in 15 SIV_{mac251} infected animals that had previously been immunised with Opal immunotherapy and no treatment related adverse effects were observed in any macaque treated with Opal immunotherapy. In this study there was no significant difference in the CD4+ and CD8+ T-cell responses induced when the Opal-SIV-Gag peptides were delivered by whole blood or PBMC[28,29].

Opal treatment in SIV infected macaques led to boosting of broad CD4+ and CD8+ T-cell responses to a number of viral antigens, reduced viral load rebound following interruption of ART and delayed disease progression over a 6 month period. Opal was well tolerated with no treatment-related reactions in over 40 animals and 250 administrations of non-cGMP Opal. To date, there have been no treatment-related organ toxicity/deaths, unexpected blood test abnormalities, remarkable findings on physical examinations, body weight loss attributable to procedure or reductions in activity or food intake.

More detailed information is available in the Opal Immunotherapy Opal-HIV-Gag(c) Clinical Investigator's Brochure (IB).

2.3.3 Non-clinical Toxicology

The preclinical toxicological evaluation of Opal-HIV-Gag(c) was conducted in accordance with Organisation for Economic Co-operation and Development (OECD) Principles of Good Laboratory Practice[30] and International Conference on Harmonisation (ICH) S6 Preclinical Safety Evaluation of Biotechnology-Derived Pharmaceuticals[31]. The study was designed in consideration of other regulatory guidelines for testing of vaccines, including the Committee for Proprietary Medicinal Products (CPMP) Note for Guidance on the Preclinical Pharmacological and Toxicological Testing of Vaccines[32] and World Health Organization (WHO) Guidelines on Nonclinical Evaluation of Vaccines[33].

A GLP repeat dose toxicology study was conducted in rhesus macaques (Macaca mulatta). The study assessed the safety of 5 *ex vivo* administrations of Opal-HIV-Gag(c) dissolved in DMSO, each to 9 mL of whole blood. Dose levels were 1.85 milligram (mg) and 18.5 mg per administration, equivalent to the same dose as and 10 times, respectively, the proposed starting dose in this clinical study on a body surface area (BSA) basis.

One animal died a day after being anaesthetised during the DNA vaccine priming phase and prior to Opal therapy initiation. This animal was replaced. Opal therapy was well tolerated at all doses studied

and with a safety profile similar to placebo. There was no evidence of systemic or local toxicity attributable to Opal-HIV-Gag(c), no premature or unscheduled deaths post-therapy initiation, no clinically relevant signs of treatment related local or system reactions to treatment administration, histopathological findings, changes in laboratory parameters, changes in activity or food intake, or clinically significant changes in body weight throughout the study. There was no evidence of immunotoxicity associated with the observed immune response and evaluation of immune system tissues and circulating cells did not reveal any adverse effects.

More detailed information is available in the IB.

2.3.4 Metabolism and pharmacokinetics

It is not known how Opal-HIV-Gag(c) is metabolised as there has not been an evaluation of the metabolism and pharmacokinetics of Opal-HIV-Gag(c). However, based on known principles of metabolism of peptides *in vivo*, it is hypothesised that serum proteases break down the peptides rapidly to constituent amino acids which then are incorporated into amino acid processing pathways. Opal-HIV-Gag(c) comprises amino acids that are unmodified from the natural form.

2.3.5 Method of Administration

In non-human primates, Opal has been administered *ex vivo* by drawing approximately 9 to 18 mL of whole blood, dissolving the vaccine in DMSO, adding vaccine to either whole blood or PBMCs separated with ficoll gradient, incubating for 1 hour then reinfusing.

As a practical alternative to PBMC separation and optimised vaccine presentation during the *ex vivo* incubation, the commercially available (Conformité Européenne (CE)-marked) Biosafe Sepax® S-100 cell separation device will be used to separate the whole blood and enrich the WBC component. The Sepax® S-100 device processes whole blood in a closed, single use disposable kit (Sepax CS490.1 kit) that consists of connecters, tubing, fraction collection bags and a centrifugal chamber. Dissolved Opal-HIV-Gag(c) will be added to the WBC collection bag, incubated for one hour and reinfused into the patient. The key steps in this process were assessed in a pilot study described in Section 2.4.1.

2.4 Clinical trials of Opal-HIV-Gag(c)

Opal-HIV-Gag(c) has not been administered to humans and the risks and benefits to humans are unknown.

2.4.1 Opal-HIV-1002 protocol

This was an open, single centre, non-randomised, non-treatment pilot study with the primary objective of providing study-specific training on the treatment procedures planned for the subsequent clinical trial Opal-HIV-1001. The study also provided the opportunity to assess subject recruitment of the planned population for Opal-HIV-1001, to assess the performance of the chosen blood separation device (the Sepax® S-100 cell separation device) and to collect cells for potential immunogenicity assessments or assay development.

All 6 subjects recruited into the study were male and aged between 38 years and 9 months and 51 years and 2 months, with a median age of 41 years and 9 months. Subjects were of white (n=3), black (n=2) or American Hispanic (n=1) origin. Subjects deemed ineligible for the study all failed the criterion for a cross-reactive clade C response via ELISpot. One subject was rescreened due to low $CD4^+$ T-cell count upon initial examination and was subsequently eligible.

The Sepax® S-100 cell separation device (with disposable kit) was used in this study to reduce the whole blood volume and enrich for the WBC component for each donated sample: 120 mL of venous blood (volume determined by the Genesis™ CM350 blood collection monitor) was collected once from each subject, whilst in the supine position, into the whole blood collection bag (Pall Medical

Cord Blood Collection System 791-01U, USA) containing 25 mL of citrate phosphate dextrose (CPD) anticoagulant. This bag was then sterile welded (Terumo TSCD-II Sterile Tube Welder®) to the single use Sepax CS490.1 (Biosafe, Switzerland) kit. Following the sterile connection of a bag to collect the WBC component (Genesis PediPak®, USA), the blood was separated into WBC, red blood cell (RBC) and plasma components using the Sepax® S-100 cell separation device (Biosafe, Switzerland) and the Generic Volume Reduction Protocol in accordance with the manufacturer's instructions.

An AK-100 sterile sampling line (Biosafe, Switzerland) was connected to the blood collection bag, using the sterile weld device, for the removal of a sample of whole blood for analysis purposes. The rapid seal SE700 hand tubesealer™ (Genesis, USA) was used to seal the tubing and the Turoflex® multi-function hand stripper used to remove the contents of tubing prior to tube sealing and removal of the kit from the Sepax S-100 device.

The blood separation procedure took a median 47 minutes (range 35 to 113 minutes) from the start of blood collection to the completion of separation. The blood collection component had a median duration of 3 minutes (range 2 to 10 minutes), and the separation procedure took a median 19 minutes (range 15 to 71 minutes).

The median recovery of WBCs present in the whole blood into the WBC component was 70% (range 31 to 89%). Subject 010, whose blood separation process had to be repeated, had the lowest yield with 31%. The secondary destination of WBCs was the RBC component (median 17% of WBCs, range 12 to 29%). The haematocrit of the WBC component was mean (± standard deviation [SD]) 63 ± 11%. The mean (± SD) percentage of RBC remaining in the WBC component was 25 ± 5.6%. The total number of lymphocytes and monocytes (reported as mononuclear cells [MNC]) in the WBC components was median 1.4 x 10^8 (range 5.5 x 10^7 to 2.7 x 10^8) cells. A mean (± SD) of 75 ± 7.2% of RBCs was recovered into the RBC component. No cells were present in the plasma components of any subjects.

The study objective of providing an opportunity to train the staff at the trial site on the procedures intended for the Opal-HIV-1001 study was achieved, with minimal difficulties encountered with the procedures. There were no AEs reported. This pilot study has demonstrated that the Sepax S-100 device enriches the WBC populations to recover a median of 70% of WBC that were initially present in whole blood and to reduce the volume of sample from 120 mL to 20 mL. The resultant WBC component yielded an adequate number of the cells for the incubation and reinfusion procedure planned for clinical study Opal-HIV-1001. With the exception of RBC, the blood cell populations recovered in the WBC-enriched component were in similar proportions to those in whole blood.

In all subjects' samples, the number of cells available for exposure to peptides was similar to the applicable range in humans that was extrapolated from pre-clinical studies in non-human primates. In addition, the concentration of peptide per million cells was not significantly lower than was observed in the non-human primate efficacy study.

2.5 Rationale for study

This phase I study is the first step to determine if immunotherapy with Opal-HIV-Gag(c) may have potential utility as a treatment for HIV. Although effective treatments for HIV infection exist, they are limited by the requirement for life-long daily treatment, cost, side effects, and the development of resistance.

An immunotherapy for HIV has a novel mode of action compared to currently licensed treatments for HIV and could be used to prolong the time to initiation of HAART (i.e. preserve therapeutic options), provide an opportunity for a therapeutic break and/or reduce the development of resistance to other therapies. The induction of HIV-specific T-cell responses is widely seen as critical for the effective control of HIV-1. Vaccine candidates will need to augment an immune response that will reduce the

loss of CD4+ T-cells and delay progression of disease by the reduction of viral load, thus restoring immune responses against HIV. It is not known whether this technology will be as immunogenic in humans as it has proven to be in macaques. The absolute levels, breadth and durability of anti-HIV immune responses can only be determined by rigorous clinical evaluation as a prelude to determining whether the vaccine can treat human HIV infection.

2.5.1 Rationale for doses selected

The clinical doses have been selected based on the outcomes of the preclinical non-human primate studies. In the repeat dose toxicology study in the non-human primates, the maximum dose was 5 doses of 18.5 mg Opal-HIV-Gag(c), which is the equivalent of 74 mg/square metre (m^2) of BSA (assumes 0.25 m^2 BSA for a monkey of 3 kilograms [kg]), or 6.2 mg/kg at each of the 5 administrations and this was also determined to be the no observable adverse effect level (NOAEL). The clinical starting dose is 12 mg or 7.4 mg/m^2 (0.2 mg/kg, assuming 1.62 m^2 BSA for a 60 kg human) at each of four administrations and the maximum dose proposed in the clinical trial is 48 mg or 29.6 mg/m^2 (0.8 mg/kg). Hence, the first time in human starting dose for the study will be approximately 10 times lower than the NOAEL in non-human primates (based on the USA Food and Drug Administration [FDA] Guidance for Industry and Reviewers Estimating the Safe Starting Dose in Clinical Trials for Therapeutics in Adult Healthy Volunteers[34]).

The diluent for the peptides is DMSO. A maximum of 800 microlitres (μL) will be added to approximately 20 mL of cells for re-infusion, giving a maximum of 4% DMSO in the re-infused mixture.

In determining the adequacy of the clinical dose for immunogenicity purposes, both the amount of Opal-HIV-Gag(c) and the number of PBMCs exposed have been taken into account. Ethical and practical constraints limit the amount of blood that can be taken from each patient and the volume of blood to be drawn is 120 mL. In order to assess if this would be adequate, we allometrically scaled on BSA to determine the blood volume required to yield a proportionally similar number of PBMCs to that shown to be effective in macaques. The blood volume ranged from 29.5 mL to 120 mL.

From the non-human primate studies (see Section 2.3.2), it was estimated that 9×10^6 to 1.8×10^7 PBMCs were exposed to peptides at each administration for each macaque. Allometrically scaling this to humans (based on BSA) yielded an ideal PBMC count of 5.9×10^7 to 1.2×10^8 for Opal treatment per person. The median total number of MNCs in the WBC component from clinical study Opal-HIV-1002 was 1.4×10^8 (range $5.5 \times 10^7 – 2.7 \times 10^8$). Even the lowest end of the range of MNCs yielded was similar to the calculated ideal PBMCs, although the caveat of relatively low predictive value of allometric scaling in immunology is noted. The number of APCs required to trigger an immune response is not known and is likely to be highly dependent on various other factors, including the ‘danger’ response associated with antigen delivery, the pre-existing cytokine milieu, the presence of $CD4^+$ T-cell help, and whether cross-priming of antigen occurs *in vivo*.

To establish the dose of peptide for efficacy purposes, the results of the preclinical non-human primate studies (see Section 2.3.2) were extrapolated. A range of 0.035 mg to 0.07 mg of peptide per million PBMCs was shown to be efficacious in the primate model. Extrapolating this to humans with the expected PBMC yield from 120 mL of whole blood requires a range of minimum doses of 4.2 to 16.8 mg per administration. The proposed clinical doses to be evaluated span this range with the minimum dose of 12 mg and the maximum dose of 48 mg. This should ensure that adequate peptide is available for PBMC presentation.

It is anticipated that the proposed blood volume and dose of Opal will result in an adequate exposure for humans that is practical, ethically acceptable and likely to be well tolerated.

3 OBJECTIVES

The primary objective of this study is to assess the safety of Opal-HIV-Gag(c) at 3 dose concentrations compared to placebo in HIV-1 infected subjects.

The secondary objectives of this study are:

- to evaluate the immunogenicity of Opal-HIV-Gag(c) in T-cells
- to assess the impact of Opal-HIV-Gag(c) treatment on HIV-1 infection.

4 STUDY DESIGN

4.1 Study Design

This is a phase I, randomised, double-blind, single-centre, sequential dose-escalation, placebo-controlled study of the safety and immunogenicity of Opal-HIV-Gag(c) in HIV-1 positive subjects.

Approximately 27 subjects will be enrolled and followed for 24 weeks on both an inpatient and outpatient basis. Subjects will be treated on 4 occasions over 12 weeks. After completion of treatment at W12, subjects will be followed for 12 weeks for safety and immunogenicity assessments.

Each dose group will be enrolled sequentially, with a sentinel group for each cohort. Satisfactory safety data (from 2 immunisations and 1 week follow up) from each cohort will permit recruitment to the next dose group (see Figure 1). A minimum number of 7 subjects completing the dose level for each dose cohort will be required for a DSMB review for dose escalation. A total of 7 subjects will ensure that at least 4 subjects have received administration of Opal-HIV-Gag(c).

Figure 1: Study design

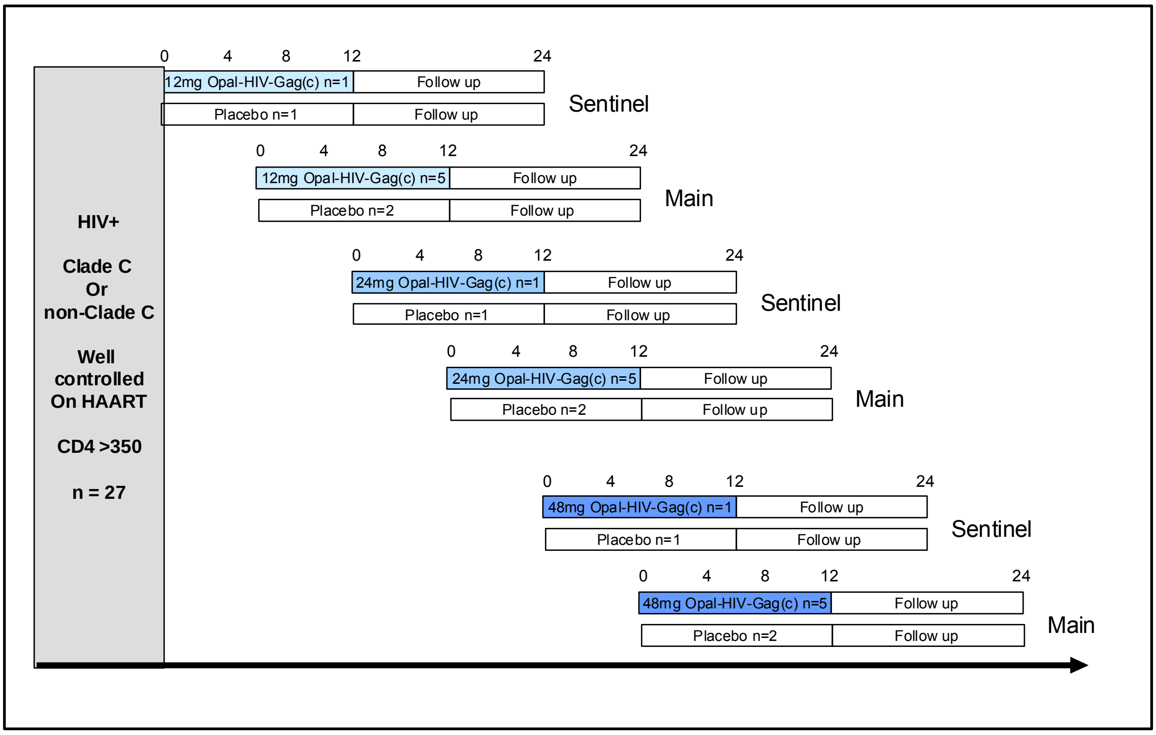

4.2 Dosing Regimens

After screening, subjects will be randomised to the following treatment regimens, starting with the lowest dose (1) and escalating to the highest dose (3):

1. 12 mg Opal-HIV-Gag(c) (n=6) or placebo (n=3)
2. 24 mg Opal-HIV-Gag(c) (n=6) or placebo (n=3)
3. 48 mg Opal-HIV-Gag(c) (n=6) or placebo (n=3)

Within each dose cohort, there will be an initial sentinel cohort of 2 subjects who will be randomised sequentially on a 1:1 basis to receive Opal-HIV-Gag(c) or placebo. The remainder of subjects will be randomised no sooner than 2 days after randomisation of the sentinel subjects.

Subjects will be stratified based on clade of infection. There will be a minimum of 5 clade C subjects per cohort, with the remaining 4 subjects being any clade. The 2 sentinel subjects in each cohort must

be clade C. After the sentinel dosing, the next 3 clade C subjects will be randomised 2:1, and the remaining 4 subjects (of any clade) randomised 3:1.

All subjects will receive a total of 4 immunisations; at D0, 28 (W4), 56 (W8) and 84 (W12). Immunogenicity will be assessed at D0, 70 (W10), 84 (W12), 91 (W13), 98 (W14) and 112 (W16) and all subjects will be followed until W24.

4.3 Study Sites

This will be a single centre study conducted at St Stephen's Centre, Chelsea and Westminster Hospital, London.

4.4 Estimated Duration of the Study

It is anticipated that the total duration of the study will be approximately 16 months including recruitment and 3 months for reporting. The on-study period per subject is up to 30 weeks, including the Screening period.

5 SUBJECT POPULATION

5.1 Selection and Number of Subjects

The nature of the study and the potential risks will be explained to all candidates and written informed consent will be obtained from each subject prior to performing screening procedures. Up to 27 HIV positive subjects who are well controlled on existing ART will be enrolled. There will be no exemptions and subjects must satisfy all eligibility criteria in order to participate. Subjects with laboratory abnormalities that are exclusionary but inconsistent with previous history can be re-screened a single time. Inclusion and exclusion criteria are to be determined at Screening unless otherwise indicated.

5.2 Inclusion Criteria

The criteria for entry into the study are:

1. Provision of written informed consent
2. Documented laboratory diagnosis of HIV-1 infection (either positive ELISA, HIV-1 antibody test confirmed by Western Blot, p24 assay, HIV-RNA, or culture)
3. Documented HIV subtype (clade) of infection
4. 18 - 60 years of age, inclusive
5. Stable ART regimen containing at least 3 active ART agents for at least 2 months prior to Baseline
6. Plasma HIV-RNA <400 copies/mL for 6 months up to and including Screening. Subjects on stable ART with a single value ≥400 copies/mL (i.e. the result is unconfirmed by subsequent testing) within this timeframe may be included at the discretion of the Investigator
7. CD4+ T-cell count ≥350 cells/mm^3 at Screening (with nadir ≥100 cells/mm^3)
8. A positive immunogenic response when stimulated with HIV-1 Gag clade C peptides at Screening
9. Male or female. Women of child-bearing potential must be using two effective methods of contraception and agree to continue to do so from Screening, throughout study medication dosing and for 28 days after the last dose of study medication

5.3 Exclusion Criteria

The criteria for exclusion from the study are:

1. Any serious or active medical or psychiatric illness which, in the opinion of the Investigator, would interfere with treatment, assessment, compliance with the protocol, or subject safety. This would include any active clinically significant renal, cardiac, pulmonary, vascular, or metabolic (thyroid disorders, adrenal disease) illness, or malignancy
2. HBsAg, or HCV antibody and HCV RNA positive at Screening
3. Female subjects who are lactating and those of reproductive potential with a positive urine β-HCG pregnancy test at either Screening or Baseline
4. A new AIDS-defining condition diagnosed within 42 days prior to Baseline visit
5. Known or suspected allergy to DMSO
6. History of allergy or reaction to medications (including peptide or protein containing agents) or history of severe allergy that, in the opinion of the Investigator, might compromise the subject's participation in any way

7. Moderate or severe asthma, defined as at least chronic moderate symptoms which frequently interfere with daily activities and require anti-asthma/anti-inflammatory agents
8. Have received immunomodulating agents (including immunosuppressive agents, interferon or other immune or cytokine-based therapies), immunisation, and/or systemic chemotherapeutic agents within 60 days of Screening or expected to receive these agents during the course of the study
9. Recipient of live attenuated vaccines within 60 days of Screening
10. Recipient of whole killed, toxoid or sub-unit vaccines (e.g. influenza, pneumococcus, tetanus, hepatitis B) within 42 days prior to Baseline
11. Ever received an HIV prophylactic or immunotherapeutic vaccine (does not apply to subjects who have written documentation of receiving placebo or adjuvant only)
12. Recreational and/or therapeutic drug or alcohol use that, in the opinion of the Investigator, might compromise the subject's participation in any way. Subject's urine will be tested for illicit drugs at Screening and entry into the study will be at the discretion of the Investigator (see Section 5.4)
13. Medical or psychiatric condition or occupational responsibilities that may preclude compliance with the protocol
14. Laboratory blood values:
    - Haemoglobin <11.0 g/dL for men and <10.0 g/dL for women
    - Neutrophil count <800/mm^3
    - Platelet count <50,000/mm^3
    - AST or ALT >2.5 times ULN
    - Lipase >2.5 times ULN
    - Amylase >1.5 times ULN (unless serum lipase is ≤1.5 times ULN)
    - Subjects with an estimated creatinine clearance of <80 mL/min
15. Recipients of blood products or immunoglobulins within 6 months prior to Screening or loss of 450 mL or more of blood during the three months prior to Screening
16. Recipients of experimental or investigational agents within 30 days prior to Screening
17. Previous participation in this study

5.4 Other Study Eligibility Criteria Considerations

In order to assess any potential impact on subject eligibility with regard to safety, the Investigator must refer to the relevant document(s) for detailed information regarding warnings, precautions, contraindications, AEs, and other significant data pertaining to the study product(s) being used in this study. Such documents may include, but not be limited to, the IB or other documentation provided by the Sponsor.

All women of reproductive potential (defined as sexually mature women who have had menses within the preceding 24 months and have not undergone hysterectomy, bilateral oophorectomy or tubal ligation) must have a negative urine pregnancy test with a sensitivity of at least 50 International Units/mL performed at Screening and Baseline and prior to receiving each dose of IMP. Women who are not of reproductive potential (who have been postmenopausal for at least 24 consecutive months or have undergone hysterectomy, bilateral oophorectomy or tubal ligation) are eligible without requiring the use of contraception.

Women of reproductive potential must agree not to attempt to become pregnant or undergo *in vitro* fertilisation and, if participating in sexual activity that could lead to pregnancy, must use two reliable

methods of contraception simultaneously while receiving protocol-specified medication and for 28 days after stopping the medication.

A combination of TWO of the following methods must be used:

- Condoms (male or female) with or without a spermicidal agent
- Diaphragm or cervical cap with spermicide
- Intra Uterine Device
- Hormonal-based contraception.

Subjects who are positive for cannabinoids and/or benzodiazepines may be considered for participation in the study at the Investigator's discretion. For other illicit substances detected by urine illicit drug screen at Screening, a single repeat test is permitted. Should the second result be negative, entry to the study will be at the discretion of the Investigator. Counselling will be provided for all subjects with positive urinary illicit drug screening results.

If a subject is screened and eligible for the study but unable to participate in that cohort, they may be rescreened for a subsequent cohort. Rescreening of these subjects must be discussed and agreed with the Medical Monitor.

Subjects who are ineligible for a cohort but likely, in the opinion of the Investigator, to be eligible for a subsequent cohort may be rescreened. Rescreening of these subjects must be discussed and agreed with the Medical Monitor and rescreening should be performed once.

Every effort should be made to keep chronic concurrent medications stable through out the study period.

6 SCHEDULE OF ASSESSMENTS AND PROCEDURES

6.1 Study Schedule of Evaluations

The schedule of assessments is presented in Table 1.

6.2 Study Procedures / Assessment Periods

The study procedures to be conducted for each subject enrolled in the study are listed below. Additional information on the study procedures is provided in Section 6.3. Any deviation from protocol procedures must be recorded in the source documents and the Sponsor must be notified.

All laboratory tests on blood samples will be performed at the selected central laboratory. Refer to the Study Reference Manual provided for information on sample collection and shipment of all required study samples.

Additional visits and/or assessments may be conducted as clinically indicated. For these additional assessments, visit-specific data will not be collected in the Case Report Form (CRF), although all AEs and concurrent medication must be recorded throughout the study period.

6.2.1 Screening visit

Subjects will be screened up to 42 days prior to randomisation (range D-42 to D-14) to determine eligibility for participation in the study. Screening assessments may be conducted on different days if required. The following will be performed and documented during screening:

- obtain written informed consent prior to any study related procedures
- a screening medical history (see Section 6.3.1)
- demographic data (see Section 6.3.1)
- a complete physical examination including: (see Section 6.3.1)
  - vital signs
  - bodyweight
  - height
  - assessment of all appropriate body systems to determine study eligibility
- ECG (see Section 6.3.2)
- concurrent medication assessment (see Section 6.3.1)
- blood samples for (see Section 6.3.3)
  - haematology
  - clinical chemistry
  - HIV serology (if required)
  - HBsAg serology
  - HCV testing
  - HIV viral load assessment
  - CD4+ T-cell counts
  - HIV clade C peptide immunology screening
- urine pregnancy test for women of childbearing potential
- urine sample for illicit drug screening (see Section 6.3.3)

Results of all screening tests must be available and reviewed prior to the subject's Baseline visit. Subjects meeting all the inclusion criteria and none of the exclusion criteria will return to the clinic within 42 days after Screening for Baseline evaluations.

6.2.2 Day 0 - Day 1—Baseline evaluations, randomisation, and study medication administration

Subjects will be asked to fast for at least 4 hours prior to dosing (see section 6.5) and be admitted to the clinic in the morning on D0. Water can be taken *ad libitum*. Prior to administration of study medication, confirmation of eligibility criteria will be performed through the review of the inclusion/exclusion criteria. Subjects meeting all of the inclusion and none of the exclusion criteria will then be randomised according to clade and the following procedures performed and documented as indicated on the CRF.

6.2.2.1 Pre-Dose

- a modified physical examination including: (see Section 6.3.1)
    - vital signs
    - body weight
- urine pregnancy test for women of childbearing potential (see Section 6.3.3)
- urine sample for illicit drug screening (see Section 6.3.3)
- concurrent medication assessment (see Section 6.3.1)

If the subject meets all of the inclusion and none of the exclusion criteria (see Section 5), the Subject may be randomised and the following should be performed:

- ECG (see Section 6.3.2)
- blood samples for: (see Section 6.3.3)
    - haematology
    - clinical chemistry
    - HIV viral load assessment
    - HLA typing
    - CD4+ T-cell counts
    - immunogenicity
    - HIV virus resistance assessment (in accordance with clinical care, see Section 6.3)
- blood/serum samples for banking (see Section 6.3.4)

6.2.2.2 Study medication administration

- study treatment will be administered as described in Section 7.5.3.2
- upon reinfusion of the study medication, subjects will be under constant medical supervision for at least 3 hours, with regular formal medical checks not less than hourly until Hour 8

6.2.2.3 Post-dose

- subjects will remain supine or semi-supine for 4 hours post-administration
- continuous cardiac monitoring via lead 2 for 4 hours
- ECG (see Section 6.3.2) 4 hours post-administration
- a modified physical examination (see Section 6.3.1) at 30 min, 4 and 24 hours post dose
- vital signs (blood pressure (BP), temperature, heart rate and respiratory rate) will be measured at 15, 30, 45, 60, 75, 90 and 105 min, and 2, 3, 4, 6, 8, 12 and 24 hours after dosing
- blood samples for: (see Section 6.3.3)
    - haematology at 4 hours post dose
    - clinical chemistry at 4 hours post dose
- at approximately 4 hours post-treatment, a standard meal will be given. After 8 hours, food will be provided as required.

Subjects remain in the clinic for 24 hours following the administration of study medication. Prior to discharge, subjects will have the following tests performed:

- blood samples for: (see Section 6.3.3)
  - haematology
  - clinical chemistry
- vital signs (see Section 6.3.1)
- AE and concurrent medication assessments

Subjects will be discharged 24 hours post-dose only if there are no ongoing medical issues requiring further follow up.

6.2.3 Day 5, 7, 31, 59 and 87 safety visits conducted by telephone

On D5, 7, 31, 59 and 87, a telephone interview with subjects will be conducted for AE and concurrent medication reporting.

6.2.4 Week 4 in-patient study medication administration visit

Subjects will be asked to fast for at least 4 hours prior to doing (see section 6.5) and be admitted to the clinic in the morning on D28 (W4). This visit should be performed no more than one day before or one day after the scheduled visit date. Water can be taken *ad libitum*. The following tests will be performed and documented as indicated on the CRF.

6.2.4.1 Pre-dose

- AE assessment
- a modified physical examination including: (see Section 6.3.1)
  - vital signs
  - body weight
- urine pregnancy test for women of childbearing potential (see Section 6.3.3)
- urine sample for illicit drug screening (see Section 6.3.3)
- concurrent medication assessment (see Section 6.3.1)
- ECG (see Section 6.3.2)
- blood samples for: (see Section 6.3.3)
  - haematology
  - I-Stat system E3+
  - clinical chemistry
  - HIV viral load assessment
  - CD4+ T-cell counts

6.2.4.2 Study medication administration

- study treatment will be administered as described in Section 7.5.3.2
- upon reinfusion of the study medication, subjects will be under constant medical supervision for at least 3 hours with regular formal medical checks not less than hourly until 8 hours post-treatment.

6.2.4.3 Post-dose

- Subjects will remain supine or semi-supine for 4 hours post-administration
- At approximately 4 hours post-treatment, a standard meal will be given. After 8 hours food will be provided as required.
- Continuous cardiac monitoring via lead 2 for 4 hours
- ECG (see Section 6.3.2) 4 hours post-administration
- a modified physical examination (see Section 6.3.1) at 30 min, 4 and 24 hours post dose
- vital signs (BP, temperature, heart rate and respiratory rate) will be measured at 15, 30, 45, 60, 75, 90 and 105 min, and 2, 3, 4, 6, 8, 12 and 24 hours after dosing

- Blood samples for: (see Section 6.3.3)
  - haematology at 4 hours post dose
  - clinical chemistry at 4 hours post dose

Subjects remain in the clinic for 24 hours following the administration of study medication. Prior to discharge, subjects will have the following tests performed:

- blood samples for: (see Section 6.3.3)
  - haematology
  - clinical chemistry
- AE and concurrent medication assessments
- vital signs (see Section 6.3.1)

Subjects will be discharged 24 hours post-dose only if the subject is clinically well.

6.2.5 Week 8 and 12 in-patient study medication administration visits

Subjects will be asked to fast for at least 4 hours prior to dosing (see section 6.5) and be admitted to the clinic in the morning on D56 (W8) and D84 (W12) respectively. These visits should be performed no more than one day before or one day after the scheduled visit date. Water can be taken *ad libitum*. The following tests will be performed and documented as indicated on the CRF.

6.2.5.1 Pre-dose

- AE assessment
- a modified physical examination including: (see Section 6.3.1)
  - vital signs
  - body weight
- urine pregnancy test for women of childbearing potential (see Section 6.3.3)
- urine sample for illicit drug screening (see Section 6.3.3)
- concurrent medication assessment (see Section 6.3.1)
- ECG (see Section 6.3.2)
- blood samples for: (see Section 6.3.3)
  - haematology
  - I-Stat system E3+
  - clinical chemistry
  - HIV viral load assessment
  - CD4+ T-cell counts
  - immunogenicity at W12
- blood/serum samples for banking (see Section 6.3.4)

6.2.5.2 Study medication administration

- study treatment will be administered as described in Section 7.5.3.2
- upon reinfusion of the study medication, subjects will be under constant medical supervision for at least 3 hours, with regular formal medical checks not less than hourly until 8 hours post-treatment.

6.2.5.3 Post-dose

- subjects will remain supine or semi-supine for 4 hours post-administration
- at approximately 4 hours post-treatment, a standard meal will be given.
- a modified physical examination (see Section 6.3.1) at 30 min, and 4 hours post dose
- vital signs (BP, temperature, heart rate and respiratory rate) will be measured at 15, 30, 45, 60, 75, 90 and 105 min, and 2, 3, 4, 6 and 8 hours after dosing

- continuous cardiac monitoring via lead 2 for 4 hours
- ECG (see Section 6.3.2) 4 hours post-administration
- blood samples for: (see Section 6.3.3)
    - haematology at 4 hours post dose
    - clinical chemistry at 4 hours post dose

Subjects remain in the clinic for 8 hours following the administration of study medication. Prior to discharge, subjects will have AE, concurrent medication and vital signs assessments (see Section 6.3.1). Subjects will be discharged 8 hours post-dose only if there are no ongoing medical issues requiring further follow up.

6.2.6 On-study clinic visits Day 3 and Weeks 2, 6, 10, 13, 14 and 16

Subjects will return to the clinic for assessments on D3, D42 (W6), D70 (W10), D91 (W13), D98 (W14) and D112 (W16). With the exception of D3, for which there is no visit window, these visits should be performed no more than one day before or one day after the scheduled visit date. The following tests will be performed and documented as indicated on the CRF:

- a modified physical examination will be performed on D3 and W2, 6, 10, 13, 14 and 16 including: (see Section 6.3.1)
    - vital signs
    - body weight
- AE assessment to be performed on D3 and W2, 6, 10, 13, 14 and 16
- concurrent medication assessments to be performed on D3 and W2, 6, 10, 13, 14 and 16
- blood samples for: (see Section 6.3.3)
    - haematology to be performed at W2, 6, 10, 13, 14 and 16
    - clinical chemistry to be performed at W2, 6, 10, 13, 14 and 16
    - CD4+ T-cell counts to be performed at W16
    - HIV-1 Viral load assessment to be performed at W16
    - HIV-1 resistance assessment to be performed at W14 in accordance with clinical care (see Section 6.3)
    - immunogenicity to be performed at W10, 13, 14 and 16
- urine pregnancy test for women of childbearing potential to be performed at W16 (see Section 6.3.3)
- blood/serum samples for banking to be performed at W10, 13, 14 and 16 (see Section 6.3.4)

6.2.7 Exit evaluation (Week 24)

This visit should be performed no more than 3 days before or one day after the scheduled visit date. Subjects will return to the clinic. The following tests will be performed and documented as indicated on the CRF:

- a full physical examination including: (see Section 6.3.1)
    - vital signs
    - body weight
- AE assessment
- concurrent medication assessments (see Section 6.3.1)
- blood samples for: (see Section 6.3.3)
    - haematology
    - clinical chemistry
    - CD4+ T-cell counts
    - HIV-1 Viral load assessment (in accordance with clinical care, see Section 6.3)
- urine pregnancy test for women of childbearing potential (see Section 6.3.3)

6.3 Details on Scheduled Assessments

6.3.1 Demographic Data, Medical History, Physical Examination, Vital Signs

Demographic data will include sex, ethnicity and date of birth. The medical history will include any diagnosed and/or recurrent medical conditions, and any medications/medical procedures currently ongoing, including non-prescription medication, will be recorded in the CRF.

A complete physical examination (including head, ears, nose, throat, lungs, lymph nodes, heart, abdomen and skin) will be conducted at Screening to determine study eligibility. A complete physical examination will also be conducted at the exit visit (W24) or at the early termination visit if applicable. At other scheduled visits, a full physical examination is recommended if, in the opinion of the Investigator, any clinical changes are suggested by the subject's interim clinical history.

A modified physical examination will be performed at all other scheduled clinic visits. This modified physical examination will be performed to document physical findings of systems appropriate to assessing the clinical status of the disease process.

Vital signs will be measured at every scheduled clinic visit and at the early termination visit if applicable. Vital signs will be measured by:

- body temperature (degrees Celsius [°C])
- respiratory rate (breaths/min)
- pulse rate (beats/min),
- BP (mm mercury [mmHg])

BP and pulse are to be recorded after the subject has been semi-supine for at least 10 minutes. These parameters should be measured when the subject is rested sufficiently. Refer to the Study Reference Manual for more details of the methods to be used.

Vital signs will also be measured at other times if deemed clinically appropriate.

Body weight (kg, without shoes) will be measured at Screening, Baseline, W4, 8, 12, 16 and 24 and at the early termination visit if applicable.

Height (centimetres [cm], without shoes) will be measured and documented at the Screening visit.

6.3.2 Electrocardiograms

The 12-lead electrocardiogram (ECG) recordings in this clinical trial will be performed in a standardised manner as outlined in the Study Reference Manual. Repeat measurements will be performed if there are any clinical abnormalities observed or artefacts are present. All 12-lead ECG recordings will be reviewed by the Investigator or nominee in real time.

The 12-lead ECG recordings will be performed once the subject has been semi-supine for at least 10 minutes and will be measured in triplicate over approximately 3 minutes. The mean of the triplicate readings will be used to determine eligibility for the study. ECG data must be reviewed by the Investigator or nominee prior to dosing to ensure that each subject has no clinically relevant findings on ECG prior to dosing.

Continuous cardiac monitoring of each subject will be performed on dosing days until 4 hours post-dose and an ECG recorded at approximately 30 min pre-dose and 4 hours post-dose. A 12-lead ECG will be performed for any out of range or arrhythmia alarms deemed clinically significant by the Investigator. Any clinically significant out of range or arrhythmia alarms will be printed out and recorded in the subject's CRF and reported as AEs.

6.3.3 Blood and Urine samples for laboratory tests

Blood will be collected at all scheduled clinic visits (and at the early termination visit if applicable) for the tests specified in Table 2 at the times specified in Section 6.2. The times the samples are actually taken will be recorded. All times must be recorded in the 24 hour format.

Table 2: Laboratory Parameters

Test	Parameters
Haematology:	Haemoglobin, haematocrit, RBC and RBC morphology, WBC and differential WBC count, platelet count, mean corpuscular volume, mean corpuscular haemoglobin concentration, prothrombin time.
I-Stat system:	Sodium, potassium, haemoglobin and haematocrit pre-dose on W4, 8 and 12.
Chemistry:	Sodium, potassium, chloride, bicarbonate, blood urea nitrogen, creatinine, total protein, albumin, serum amylase, serum lipase, phosphate, AST, ALT, gamma glutamyl transferase, alkaline phosphatase, total bilirubin, glucose, creatine phosphokinase, calcium and uric acid levels. Ferritin, Vitamin B12 and serum folate will be tested at Screening.
Serology:	HIV (unless previously performed), HCV antibody and HBsAg (at screening only).
HCV RNA:	HCV RNA to be tested if HCV antibody is positive (at Screening only)
HIV viral load:	Serum samples will be collected for HIV RNA viral load testing at Screening, Baseline, W4, 8, 12, 16, and 24/Early termination
HIV Resistance assessment:	Blood will be collected for a HIV resistance assessment if the subject has measurable viral load in accordance with standard clinical care.
CD4+ T-cell counts:	Blood will be collected for CD4+ T-cell counts at Screening, Baseline, W4, 8, 12, 16, and 24/Early termination.
Immunogenicity:	Blood will be collected at Baseline, W10, 12, 13, 14 and 16. Immunogenicity will be assessed by ELISpot and other immunological assays.
Plasma Banking:	A portion of the sample (at least 5 aliquots of 1 mL) at Baseline, W10, 12, 13, 14 and 16 will be banked for exploratory analyses. Plasma will be collected from blood samples for immunogenicity.

Urine samples will be collected from women of child bearing potential at Screening, Baseline, W4, 8, 12, 16, and 24/Early termination for pregnancy testing.

Urine samples will be collected from all subjects at Screening, Baseline, W4, 8 and 12 for illicit drug testing.

6.3.4 Handling and processing of biological specimens

Blood and urine specimens collected during the trial will contain HIV-1 and possibly other pathogens. All personnel involved in collecting and handling biological specimens should follow appropriate precautionary procedures for handling biohazardous materials as currently recommended by the national regulatory authority. The processing of all biological specimens will be in accordance with relevant written Standard Operating Procedures (SOPs).

Further details of the handling of blood samples can be found in the Study Reference Manual.

6.3.5 Time of blood incubation and infusion activities

The time taken for the steps of blood incubation and/or infusion should be recorded.

6.4 Visit windows

Subjects should attend clinic visits on the days specified. Visits on W2 (D14), W4 (D28), W8 (D56), W12 (D84), W14 (D96) and W16 (D112) should occur within -1 day to +1 day of the scheduled day. The W24 visit should occur within -3 days to +1 day of the scheduled day. There will be no visit window for safety visits conducted via telephone on D5, D7, D31, D59 and D87.

6.5 Fasting

Subjects will be asked to fast on D0, W 4, W8 and W12 visits. This will usually be from midnight the preceding evening. However, if dosing is not scheduled to take place before midday on D0, W4, W8 or W12, a light breakfast is allowed. Subjects must fast at least 4 hours before the pre-dosing blood draw.

7 STUDY PRODUCTS

7.1 Randomisation process

Potential subjects who provide written informed consent will be sequentially assigned a Screening Number prefixed by "S" (S001-) and subjects who meet the inclusion/exclusion criteria and are to be randomised will be assigned a Subject Number 001to 027 sequentially. A randomisation scheme will be prepared in advance by the study statistician using a computer generated system according to relevant SOPs. The scheme will have the property that within each dose cohort, the initial sentinel cohort of 2 clade C subjects will be randomised 1:1 to receive active treatment or placebo, the next 3 clade C subjects will be randomised in a 2 (active):1 (placebo) ratio, and the remaining 4 subjects (who may be of any clade) will be randomised in a 3:1 ratio.

7.2 Blinding

The study will be conducted double-blind. Study subjects, investigators and monitors will be blind to the administration of active or placebo to subjects. The study pharmacist or delegate will not be blinded during the study and will have access to the randomisation code. The treatment procedure will require an unblinded pharmacist for the preparation and delivery of the study materials. In order to maintain the blind, the pharmacist will not divulge the treatment allocations to any other study or sponsor staff. Treatment will be labelled and presented in a manner that does not break the blind.

7.3 Treatment Allocation

The pharmacist will prepare the treatment corresponding to the Subject Number in the randomisation scheme, stratifying according to HIV clade. The first two subjects must be clade C. A period of at least 2 days must elapse between the start of treatment of sentinel subjects and the start of treatment for remaining subjects in the same cohort. The pharmacist will record the name of the subject and the randomisation date against the Subject Number on the randomisation scheme. This entry must be signed by the pharmacist.

7.4 Method of Unblinding

7.4.1 Medical emergency

As the study is blinded, the clinical site, the laboratories, the Contract Research Organisation (CRO) or MDL will not have access to the open randomisation scheme. However, pharmacy will hold the open randomisation scheme and sealed code break envelopes will be held by the clinical site, medical monitor and MDL. In the event of a medical emergency requiring breaking of the blind, the Investigator must contact MDL or the Medical Monitor prior to doing so. The breaking of the blind will only be sanctioned where knowledge of the study medication treatment will affect subject management.

If the code is broken, the envelope must be signed and dated by the individual who broke the code, and the Code Break Log completed (and information entered into the subject's source documents and CRF), explaining the reason and date that it was opened, identity of the person who authorised the code break, and documenting the identity of the study treatment allocated to the subject. The Code Break Log must be signed and dated and countersigned by the Investigator.

7.4.2 End of study

The randomisation code will be broken by the study statistician once data entry has been completed, the database locked, the per-protocol population for analysis established and the Statistical Analysis Plan (SAP) finalised and approved. MDL will provide written permission to the study statistician prior to the breaking of the randomisation code.

7.5 Investigational medicinal products

7.5.1 Formulation

The active IMP (Opal-HIV-Gag(c)) contains 120 peptides (15 amino acids in length overlapping the preceding peptide by 11 amino acids) spanning the HIV-1 clade C Gag protein. The peptides were synthesised and lyophilised by CS Bio Inc. (USA) according to US FDA standards of cGMP as defined by United States 21 Code of Federal Regulations.

To formulate the Opal-HIV-Gag(c), individual peptides were reconstituted and pooled in equal amounts by weight. The pooled peptides were filled into vials (at 60 mg/vial), lyophilised to produce a dry powder and sterilised by terminal gamma irradiation. The final product release specifications are listed in the IB. The material for clinical administration will be released by a qualified person according to the regulations for release of IMP manufactured outside the EU for use in European trials.

The comparator placebo material will consist of the diluent only delivered using the same method of administration. The diluent for the active and the placebo is DMSO USP/ Ph Eur. To minimise the risk of toxicity associated with DMSO, the overall concentration of the DMSO will be 4% volume per volume (v/v). Side effects are reported more commonly in stem cell transplantation where 10% DMSO v/v is used in standard procedures and reinfused[35]. The re-infusion will also be a slow push or controlled infusion by infusion pump (not more than 5 mL/min[36]), to reduce the risk of vasospasm or toxicity. Subjects will be monitored and history of allergy to DMSO will be an exclusion criterion to participation in the study.

7.5.2 Supply, packaging and labelling, storage and handling

The Opal-HIV-Gag(c) must be stored in a secure area with access limited to the unblinded pharmacist and authorised staff.

The vials and outer packaging will be stored at -20°C in a controlled and monitored freezer (range -25°C to -15°C).

At a minimum, the immediate packaging will include the following information:

- sponsor name
- protocol number Opal-HIV-1001
- product name/drug code
- dose/product strength
- route of administration
- lot number
- directions for use
- expiry date or retest date (on outer packaging)
- ‘For Clinical Trial Use Only’

Any specific cautionary statements will be included according to local law.

Opal-HIV-Gag(c) will be administered by mixing with blood that has been removed and processed via a Sepax S-100 device (CE marked) and single use kit. Opal-HIV-Gag(c) will be provided to Pharmacy and single use kits will be provided to the site. The single use kits must be stored protected from light and in accordance with the manufacturer’s specifications. Kits must not be used if they have exceeded their expiry date or if the sterility label is compromised on the packaging (refer to Study Reference Manual).

DMSO Ph Eur/USP is provided as commercially available supply and will be labelled and stored in accordance with the manufacturer’s specifications.

7.5.3 Dosage and administration of investigational medicinal product

Only subjects enrolled in the study may receive Opal-HIV-Gag(c) or matching placebo. Subjects will receive Opal-HIV-Gag(c) or matching placebo at St Stephen's Centre.

7.5.3.1 Dose of investigational medicinal product

The dosage calculations at each dose level for each visit (W0, 4, 8 and 12) are provided in Table 3 below. Further details of dose and reconstitution are listed in 7.5.3.2 and the Study Reference Manual.

Table 3: Dose of investigational medicinal product in 20 mL WBC-enriched blood component

Dose cohort	Dose per peptide	Total amount of Opal-HIV-Gag(c)	Volume DMSO as diluent added to reconstitute	Final volume of IMP drawn into syringe and administered#
1*	5 microgram (µg)/peptide/mL	12 mg	4 mL	800 µL
2*	10 µg/peptide/mL	24 mg	2 mL	800 µL
3*	20 µg/peptide/mL	48 mg	1 mL	800 µL

* Refers to active IMP administration. Placebo recipients for all dose cohorts will be administered 800 µL of DMSO diluent.
# The administered volume of IMP for all Opal-HIV-Gag(c) and placebo is 800 µL, which will constitute a total of 4% v/v of DMSO added to the 20 mL WBC enriched cells.

7.5.3.2 Administration of investigational medicinal product

The following activities are required for preparation and administration of the IMP, in accordance with the randomisation scheme (please refer to the Study Reference Manual for further details):

Pharmacy Procedure

Prepare the IMP for one subject at a time. The preparation of the IMP must be conducted by pharmacy personnel experienced in the use of aseptic techniques within an appropriate area for preparation of sterile products (such as a pharmacy isolator). The maximum time from drawing the Opal-HIV-Gag(c) dissolved in DMSO Ph Eur/USP or DMSO Ph Eur/USP placebo into the dosing syringe to addition of the IMP to the WBC component should not exceed 3 hours. The IMP in the dosing syringe is to be kept at room temperature (within an insulated "cool box") prior to addition to the WBC component.

<u>Preparation of placebo dose:</u>

1. Within the area appropriate for preparation of sterile products and using aseptic technique:
    a. Swab the DMSO Ph Eur/USP lid with alcohol
    b. Withdraw 800 µL from the vial of DMSO Ph Eur/USP with a sterile 1 mL polypropylene syringe and needle
    c. Remove and discard the needle and immediately attach a sterile capped needle to the syringe
    d. On the syringe label, complete the subject number, and attach label to the syringe
2. Place the syringe in an insulated "cool box " at room temperature for transfer to the clinical site
3. Record the lot number of the DMSO Ph Eur/USP and discard any remaining DMSO Ph Eur/USP
4. Contact the clinical site personnel for collection of IMP.

<u>Preparation of Opal-HIV-Gag(c) dose:</u>

1. Remove a vial of Opal-HIV-Gag(c) from storage at -20°C and thaw at room temperature for 30 minutes
2. Within the area appropriate for preparation of sterile products and using aseptic technique:
    a. Swab the DMSO Ph Eur/USP lid with alcohol

b. According to the table below, withdraw the required volume of DMSO Ph Eur/USP from the vial using a sterile 5mL polypropylene syringe with a needle

Dose cohort	Volume of DMSO Ph Eur/USP for reconstitution
1	4 mL
2	2 mL
3	1 mL

i. Swab the Opal-HIV-Gag(c) vial lid with alcohol
ii. Inject the required volume of DMSO Ph Eur/USP into the vial of Opal-HIV-Gag(c), discard needle and syringe and swirl the vial to reconstitute material, checking it is dissolved fully
iii. Using a sterile 1 mL polypropylene syringe with a needle, draw 800 µL of Opal-HIV-Gag(c) in DMSO Ph Eur/USP solution into the syringe
iv. Remove and discard the needle and immediately attach a sterile capped needle
v. On the syringe label, complete the subject number according to the randomisation code, and attach label to the syringe.

c. Place the syringe with capped needle in a insulated "cool box" at room temperature for transfer to the clinical site

3. Record the lot numbers of the Opal-HIV-Gag(c) and DMSO Ph Eur/USP and discard any remaining DMSO Ph Eur/USP
4. Contact the clinical site personnel for collection of IMP.

Clinic Procedure

Standard clinical procedures are to be used throughout the administration of the IMP unless otherwise stated. The IMP must not be directly injected into the subject. The time between the addition of the IMP to the WBC collection bag (Genesis PediPak®, USA) and initiating re-infusion must not exceed 2 hours. The time for infusion must not be less than 5 minutes and should not exceed 1 hour.

IMP storage at the clinic:

1. The IMP should be retained at room temperature in the insulated "cool box" upon arrival at the clinic until required

Blood collection for IMP administration:

2. A total of 120 mL of whole blood is collected into the whole blood collection bag (Pall Medical Cord Blood Collection System 791-01U, USA), with the volume determined using a CM350 blood collection monitor (Genesis®, USA)

Blood separation kit modification and blood separation:

3. Using the sterile connection device (Terumo TSCD-II Sterile Tube Welder®), attach a sterile single use blood separation kit (Biosafe, Switzerland) to a sterile WBC collection bag (Genesis PediPak®, USA) and the whole blood collection bag
   Note: The WBC collection bag collects the separated WBC component
4. Separate the blood into three components (plasma, RBC and WBC) using the Sepax® S-100 cell separation device and the "Generic Volume Reduction Protocol"
5. Remove the bag containing the WBC component (there should be approximately 20 mL in the Genesis PediPak®) from the blood separation kit) by heat sealing using a rapid seal SE700 hand tubesealer™ (Genesis, USA) at 3 points of the tubing and cutting between the seals
6. Discard the kit and remaining blood components (plasma and RBC) appropriately
7. Use the sterile connection device to attach a sterile AK-100 sample line to the WBC collection bag and collect 0.5 mL of the WBC component. Remove the sample line by heat sealing using a rapid seal SE700 hand tubesealer™ (Genesis, USA) at 3 points of the tubing and cutting between the seals.

Incubation of IMP and re-infusion:

8. Remove the syringe and needle containing the IMP from the insulated "cool box"
9. Swab the non-latex injection site with alcohol
10. Inject the IMP into the WBC collection bag through the non-latex injection site on the bag
11. Mix the IMP and WBC component by gentle inversion of the bag 5 times and place into the incubating rocking platform shaker (VWR Signature™ USA) for 1 hour at 37°C
12. Attach an infusion giving set for blood into the sterile access port on the WBC collection bag
13. Using an infusion pump with the giving set, administer the infusion intravenously over a period of time not less than 5 minutes
14. Flush with approximately 50 mL of sterile saline
15. Remove and discard the infusion giving set.

7.5.4 Dispensing and accountability

The pharmacist or delegate will be responsible for maintaining accurate records for all study medications dispensed and returned. The inventory must be available for inspection by the study monitor. Study product supplies, including partially used or empty vials, must be accounted for by the study monitor and returned to the drug repository for destruction at the end of the study.

When requested in writing by the sponsor, unused study medication supplies may be destroyed by the pharmacy according to their SOPs provided such disposition can be performed safely. Records shall be maintained by the Investigator for any alternate disposition of the study medication. These records must show the identification and quantity of each unit disposed of, the method of destruction (taking into account the requirements of local law) and the person who disposed of the test substance. Such records shall be submitted to the sponsor.

8 CONCOMITANT MEDICATIONS AND TREATMENTS

At each study visit or contact, the Investigator should question the subject or their legal representative about any medication taken. Concomitant therapies should be brought to the attention of the Investigator. Any such medications will be recorded on the CRF. Any changes in doses or introduction of new medications during the course of the study will also be recorded.

8.1 Special Dietary Requirements

There are no special dietary requirements.

8.2 Concomitant Medications / Treatments Not Permitted

8.2.1 Prior to study entry

Refer to Exclusion Criteria in Section 5.3.

8.2.2 During the study dosing period

Throughout the study medication dosing period, subjects may not receive any of the following concomitant medications:

- immunomodulators, immunoglobulins and other blood products, cytotoxic chemotherapy or the any use of experimental agents are not permitted
- live attenuated vaccines may not be administered within 60 days of entering and throughout the duration of the study
- whole killed/toxoid (influenza, pneumococcal, tetanus) or sub-unit vaccines (hepatitis B) should not be given during the vaccination period of the study or in the 30 days following the last vaccination unless medically indicated.

All concurrent medications, including vitamin supplements and herbal remedies, must be recorded in the appropriate section of the CRF.

Every effort should be made to keep chronic concurrent medications stable throughout the study period.

9 ADVERSE EVENTS AND TOXICITY MANAGEMENT

9.1 Safety Parameters

Safety parameters will include AEs (see Section 9.2), vital signs (see Section 6.3.1), clinical laboratory tests (see Section 6.3.3), physical examinations (see Section 6.3.1) and ECG's (see Section 6.3.2).

9.2 Adverse Events

An AE is any untoward medical occurrence in a subject or clinical investigation subject administered an IMP (i.e. whether it is the experimental product or the placebo) and which does not necessarily have a causal relationship with the IMP. An AE can therefore be any unfavourable and unintended sign, symptom, or disease temporally associated with the use of a medicinal product, whether or not considered related to the medicinal product. Pre-existing events, which increase in frequency or severity or change in nature during or as a consequence of use of a drug in human clinical trials, will also be considered as AEs. AEs may also include pre- or post-treatment complications that occur as a result of protocol-mandated procedures (e.g. invasive procedures such as biopsies).

Any medical condition or clinically significant laboratory abnormality with an onset date before the first date of study product administration is considered to be pre-existing, and should be documented in the CRF.

Any AE (i.e. a new event or an exacerbation of a pre-existing condition) with an onset date after study product administration up to the last day on study (including the follow-up, off study medication period of the study), should be recorded as an AE on the appropriate CRF page(s).

An AE **does not** include:

- medical or surgical procedures (e.g. surgery, endoscopy, tooth extraction, transfusion); the condition that leads to the procedure is an AE
- pre-existing diseases or conditions present or detected prior to start of study product administration that do not worsen
- situations where an untoward medical occurrence has not occurred (eg. hospitalisation for elective surgery, social and/or convenience admissions)
- overdose of either study product or concomitant medication without any signs or symptoms unless the subject is hospitalised for observation.

9.2.1 Assessment of Adverse Events

All AEs will be assessed by the Investigator and recorded on the appropriate CRF page, including the date of onset and resolution, severity, relationship to study product, outcome, action taken with study medication, and seriousness.

Severity should be recorded and graded according to the AIDS Clinical Trial Group (ACTG) grading scale (see Appendix A) or, if there are no specific toxicity grades for the parameter, the grading in Table 4 used.

Table 4: Grading Scale where AIDS Clinical Trial Group Toxicity Grades Do Not Apply

Grade	Severity	Definition
Grade 1	Mild	Aware of sign or symptom, but easily tolerated
Grade 2	Moderate	Discomfort enough to cause interference with usual activities
Grade 3	Severe	Incapacitating with inability to work or perform usual activities
Grade 4	Life-threatening	Participant is at immediate risk of death

The relationship to study product therapy should be assessed using the following definitions:

Unrelated: AE is clearly due to extraneous causes (e.g. underlying disease, environment)

Unlikely: The temporal association between the AE and IMP is such that IMP is not likely to have any reasonable association with the AE

Possible: The AE could have been produced by the subject's clinical state or IMP

Probable: The AE follows a reasonable temporal sequence from the time of IMP administration, abates upon discontinuation of the IMP and cannot be reasonably explained by the known characteristics of the subjects clinical state

Definite: The AE follows a reasonable temporal sequence from the time of IMP administration, abates upon discontinuation of the IMP and/or reappears when IMP is re-introduced

These criteria in addition to good clinical judgment should be used as a guide for determining the causal assessment. If it is felt that the event is not related to IMP, then an alternative explanation should be provided.

9.3 Serious Adverse Events

A **serious adverse event** (SAE) is defined as follows:

Any adverse experience that results in any of the following outcomes:

- death
- life-threatening situation (subject is at immediate risk of death)
- in-patient hospitalisation or prolongation of existing hospitalisation (excluding those for study therapy or placement of an indwelling catheter, unless associated with other serious events)
- persistent or significant disability/incapacity
- congenital anomaly/birth defect in the offspring of a subject who received study product
- other: Important medical events that may not result in death, be immediately life-threatening, or require hospitalisation, may be considered a SAE when, based upon appropriate medical judgment, they may jeopardise the subject and may require medical or surgical intervention to prevent one of the outcomes listed in this definition. Examples of such events are:
    - intensive treatment in an emergency room or at home for allergic bronchospasm
    - blood dyscrasias or convulsions that do not result in hospitalisation
    - development of drug dependency or drug abuse.

9.3.1 Clarification of Serious Adverse Events

Death is an outcome of an AE, and not an AE in itself. In reports of death due to "Disease Progression", where no other information is provided, the death will be assumed to have resulted from progression of the disease being treated with the study product(s).

All deaths in the following periods, regardless of cause, must be reported to MDL:

- deaths of subjects on study
- deaths occurring within 30 days of last study product dose or within 30 days of last study evaluation, whichever is longer.

"Life-threatening" means that the subject was at immediate risk of death from the event as it occurred. This does not include an event that might have led to death, if it had occurred with greater severity.

Complications that occur during hospitalisations are AEs. If a complication prolongs hospitalisation, it is a SAE.

"In-patient hospitalisation" means the subject has been formally admitted to a hospital for medical reasons, for any length of time. This may or may not be overnight. It does not include presentation and care within an emergency department.

The Investigator should attempt to establish a diagnosis of the event based on signs, symptoms and/or other clinical information. In such cases, the diagnosis and not the individual signs/symptoms should be documented as the AE and/or SAE.

9.3.2 Serious adverse event reporting requirements

9.3.2.1 All serious adverse events

MDL has requirements for expedited reporting of SAE's meeting specific requirements to worldwide regulatory authorities; therefore, all appropriate parties must be notified immediately regarding any SAE that occurs after the first dose of study product has been administered. The procedures for reporting all SAEs, regardless of causal relationship, are as follows:

- complete the "Serious Adverse Event Report" CRF page
  - fax the SAE report to the SAE hotline within 24 hours of the Investigator's knowledge of the event at +61 3 9629 8718
  - for fatal or life-threatening events, also fax copies of hospital case reports, autopsy reports, and other documents when requested and applicable.

The Sponsor may request additional information from the Investigator to ensure the timely completion of accurate safety reports.

The Investigator must take all therapeutic measures necessary for resolution of the SAE. Any medications necessary for treatment of the SAE must be recorded in the concomitant medication section of the subject's CRF.

9.3.2.2 Investigator reporting requirements for serious adverse events

An SAE may qualify for reporting to regulatory authorities if the SAE is considered to have a possible causal relationship to the study product and is unexpected/unlisted based upon the current IB. In this case, all Investigators will receive a formal notification describing the SAE.

Where this is required by local regulatory authorities, and in accordance with the local institutional policy, the Investigator should notify (in writing) the Independent Ethics Committee (IEC) of SAEs as soon as is practical.

9.4 Follow up of Serious and Non-serious Adverse Events

Follow-up of serious and non-serious AEs will continue through the last day on study (including the follow-up, off study medication period of the study), until the Investigator and/or the Sponsor determine that the subject's condition is chronic or stable, or up to 30 days after the last dose of IMP, whichever is longer. The Sponsor may request that certain AEs be followed until resolution.

9.5 Clinical Laboratory Abnormalities and Other Abnormal Assessments as Adverse Events or Serious Adverse Events

All laboratory values must be reviewed in real time by the Investigator. Given that all laboratory data are collected and statistically analysed according to their respective toxicity gradings, laboratory abnormalities that occur without related clinical symptoms and signs should generally not be recorded as adverse events. Where possible, the overall diagnosis rather than the laboratory abnormality should be recorded on the AE page of the CRF. This will avoid duplication of laboratory abnormalities in both the AE and laboratory reports. Abnormal laboratory results that are of clinical significance may be reported as AEs and should be reviewed with the Medical Monitor.

Any laboratory test result that meets the criteria for a SAE (refer to Section 9.3) should be recorded as an AE, the AE page of the CRF completed and a SAE form also completed in order for the Sponsor to collect additional information about that abnormality, including information regarding relationship to study product or other causes, any action taken and resolution.

9.6 Guidance for toxicity management

AEs and abnormal laboratory values will be graded according to the Toxicity Grading Scale (Appendix A). IMP will be interrupted or discontinued due to toxicity according to the toxicity grading scale.

All abnormal values should be confirmed by repeat testing within 3 calendar days of receipt of results prior to dose adjustment or interruption, unless such a delay is not consistent with good medical practice. For the purpose of monitoring toxicities, the baseline value is defined as the last value prior to the administration of the first dose of study medication. The value must be obtained from the central laboratory. All assessments of toxicity will be based on changes from this value, regardless of later interruptions to dose.

The following table (Table 5) outlines the procedure for temporary or permanent IMP discontinuation due to an AE or SAE. All subjects who experience a study product related AE should be followed until resolution of the AE, even if the subject has interrupted or discontinued study product.

Table 5: Toxicity management.

Clinical or laboratory* abnormality	Action Taken	
	**Continue dosing at Investigator discretion**	**Permanently discontinue dosing**
Grade 1	Regardless of causality	N/A
Grade 2	Regardless of causality	N/A
Grade 3<br>Lab events of clinical significance*<br>Clinical Events	 Unrelated to treatment<br>Unrelated to treatment	 Unknown or at least possibly related<br>Unknown or at least possibly related#
Grade 4<br>Lab events of clinical significance*<br>Clinical Events	 Unrelated to treatment<br>Unrelated to treatment	 Unknown or at least possibly related<br>Unknown or at least possibly related

* All grade 3 or 4 laboratory abnormalities must be confirmed by repeat testing as soon as possible, preferably within 24 hours of receipt of results prior to dose interruption of discontinuation, unless such a delay is not consistent with good medical practice.

#The Investigator may continue dosing at their discretion for transient and self limiting events that may be attributed to IMP but are expected for a vaccine (such as fever): these grade 3 event(s) must be followed to resolution (≤grade 2) before the subject is discharged from the clinic.

9.7 Warnings and Precautions

The risks of treatment with Opal-HIV-Gag(c) not been evaluated and the risks are unknown. For information regarding precautions and AEs with the IMP, the Investigator is referred to the IB for Opal-HIV-Gag(c).

9.8 Risks for Women of Childbearing Potential or during Pregnancy

The risks of treatment with Opal-HIV-Gag(c) during pregnancy have not been evaluated. Pre-menopausal women of childbearing potential will follow a medically prescribed birth control regimen using two methods of contraception or agree to abstain from heterosexual intercourse while participating in the study and for 30 days following the last dose of study product.

9.9 Procedures to be followed in the Event of Pregnancy

The subject must be instructed to inform the Investigator IMMEDIATELY if she becomes pregnant during the study, and seek advice regarding discontinuation of study medication. Whenever possible, treatment should be discontinued. The Investigator should report all pregnancies to the Sponsor within 24 hours of becoming aware of the pregnancy. Pregnancies should be reported using the form in the CRF for reporting the occurrence and outcome of pregnancies in subjects enrolled in the study.

Prior to discontinuing therapy, the Investigator should counsel the subject and discuss the risks of continuing study product dosing and the possible effects on the foetus.

Monitoring of the subject should continue until conclusion of the pregnancy. The outcome of the pregnancy should be reported to the Sponsor regardless of whether the study is still in progress or not.

10 DATA SAFETY MONITORING BOARD REVIEW

The Sponsor has convened a DSMB that is charged with the review of this study. The DSMB review will occur as soon as practical after the W4 dose administration of IMP is complete for each cohort and will include all available safety data.

Each DSMB meeting will have an open session and an optional closed session. Open session review materials will be blinded and include aggregate data on study accrual, baseline characteristics and general study progress, and will be made available to the DSMB, Sponsor representatives, and invited guests (e.g. participating Investigators) to facilitate a general discussion of study progress. Attendance at the closed session will be restricted to DSMB members and those individuals who were directly involved in the preparation of the review materials. The DSMB will review available safety results of the trial and logistical issues such as accrual, retention, quality of clinical and laboratory data, and safety and implications of results of external studies. Unblinding envelopes will be made available to the DSMB should unblinding be requested.

The DSMB will make its recommendation to the Sponsor about dose escalation, halting or termination of the clinical trial. At each meeting the DSMB will review all available safety data and the DSMB will recommend to the Sponsor to:

- proceed with dose escalation
- pause enrolment pending either resolution of specific issues or amendment of the protocol as specified
- pause enrolment and all scheduled trial administrations pending either resolution of specific issues or amendment of the protocol as specified
- terminate the study

The DSMB, following a meeting to review the data, may recommend to the Sponsor and Principal Investigator not to permit further dose escalation if any of the following conditions are met:

- a subject experiences a SAE regarded as “probably” or “definitely” related to Opal-HIV-Gag(c) (i.e unblinded review of the event)
- any three subjects experience a similar severe adverse event (with a toxicity of at least grade 3) which is “probably” or “definitely” related to Opal-HIV-Gag(c) (i.e. unblinded review of the events)
- the Principal Investigator, DSMB and/or the Sponsor feel that consideration by the DSMB of a full interim safety summary is warranted following detection of a pattern of repeated or unresolved clinical AEs or laboratory abnormalities.

The clinical trial will be halted by the Principal Investigator and/or Sponsor for review and recommendation by the DSMB if:

- any three subjects experience a similar severe adverse event (with a toxicity of at least grade 3) which, in the opinion of the Principal Investigator, cannot be attributed to causes other than the IMP. (Note: transient and self-limiting events ‘expected’ in response to receipt of vaccines [such as fever] may not result in halting).
- any subject experiences a serious or severe adverse event, which, in the opinion of the Principal Investigator, contraindicates further dosing of additional subjects.

Refer to the DSMB Charter for further details of the review process.

11 SUBJECT COMPLETION / WITHDRAWAL

11.1 Subject Completion

A subject will be deemed to have completed the study once all trial procedures have been conducted. Any AEs or SAEs still ongoing at the time of the Exit Evaluation will be followed in accordance with Section 9.

11.2 Criteria for Premature Withdrawal from Treatment or the Study

Subjects have the right to withdraw from treatment or the study at any time for any reason. The Investigator must make every reasonable effort to keep each subject in the study except where termination or withdrawal is for reasons of safety. The Investigator also has the right to withdraw patients from treatment or the study in the event of intercurrent illness, AEs, pregnancy, treatment failure after a prescribed procedure, protocol violations, administrative reasons or other reasons.

It is understood by all concerned that an excessive rate of withdrawals from the study can render the study difficult to interpret. In particular, missing data on virological or histological assessments could significantly impact on the interpretation of the results; therefore, unnecessary withdrawal of subjects from the study should be avoided.

The reasons for withdrawal of the subject must be recorded on the CRF. The following are considered justifiable reasons for subject withdrawal:

- the need to take medication which may interfere with study measurements
- intolerable/unacceptable adverse experiences
- major violation or deviation of study protocol
- non-compliance of subject with protocol
- subject unwilling to proceed and/or consent is withdrawn
- withdrawal from the study is, in the Investigator's judgement, in the subject's best interest
- pregnancy of female study subject at any time during the study period (if applicable).

11.3 Withdrawal of subjects from Study Product

Section 9.6 provides guidance for dose modification or discontinuation of IMP in the event of AEs or abnormal laboratory values.

If a subject permanently discontinues dosing with IMP (for example, as a result of an AE), every attempt should be made to keep the subject in the study and continue to perform the required follow-up and procedures. The ideal is to continue to follow the subject for the full study period or until resolution of the AE. If it is not possible for the subject to remain in the study for the full study period, every attempt should be made to keep the subject in the study up to W16. If the subject remains in the study but off study treatment, all study procedures should continue as per protocol or equivalent assessments during the on-study period, such as pre-dosing study assessments at W4, 8 and 12.

If the subject will not remain in the study for all study related procedures, the Exit Evaluation should be performed wherever possible.

All subjects who discontinue IMP dosing should be followed for at least 30 days after the last dose of IMP in order to monitor subjects for possible post-treatment AEs which may occur after IMP has been discontinued.

11.4 Withdrawal of Subjects from the Study

Should a subject decide to withdraw from the study, all efforts will be made to complete and report the observations as thoroughly as possible.

If the reason for removal of a subject from the study is an AE or an abnormal laboratory test result, the principal reason will also be recorded on the CRF AE page. Where possible, subjects should be followed until the AE is resolved or the abnormal laboratory test has returned to normal.

11.5 Replacement of withdrawn subjects

Any subjects who discontinue in a clinical study of their own volition or by a decision of the Investigator are defined as “withdrawals”. Withdrawals may be replaced only upon consultation with the Sponsor. A replacement subject will be given the same last two digits of the withdrawn subject’s Subject Number but prefixed with ‘1’ replacing ‘0’ (e.g. 101 to replace 001), and he/she will receive the same treatment as the withdrawn subject if their treatment is still blinded, or otherwise a treatment by random allocation after consultation with the sponsor and study statistician.

11.6 Premature termination of the study

The study may be prematurely terminated by the ethics committee or relevant regulatory authorities, if the perception of the benefit/risk becomes unfavourable for continuation of the study.

Additionally, MDL reserves the right to terminate the study at any time on the basis of new information regarding safety or efficacy, or if study progress is unsatisfactory, or for other valid administrative or commercial reasons.

After such a decision is made, the Investigator should promptly inform the subjects and assure appropriate therapy and follow-up, and inform the relevant regulatory authorities and the ethics committee. All delivered study materials must be collected and all CRFs completed to the extent possible.

12 STATISTICAL ANALYSIS

12.1 Hypothesis

This is a first in human phase I study and, as such, there will be no formal hypotheses testing. The focus of the analysis will be to generate descriptive statistics for all the study endpoints. The descriptive statistics will be presented in tables and by graphs.

12.2 Sample Size Determination

Twenty seven subjects will be included in the study, which is within the recommended 20 to 80 subjects for a Phase I study (see Chow and Liu 2004[37], FDA general considerations for the clinical evaluation of drugs 1997[38]).

A possible safety signal will be indicated by three or more subjects with toxicity of at least grade 3 considered to be related to treatment among the 18 patients randomised to receive Opal-HIV-Gag(c) immunotherapy. If the true rate of severe treatment related toxicities is 5% or 10%, then the probability of three or more events being reported is 5.8% and 26.6% respectively. If the true rate of severe treatment related toxicity is greater than 20% and 30%, then the probability of three or more events being reported is greater than 72.9% and 94.0% respectively.

In the 18 Opal-HIV-Gag(c) subjects, a 95% confidence interval will be calculated around the observed rate of severe treatment related toxicities. If one, two or three events are observed among the 18 Opal-HIV-Gag(c) subjects, then the calculated 95% confidence intervals will be (0.1 to 27.3%), (1.4 to 34.7%) and (3.6 to 41.4%) respectively. A total of 18 Opal-HIV-Gag(c) subjects will give at least reasonable power to detect severe treatment related toxicities at rates of 20% or greater.

By including placebo recipients, laboratory assays assessing the immunogenicity of the HIV immunotherapy are subjected to more rigorous testing for validity and specificity. The inclusion of a placebo group with double blinding may also assist in reducing the potential for volunteers to make assumptions about the candidate material they are receiving and subsequent changes of behaviour.

12.3 Randomisation

Study treatments are presented in a double-blind manner and assigned at random to subjects in order to eliminate any selection or assessment bias that might occur. Eighteen subjects will receive Opal-HIV-Gag(c) and nine placebo. The randomisation process is described in Section 7.1.

In the event that a subject is incorrectly entered into the study and randomised, the subject may continue in the study if it is considered safe to do so after consultation with the Sponsor. On no account may a Screening Number or Subject Number be used twice; however, it is possible to replace subjects who discontinue (see Section 11.5).

12.4 Criteria for evaluation of study objectives

12.4.1 Definition of evaluation of study objective(s)

- Primary:
  - Safety will be examined through treatment-emergent AEs, vital signs and routine laboratory screening (haematology and clinical chemistry)
- Secondary:
  - Immunogenicity will be assessed through T-cell immunogenicity
  - Impact on HIV-1 infection will be assessed through HIV-1 viral load and CD4 T-cell counts

12.4.2 Analysis populations

The populations for analysis will include the following:

The Intent to Treat population will comprise all randomised subjects.

Safety Population: The safety population will include all subjects who received at least one dose of study medication. Only subjects with clear documentation that no study medication was received may be excluded. In the event of randomisation errors, subjects will be analysed according to the treatment they received.

12.4.2.1 Group comparability

Demographic and baseline information, including disease status (if applicable) and medical conditions, will be summarised and tabulated by treatment group. The purpose of these summaries is to characterise the study population and to describe any baseline imbalances.

12.4.2.2 Data analysis methods

The intent-to-treat population will be used for efficacy analyses and the safety population will be used for safety analyses. If no difference between the populations exists, only the intent-to-treat analysis will be conducted.

12.5 Statistical analysis

A detailed SAP in which all aspects of data analysis will be defined in detail will be prepared prior to inspection of any data by the biostatistician and prior to database lock.

The SAP will include details of the following:

- Definitions of analysis populations
- Description of all data transformations and data derivations to be used together with rationale and references
- Details of hypotheses to be tested (if any), together with treatment effects and corresponding confidence intervals to be estimated
- Details of methods for checking the appropriateness of the chosen statistical model
- Discussion of the use of baseline data to improve precision or to adjust estimates for potential baseline differences (e.g. analysis of covariance)
- Discussion of multiplicity and adjustment procedures if required
- Methods of dealing with missing values and outliers together with details of any sensitivity analyses to be performed
- Specification of any subgroup or interaction analyses
- Details of any planned interim analyses
- Example table and listing shells to indicate the proposed presentation of the data.

12.6 Analyses

12.6.1 Week 14 Interim Analysis

A planned interim analysis of safety and immunogenicity data will be conducted when all randomised study participants have reached W14. All randomised subjects will have received all IMP administrations and will have completed two weeks follow-up or have permanently withdrawn from the study. There is no intent to stop the study early since the W24 efficacy and safety data are also of interest. A study report will be prepared to present the study results of the W14 analysis.

12.6.2 Final Analysis

After the last study participant has completed W24, the safety and immunogenicity data will be analysed and a final clinical/statistical study report will be prepared.

12.6.3 Analysis of demographics

Demographic and other baseline characteristics will be described by assigned group and for the total of each of the derived study cohorts. Demographic characteristics of each study cohort will be tabulated.

12.6.4 Analysis of safety

12.6.4.1 Incidence of adverse events

All AEs will be coded using the Medical Dictionary for Regulatory Activities (MedDRA) version 8.1 or higher. AEs will be summarised according to the number of patients experiencing specific events, reported by system organ class, high level group term, high level term and preferred term. AEs will also be presented by severity and relationship to treatment. All data will be listed.

12.6.4.2 Clinical laboratory parameters

Routine samples are being collected for assessment of clinical chemistry and haematology. Summary tables at each time point and by toxicity grade will be presented by dose level. All data will be listed.

12.6.4.3 Vital signs

Vital signs are being assessed frequently across the duration of the study. For each sign (temperature, heart rate, respiratory rate, systolic and diastolic BP) summary tables will be produced. All data will be listed.

12.6.5 Analysis of efficacy

Efficacy is being assessed through the measurement of T-cell immunogenicity. For each variable, summary tables will be produced by time point and by dose level, and may be presented by clade. If appropriate, data will be summarised after log transformation. This will be described fully in the SAP. In addition, summary tables of the number (and percentage) of subjects with viral load and CD4+ T-cell counts will be presented by time point and by dose, and may be presented by clade. All data will be listed.

Immunogenicity of the immunotherapy will be determined based on comparisons between study treatment groups at W14. Changes in efficacy endpoints between the study treatment groups over the entire follow-up period may be the subject of informal statistical analyses which may involve post-hoc calculation of confidence intervals in order to further describe and characterise endpoints of interest. Analyses will compare study treatment groups at each time-point over the entire 24 week follow-up period in terms of appropriate summary statistics. Exploratory analyses will be used to examine the relationship between primary and secondary immunogenic endpoints.

12.6.6 Other Efficacy Analyses

Other efficacy analyses will be documented as part of the SAP.

12.6.7 Exploratory Analyses

Other exploratory analyses will be documented as part of the SAP.

13 GENERAL STUDY ADMINISTRATION

13.1 Ethical Aspects

13.1.1 Local regulations / Declaration of Helsinki

The Investigator will ensure that this study is conducted in full conformance with the protocol, the latest version of the "Declaration of Helsinki (and its amendments) and with the requirements of national drug and data protection laws of the countries in which the research is conducted (see Appendix B).

Studies conducted in European Union countries must fully adhere to the principles outlined in European Union's Clinical Trial Directives (2001/20/EC and 2005/28/EC).

In other countries, the Sponsor, the CRO, and the Investigators will ensure strict adherence to the provisions of the "Good Clinical Practice Guidelines" and national regulations.

13.1.2 Informed consent

It is the responsibility of the Investigator to obtain written informed consent from each individual participating in this study after adequate explanation of the aims, methods, objectives and potential hazards of the study prior to undertaking any study related procedures. The Investigator must also explain to the subject that they are completely free to refuse to enter the study or to withdraw from it at any time for any reason. The Investigator must utilise an Institutional Review Board (IRB) or IEC approved consent form for documenting written informed consent.

13.1.3 Premature withdrawal

If subjects discontinue study medication dosing, for example as a result of an AE, every attempt should be made to keep the subject in the study and continue to perform the required study related follow-up and procedures or equivalent assessments. If this is not possible or acceptable to the subject or Investigator, the subject may be withdrawn from the study. Refer to Section 11 for more detail.

13.1.4 Institutional Review Boards or Ethics Committees

This protocol and any accompanying material provided to the subject (such as subject information sheets or descriptions of the study used to obtain informed consent) will be submitted by the Investigator to an IRB or IEC. Approval from the committee must be obtained before starting the study, and should be documented in a letter to the Investigator specifying the protocol number and version and the date on which the committee met and granted the approval.

Any modifications made to the protocol after receipt of IRB or IEC approval must also be submitted by the Investigator to the committee in accordance with institutional procedures and regulatory requirements.

13.1.5 Conditions for modifying the protocol

Protocol modifications which could potentially adversely affect the safety of participating subjects or which alter the scope of the investigation, the scientific quality of the study, the experimental design, dosages, duration of therapy, assessment variables, the number of subjects treated or subject selection criteria, may be made only after consultation between an appropriate representative of the Sponsor and the Investigator.

Protocol modifications (amendments) must be prepared by a representative of the Sponsor and initially reviewed and approved by the responsible Medical Monitor and PI.

All protocol modifications must be submitted to the IRB or IEC in accordance with local requirements. Approval must be awaited before significant changes can be implemented i.e., if the risk

benefit ratio is affected and/or the modification represents a change in basic trial definitions such as objectives, design, sample size or outcome measures.

In the event of an emergency, the Investigator may institute any medical procedures deemed appropriate. However, all such procedures must be promptly reported to the Sponsor, the Medical Monitor and the IEC/IRB.

Administrative changes to the protocol are defined as minor corrections and/or clarifications that have no effect on the way the study is to be conducted, or on the safety of the subjects. These administrative changes will be agreed upon by the Sponsor and the Investigator and will be documented in a memorandum. The Investigator will then notify the IRB/IEC of such administrative changes.

13.1.6 Conditions for terminating the study

Both the Sponsor and the Investigator reserve the right to terminate the study at any time. Should this be necessary, the procedures will be arranged on an individual study basis after review and consultation by both parties. In terminating the study, the Sponsor and the Investigator will ensure that adequate consideration is given to the protection of the subject's interests.

13.2 Study Documentation, Case Report Forms and Record Keeping

13.2.1 Investigator's files / Retention of documents

The Investigator must maintain adequate and accurate records to enable the conduct of the study to be fully documented and the study data to be subsequently verified. These documents should be classified into two separate categories: (1) Investigator's Study File, and (2) subject clinical source documents.

The Investigator's Study File will contain the protocol/amendments, Case Report and Query Forms, IRB or IEC and governmental approval with correspondence, informed consent, IMP records, staff curriculum vitae and authorisation forms and other appropriate documents and correspondence.

Subject clinical source documents (usually defined by the project in advance to record key efficacy/safety parameters independent of the CRFs) would include subject hospital/clinic records, physician's and nurse's notes, appointment book, original laboratory reports, ECG, electroencephalography, X-ray, pathology and special assessment reports, consultant letters, etc. All clinical study documents must be retained by the Investigator until at least 2 years after the last approval of a marketing application in an ICH region (i.e. USA, Europe, or Japan) and until there are no pending or contemplated marketing applications in an ICH region; or if no application is filed or if the application is not approved for such indication, until 2 years after the investigation is discontinued and regulatory authorities have been notified. The Investigator must notify the Sponsor prior to destroying any clinical study records.

Should the Investigator wish to assign the study records to another party or move them to another location, the Sponsor must be notified in advance.

If the Investigator cannot guarantee this archiving requirement at the study site for any or all of the documents, special arrangements must be made between the Investigator and the Sponsor to store these in a sealed container(s) outside of the site so that they can be returned sealed to the Investigator in case of a regulatory audit. Where source documents are required for the continued care of the subject, appropriate copies should be made for storage outside of the site.

13.2.2 Background data

The Investigator shall supply the Sponsor, on request, with any required background data from the study documentation or clinic records. This is particularly important when CRFs are illegible or when errors in data transcription are suspected. In case of special problems and/or governmental queries or

requests for audit inspections, it is also necessary to have access to the complete study records, provided that subject confidentiality is protected.

13.2.3 Inspections

The Investigator should understand that source documents for this trial should be made available to appropriately qualified personnel from the Sponsor or its representative or to regulatory authority or health authority inspectors after appropriate notification. The verification of the CRF data will be by direct inspection of source documents.

13.2.4 Case Report Forms

For each subject enrolled (consented), CRFs must be completed and signed by the Investigator. This also applies to records for those subjects who fail to complete the study (even during a pre-randomisation screening period if a CRF was initiated). If a subject withdraws from the study, the reason must be noted on the CRF. If a subject is withdrawn from the study because of a treatment-limiting AE, thorough efforts should be made to clearly document the outcome.

All forms should be typed or filled out using a black ball-point pen, and must be legible. Errors should be crossed out but not obliterated, the correction inserted, and the change initialled and dated by the PI or his/her authorised delegate. The CRFs, as well as the protocol, are confidential. The CRFs remain the property of the sponsor at all times.

13.3 Monitoring the Study

In accordance with ICH-GCP guidelines, the study monitor must have direct access to the Investigator's source documentation in order to verify the data recorded in the CRFs for consistency.

It is understood that the responsible monitor, as a Sponsor representative, will contact and visit the Investigator regularly and that he/she will be allowed, on request, to inspect the various records of the trial (CRFs and other pertinent data) provided that subject confidentiality is maintained in accordance with local requirements.

It will be the monitor's responsibility to inspect the CRFs at regular intervals throughout the study, to verify the adherence to the protocol and the completeness, consistency and accuracy of the data being entered on them. Where local regulations permit, the monitor should have access to laboratory test reports and other subject records needed to verify the entries on the CRF. The Investigator agrees to cooperate with the monitor to ensure that any problems detected in the course of these monitoring visits are resolved.

13.4 Confidentiality of Trial Documents and Subject Records

The Investigator must assure the subjects' anonymity will be maintained and that their identities are protected from unauthorised parties. On CRFs or other documents submitted to the Sponsor, subjects should not be identified by their names, but by the subject's initials and Screening or Subject Numbers. The Investigator should keep a subject enrolment log showing codes, names and addresses. Documents not for submission to the Sponsor (eg. subject's written consent forms), should be maintained by the Investigator in strict confidence.

All information concerning the study treatment and the Sponsor and its operation, such as patent applications, formulae, manufacturing processes, basic scientific data and material not previously published are considered confidential and shall remain the sole property of the Sponsor. The Investigator agrees to use this information only in accomplishing the study and will not use it for any other purposes without written consent from the Sponsor.

13.5 Publication of Data and Protection of Trade Secrets

In accord with standard editorial and ethical practice, the Sponsor will support publication of trials only in their entirety and not as individual centre data.

The results of this study may be published or presented at scientific meetings. If this is envisaged, the Investigator agrees to submit all manuscripts to the Sponsor 60 days or abstracts to the Sponsor 5 days prior to submission. This allows the Sponsor to protect proprietary information and to provide comments based on information from other studies that may not yet be available to the Investigator.

Any formal publication of the study in which input of the Sponsor's personnel exceeded that of conventional monitoring will be considered as a joint publication by the Investigator and the appropriate Sponsor personnel. Authorship will be determined by mutual agreement prior to the start of the study. Additional authors will be agreed prior to the completion of the study.

13.6 Anticipated Subject Accrual and Duration of the Study

The anticipated patient accrual will be 27 subjects in 6 months. The Investigator should continually compare the actual and expected accrual rates, and make every effort to ensure that they are as closely matched as possible. If the Investigator anticipates major problems with recruitment, or delay in the expected completion date, he/she should discuss this with the Sponsor as early as possible.

14 REFERENCES

1. UNAIDS Report on the global HIV/AIDS epidemic 2008 UNAIDS/08.25E / JC1510E: http://www.unaids.org/en/KnowledgeCentre/HIVData/GlobalReport/2008/. 2008.
2. Rosenberg ES, Billingsley JM, Caliendo AM, Boswell SL, Sax PE, Kalams SA, et al. Vigorous HIV-1-specific CD4+ T cell responses associated with control of viremia. *Science* 1997;278(5342):1447-50.
3. Jin X, Bauer DE, Tuttleton SE, Lewin S, Gettie A, Blanchard J, et al. Dramatic rise in plasma viremia after CD8(+) T cell depletion in simian immunodeficiency virus-infected macaques. *J Exp Med* 1999;189(6):991-8.
4. Schmitz JE, Kuroda MJ, Santra S, Sasseville VG, Simon MA, Lifton MA, et al. Control of viremia in simian immunodeficiency virus infection by CD8+ lymphocytes. *Science* 1999;283(5403):857-60.
5. Chea S, Dale CJ, De Rose R, Ramshaw IA, Kent SJ. Enhanced cellular immunity in macaques following a novel peptide immunotherapy. *J Virol* 2005;79(6):3748-57.
6. Friedrich TC, Dodds EJ, Yant LJ, Vojnov L, Rudersdorf R, Cullen C, et al. Reversion of CTL escape-variant immunodeficiency viruses in vivo. *Nat Med* 2004;10(3):275-81.
7. Goulder PJ, Watkins DI. HIV and SIV CTL escape: implications for vaccine design. *Nat Rev Immunol* 2004;4(8):630-40.
8. Goepfert PA, Lumm W, Farmer P, Matthews P, Prendergast A, Carlson JM, et al. Transmission of HIV-1 Gag immune escape mutations is associated with reduced viral load in linked recipients. *J Exp Med* 2008;205(5):1009-17.
9. Kiepiela P, Ngumbela K, Thobakgale C, Ramduth D, Honeyborne I, Moodley E, et al. CD8+ T-cell responses to different HIV proteins have discordant associations with viral load. *Nat Med* 2007;13(1):46-53.
10. Maecker HT, Dunn HS, Suni MA, Khatamzas E, Pitcher CJ, Bunde T, et al. Use of overlapping peptide mixtures as antigens for cytokine flow cytometry. *J Immunol Methods* 2001;255(1-2):27-40.
11. Geels MJ, Dubey SA, Anderson K, Baan E, Bakker M, Pollakis G, et al. Broad cross-clade T-cell responses to gag in individuals infected with human immunodeficiency virus type 1 non-B clades (A to G): importance of HLA anchor residue conservation. *J Virol* 2005;79(17):11247-58.
12. Goujard C, Marcellin F, Hendel-Chavez H, Burgard M, Meiffredy V, Venet A, et al. Interruption of antiretroviral therapy initiated during primary HIV-1 infection: impact of a therapeutic vaccination strategy combined with interleukin (IL)-2 compared with IL-2 alone in the ANRS 095 Randomized Study. *AIDS Res Hum Retroviruses* 2007;23(9):1105-13.
13. Autran B, Murphy RL, Costagliola D, Tubiana R, Clotet B, Gatell J, et al. Greater viral rebound and reduced time to resume antiretroviral therapy after therapeutic immunization with the ALVAC-HIV vaccine (vCP1452). *Aids* 2008;22(11):1313-22.
14. Kinloch-de Loes S, Hoen B, Smith DE, Autran B, Lampe FC, Phillips AN, et al. Impact of therapeutic immunization on HIV-1 viremia after discontinuation of antiretroviral therapy initiated during acute infection. *J Infect Dis* 2005;192(4):607-17.
15. Hanke T, Goonetilleke N, McMichael AJ, Dorrell L. Clinical experience with plasmid DNA- and modified vaccinia virus Ankara-vectored human immunodeficiency virus type 1 clade A vaccine focusing on T-cell induction. *J Gen Virol* 2007;88(Pt 1):1-12.
16. Dorrell L, Yang H, Ondondo B, Dong T, di Gleria K, Suttill A, et al. Expansion and diversification of virus-specific T cells following immunization of human immunodeficiency virus type 1 (HIV-1)-infected individuals with a recombinant modified vaccinia virus Ankara/HIV-1 Gag vaccine. *J Virol* 2006;80(10):4705-16.
17. Dorrell L, Williams P, Suttill A, Brown D, Roberts J, Conlon C, et al. Safety and tolerability of recombinant modified vaccinia virus Ankara expressing an HIV-1 gag/multiepitope immunogen (MVA.HIVA) in HIV-1-infected persons receiving combination antiretroviral therapy. *Vaccine* 2007;25(17):3277-83.

18. Ondondo BO, Yang H, Dong T, di Gleria K, Suttill A, Conlon C, et al. Immunisation with recombinant modified vaccinia virus Ankara expressing HIV-1 gag in HIV-1-infected subjects stimulates broad functional CD4+ T cell responses. *Eur J Immunol* 2006;36(10):2585-94.
19. Buchbinder SP, Mehrotra DV, Duerr A, Fitzgerald DW, Mogg R, Li D, et al. Efficacy assessment of a cell-mediated immunity HIV-1 vaccine (the Step Study): a double-blind, randomised, placebo-controlled, test-of-concept trial. *Lancet* 2008;372(9653):1881-93.
20. Kran AM, Sommerfelt MA, Sorensen B, Nyhus J, Baksaas I, Bruun JN, et al. Reduced viral burden amongst high responder patients following HIV-1 p24 peptide-based therapeutic immunization. *Vaccine* 2005;23(31):4011-5.
21. Kran AM, Sorensen B, Sommerfelt MA, Nyhus J, Baksaas I, Kvale D. Long-term HIV-specific responses and delayed resumption of antiretroviral therapy after peptide immunization targeting dendritic cells. *Aids* 2006;20(4):627-30.
22. Connolly NC, Whiteside TL, Wilson C, Kondragunta V, Rinaldo CR, Riddler SA. Therapeutic immunization with human immunodeficiency virus type 1 (HIV-1) peptide-loaded dendritic cells is safe and induces immunogenicity in HIV-1-infected individuals. *Clin Vaccine Immunol* 2008;15(2):284-92.
23. Kundu SK, Engleman E, Benike C, Shapero MH, Dupuis M, van Schooten WC, et al. A pilot clinical trial of HIV antigen-pulsed allogeneic and autologous dendritic cell therapy in HIV-infected patients. *AIDS Res Hum Retroviruses* 1998;14(7):551-60.
24. Ide F, Nakamura T, Tomizawa M, Kawana-Tachikawa A, Odawara T, Hosoya N, et al. Peptide-loaded dendritic-cell vaccination followed by treatment interruption for chronic HIV-1 infection: a phase 1 trial. *J Med Virol* 2006;78(6):711-8.
25. Primers IV. IAVI VAX Primers: Understanding the genetic variation of HIV.
, August 2008.
26. Stratov I, Dale CJ, Chea S, McCluskey J, Kent SJ. Induction of T-cell immunity to antiretroviral drug-resistant human immunodeficiency virus type 1. *J Virol* 2005;79(12):7728-37.
27. De Rose R, Fernandez CS, Smith MZ, Batten CJ, Alcantara S, Peut V, et al. Control of viremia and prevention of AIDS following immunotherapy of SIV-infected macaques with peptide-pulsed blood. *PLoS Pathog* 2008;4(5):e1000055.
28. De Rose R, Mason RD, Loh LY, Puet V, Smith M, Fernandez CS, et al. Safety, immunogenicity and efficacy of peptide-pulsed cellular immunotherapy in macaques. *J Med Primatol* 2008;37(Supplement 2).
29. De Rose R, Fernandez CS, Loh L, Peut V, Mason RD, Alcantara S, et al. Delivery of immunotherapy with peptide-pulsed blood in macaques. *Virology* 2008;378(2):201-4.
30. OECD Principles of Good Laboratory Practice C (97)186/Final Directive 2004/10/EC, 2004.
31. ICH Topic S 6. Preclinical Safety Evaluation of Biotechnology-Derived Pharmaceuticals (CPMP/ICH/302/95), 1998.
32. CPMP Note for Guidance on the Preclinical Pharmacological and Toxicological Testing of Vaccines, CPMP/SWP/465/95, 1997
33. WHO Guidelines: Nonclinical Evaluation of Vaccines, WHO/BS03, 1969.
34. FDA Guidance for Industry. Estimating the Maximum Safe Starting Dose in Initial Clinical Trials for Therapeutics in Adult Healthy Volunteers, 2005.
35. Windrum P, Morris T, Drake M, Niederwiser D, Ruutu T, Subcommittee. obotECLWPC. Variation in dimethyl sulfoxid use in stem cell transplantation: a survey of EBMT centres. *Bone Marrow Transplantation* 2005;36:601-603.
36. Sauer- Heilborn A, Kadidlo D, McCullough J. Patient care during infusion of hematopoietic progenitor cells. *Transfusion* 2004;44:907-916.
37. Sheing-Chung Chow J-pL. *Design and Analysis of Clinical trials: Concepts and Methodologies.* Second ed: John Wiley & Sons Inc., 2004.
38. CDER F. *Guidance for Industry: General considerations for the clinical evaluation of drugs*, 1997.

APPENDIX A AIDS CLINICAL TRIAL GROUP (ACTG) GRADING SCALE

DIVISION OF AIDS TABLE FOR GRADING THE SEVERITY OF ADULT AND PEDIATRIC ADVERSE EVENTS PUBLISH DATE: DECEMBER, 2004

General Instructions

Grading Adult and Paediatric AEs

The Division of AIDS AE grading table includes parameters for grading both Adult and Paediatric AEs. When a single set of parameters is not appropriate for grading specific types of AEs for both Adult and Paediatric populations, separate sets of parameters for Adult and/or Paediatric populations (with specified respective age ranges) are given in the table. If there is no distinction in the table between Adult and Paediatric values for a type of AE, then the single set of parameters listed is to be used for grading the severity of both Adult and Paediatric events of that type.

Determining Severity Grade

If the severity of an AE could fall under either one of two grades (e.g., the severity of an AE could be either Grade 2 or Grade 3), select the higher of the two grades for the AE.

Definitions

Basic Self-care Functions	Adult<br>Activities such as bathing, dressing, toileting, transfer/movement, continence, and feeding.<br>Young Children<br>Activities that are age and culturally appropriate (e.g., feeding self with culturally appropriate eating implement).
LLN	Lower limit of normal
Medical Intervention	Use of pharmacologic or biologic agent(s) for treatment of an AE.
NA	Not Applicable
Operative Intervention	Surgical OR other invasive mechanical procedures.
PR Interval	The PR interval is the time (in seconds) from the beginning of the P wave (onset of atrial depolarization) to the beginning of the QRS complex (onset of ventricular depolarization).
Qtc	Corrected Q-T interval: The Q-T interval represents the time for both ventricular depolarization and repolarization to occur, and therefore roughly estimates the duration of an average ventricular action potential.
ULN	Upper limit of normal
Usual Social & Functional Activities	Adult<br>Adaptive tasks and desirable activities, such as going to work, shopping, cooking, use of transportation, pursuing a hobby, etc.<br>Young Children<br>Activities that are age and culturally appropriate (e.g., social interactions, play activities, learning tasks, etc.).

Clinical				
**Parameter**	**Grade 1 Mild**	**Grade 2 Moderate**	**Grade 3 Severe**	**Grade 4 Potentially life-threatening**
Clinical AE NOT identified elsewhere in this DAIDS AE grading table	Symptoms causing no or minimal interference with usual social & functional activities*	Symptoms causing greater than minimal interference with usual social & functional activities	Symptoms causing inability to perform usual social & functional activities	Symptoms causing inability to perform basic self-care functions** OR medical or operative intervention indicated to prevent permanent impairment, persistent disability, or death
**Systemic**				
Acute systemic allergic reaction	Localised urticaria (wheals) with no medical intervention indicated	Localised urticaria with medical intervention indicated OR Mild angioedema with no medical intervention indicated	Generalised urticaria OR Angioedema with medical intervention indicated OR Symptomatic mild bronchospasm	Acute anaphylaxis OR Life-threatening bronchospasm OR Laryngeal edema
Chills	Symptoms causing no or minimal interference with usual social & functional activities	Symptoms causing greater than minimal interference with usual social & functional activities	Symptoms causing inability to perform usual social & functional activities	NA
Fatigue<br>Malaise	Symptoms causing no or minimal interference with usual social & functional activities	Symptoms causing greater than minimal interference with usual social & functional activities	Symptoms causing inability to perform usual social & functional activities	Incapacitating fatigue/ malaise symptoms causing inability to perform basic self-care functions
Fever (nonaxillary)	37.7 – 38.6°C	38.7 – 39.3°C	39.4 – 40.5°C	>40.5°C
Pain (indicate body site)<br>DO NOT use for pain due to injection (See Injection Site Reactions: Injection site pain)<br>See also Headache, Arthralgia, and Myalgia	Pain causing no or minimal interference with usual social & functional activities	Pain causing greater than minimal interference with usual social & functional activities	Pain causing inability to perform usual social & functional activities	Disabling pain causing inability to perform basic self-care functions OR Hospitalisation (other than emergency room visit) indicated
Unintentional weight loss	NA	5 – 9% loss in body weight from baseline	10 – 19% loss in body weight from baseline	≥20% loss in body weight from baseline OR Aggressive intervention indicated (e.g., tube feeding or total parenteral nutrition [TPN])

*** Basic Self-care Functions – Adult**: Activities such as bathing, dressing, toileting, transfer/movement, continence, and feeding. **Young Children**: Activities that are age and culturally appropriate (e.g., feeding self with culturally appropriate eating implement).
**** Usual Social & Functional Activities – Adult**: Adaptive tasks and desirable activities, such as going to work, shopping, cooking, use of transportation, pursuing a hobby, etc. **Young Children**: Activities that are age and culturally appropriate (e.g., social interactions, play activities, learning tasks, etc.).

Clinical				
**Parameter**	**Grade 1 Mild**	**Grade 2 Moderate**	**Grade 3 Severe**	**Grade 4 Potentially life-threatening**
**Infection**				
Infection (any other than HIV infection)	Localised, no systemic antimicrobial treatment indicated AND Symptoms causing no or minimal interference with usual social & functional activities	Systemic antimicrobial treatment indicated OR Symptoms causing greater than minimal interference with usual social & functional activities	Systemic antimicrobial treatment indicated AND Symptoms causing inability to perform usual social & functional activities OR Operative intervention (other than simple incision and drainage) indicated	Life-threatening consequences (e.g., septic shock)
**Injection site reactions**				
Injection site pain (pain without touching) Or Tenderness (pain when area is touched)	Pain/tenderness causing no or minimal limitation of use of limb	Pain/tenderness limiting use of limb OR Pain/tenderness causing greater than minimal interference with usual social & functional activities	Pain/tenderness causing inability to perform usual social & functional activities	Pain/tenderness causing inability to perform basic self-care function OR Hospitalisation (other than emergency room visit) indicated for management of pain/tenderness
Injection site reaction (localised): Adult >15 years	Erythema OR Induration of 5x5 cm – 9x9 cm (or 25 cm² – 81 cm²)	Erythema OR Induration OR Edema >9 cm any diameter (or >81 cm²)	Ulceration OR Secondary infection OR Phlebitis OR Sterile abscess OR Drainage	Necrosis (involving dermis and deeper tissue)
Injection site reaction (localised): Paediatric ≤15 years	Erythema OR Induration OR Edema present but <2.5 cm diameter	Erythema OR Induration OR Edema >2.5 cm diameter but <50% surface area of the extremity segment (e.g., upper arm/thigh)	Erythema OR Induration OR Edema involving ≥50% surface area of the extremity segment (e.g., upper arm/thigh) OR Ulceration OR Secondary infection OR Phlebitis OR Sterile abscess OR Drainage	Necrosis (involving dermis and deeper tissue)
Pruritis associated with injection See also Skin: Pruritis (itching - no skin lesions)	Itching localised to injection site AND Relieved spontaneously or with <48 hours treatment	Itching beyond the injection site but not generalised OR Itching localised to injection site requiring ≥48 hours treatment	Generalised itching causing inability to perform usual social & functional activities	NA

*** Basic Self-care Functions – Adult**: Activities such as bathing, dressing, toileting, transfer/movement, continence, and feeding. **Young Children**: Activities that are age and culturally appropriate (e.g., feeding self with culturally appropriate eating implement).
**** Usual Social & Functional Activities – Adult**: Adaptive tasks and desirable activities, such as going to work, shopping, cooking, use of transportation, pursuing a hobby, etc. **Young Children**: Activities that are age and culturally appropriate (e.g., social interactions, play activities, learning tasks, etc.).

Clinical				
**Parameter**	**Grade 1 Mild**	**Grade 2 Moderate**	**Grade 3 Severe**	**Grade 4 Potentially life-threatening**
**Skin – Dermatological**				
Alopecia	Thinning detectable by study participant (or by caregiver for young children and disabled adults)	Thinning or patchy hair loss detectable by health care provider	Complete hair loss	NA
Cutaneous reaction – rash	Localised macular rash	Diffuse macular, maculopapular, or morbilliform rash OR Target lesions	Diffuse macular, maculopapular, or morbilliform rash with vesicles or limited number of bullae OR Superficial ulcerations of mucous membrane limited to one site	Extensive or generalized bullous lesions OR Stevens-Johnson syndrome OR Ulceration of mucous membrane involving two or more distinct mucosal sites OR Toxic epidermal necrolysis
Hyperpigmentation	Slight or localised	Marked or generalised	NA	NA
Hypopigmentation	Slight or localised	Marked or generalised	NA	NA
Pruritis (itching – no skin lesions) (See also Injection Site Reactions: Pruritis associated with injection)	Itching causing no or minimal interference with usual social & functional activities	Itching causing greater than minimal interference with usual social & functional activities	Itching causing inability to perform usual social & functional activities	NA
**Cardiovascular**				
Cardiac arrhythmia (general) (By ECG or physical exam)	Asymptomatic AND No intervention indicated	Asymptomatic AND Non-urgent medical intervention indicated	Symptomatic, non-life-threatening AND Non urgent medical intervention indicated	Life-threatening arrhythmia OR Urgent intervention indicated
Cardiacischemia/ infarction	NA	NA	Symptomatic ischemia (stable angina) OR Testing consistent with ischemia	Unstable angina OR Acute myocardial infarction
Haemorrhage (significant acute blood loss)	NA	Symptomatic AND No transfusion indicated	Symptomatic AND Transfusion of ≤2 units packed RBCs (for children, ≤10 cubic centimetres (cc)/kg) indicated	Life-threatening hypotension OR Transfusion of >2 units packed RBCs (for children, >10 cc/kg) indicated

*** Basic Self-care Functions – Adult**: Activities such as bathing, dressing, toileting, transfer/movement, continence, and feeding. **Young Children**: Activities that are age and culturally appropriate (e.g., feeding self with culturally appropriate eating implement).
**** Usual Social & Functional Activities – Adult**: Adaptive tasks and desirable activities, such as going to work, shopping, cooking, use of transportation, pursuing a hobby, etc. **Young Children**: Activities that are age and culturally appropriate (e.g., social interactions, play activities, learning tasks, etc.).

Clinical				
**Parameter**	**Grade 1 Mild**	**Grade 2 Moderate**	**Grade 3 Severe**	**Grade 4 Potentially life-threatening**
Hypertension: Adult >17 years (with repeat testing at same visit)	>140 – 159 mmHg systolic OR >90 – 99 mmHg diastolic	>160 – 179 mmHg systolic OR >100 – 109 mmHg diastolic	>180 mmHg systolic OR >110 mmHg diastolic	Life-threatening consequences (e.g., malignant hypertension) OR Hospitalisation indicated (other than emergency room visit)
Hypertension: Paediatric ≤17 years (with repeat testing at same visit)	NA	91st – 94th percentile adjusted for age, height, and gender (systolic and/or diastolic)	(95th percentile adjusted for age, height, and gender (systolic and/or diastolic)	Life-threatening consequences (e.g., malignant hypertension) OR Hospitalisation indicated (other than emergency room visit)
Hypotension	NA	Symptomatic, corrected with oral fluid replacement	Symptomatic, IV fluids indicated	Shock requiring use of vasopressors or mechanical assistance to maintain BP
Pericardial effusion	Asymptomatic, small effusion requiring no intervention	Asymptomatic, moderate or larger effusion requiring no intervention	Effusion with non-life-threatening physiologic consequences OR Effusion with non-urgent intervention indicated	Life-threatening consequences (e.g., tamponade) OR Urgent intervention indicated
Prolonged PR interval: Adult >16 years	PR interval 0.21 – 0.25 sec	PR interval >0.25 sec	Type II 2nd degree Atrioventricular (AV) block OR Ventricular pause >3.0 sec	Complete AV block
Prolonged PR interval: Paediatric ≤16 Years	1st degree AV block (PR >normal for age and rate)	Type I 2nd degree AV block	Type II 2nd degree AV block	Complete AV block
Prolonged QTc: Adult >16 years	Asymptomatic, QTc interval 0.45 – 0.47 sec OR Increase interval <0.03 sec above baseline	Asymptomatic, QTc interval 0.48 – 0.49 sec OR Increase in interval 0.03 – 0.05 sec above baseline	Asymptomatic, QTc interval ≥0.50 sec OR Increase in interval ≥0.06 sec above baseline	Life-threatening consequences, e.g. Torsade de pointes or other associated serious ventricular dysrhythmia
Prolonged QTc: Paediatric ≤16 years	Asymptomatic, QTc interval 0.450 – 0.464 sec	Asymptomatic, QTc interval 0.465 – 0.479 sec	Asymptomatic, QTc interval ≥0.480 sec	Life-threatening consequences, e.g. Torsade de pointes or other associated serious ventricular dysrhythmia

*** Basic Self-care Functions – Adult**: Activities such as bathing, dressing, toileting, transfer/movement, continence, and feeding. **Young Children**: Activities that are age and culturally appropriate (e.g., feeding self with culturally appropriate eating implement).
**** Usual Social & Functional Activities – Adult**: Adaptive tasks and desirable activities, such as going to work, shopping, cooking, use of transportation, pursuing a hobby, etc. **Young Children**: Activities that are age and culturally appropriate (e.g., social interactions, play activities, learning tasks, etc.).

Clinical				
**Parameter**	**Grade 1 Mild**	**Grade 2 Moderate**	**Grade 3 Severe**	**Grade 4 Potentially life-threatening**
Thrombosis/embolism	NA	Deep vein thrombosis AND No intervention indicated (e.g., anticoagulation, lysis filter, invasive procedure)	Deep vein thrombosis AND Intervention indicated (e.g., anticoagulation, lysis filter, invasive procedure)	Embolic event (e.g., pulmonary embolism, life-threatening thrombus)
Vasovagal episode (associated with a procedure of any kind)	Present without loss of consciousness	Present with transient loss of consciousness	NA	NA
Ventricular dysfunction (congestive heart failure)	NA	Asymptomatic diagnostic finding AND intervention indicated	New onset with symptoms OR Worsening symptomatic congestive heart failure	Life-threatening congestive heart failure
**Gastrointestinal**				
Anorexia	Loss of appetite without decreased oral intake	Loss of appetite associated with decreased oral intake without significant weight loss	Loss of appetite associated with significant weight loss	Life-threatening consequences OR Aggressive intervention indicated (e.g., tube feeding or TPN)
Ascites	Asymptomatic	Symptomatic AND Intervention indicated (e.g., diuretics or therapeutic paracentesis)	Symptomatic despite intervention	Life-threatening consequences
Cholecystitis	NA	Symptomatic AND Medical intervention indicated	Radiologic, endoscopic, or operative intervention indicated	Life-threatening consequences (e.g., sepsis or perforation)
Constipation	NA	Persistent constipation requiring regular use of dietary modifications, laxatives, or enemas	Obstipation with manual evacuation indicated	Life-threatening consequences (e.g., obstruction)
Diarrhoea: Adult and Paediatric ≥ 1 year	Transient or intermittent episodes of unformed stools OR Increase of ≤3 stools over baseline per 24-hour period	Persistent episodes of unformed to watery stools OR Increase of 4 – 6 stools over baseline per 24 hour period	Bloody diarrhoea OR Increase of ≥7 stools per 24-hour period OR Intravenous (IV) fluid replacement indicated	Life-threatening consequences (e.g., hypotensive shock)
Diarrhoea: Paediatric <1 year	Liquid stools (more unformed than usual) but usual number of stools	Liquid stools with increased number of stools OR Mild dehydration	Liquid stools with moderate dehydration	Liquid stools resulting in severe dehydration with aggressive rehydration indicated OR Hypotensive shock

*** Basic Self-care Functions – Adult**: Activities such as bathing, dressing, toileting, transfer/movement, continence, and feeding. **Young Children**: Activities that are age and culturally appropriate (e.g., feeding self with culturally appropriate eating implement).
**** Usual Social & Functional Activities – Adult**: Adaptive tasks and desirable activities, such as going to work, shopping, cooking, use of transportation, pursuing a hobby, etc. **Young Children**: Activities that are age and culturally appropriate (e.g., social interactions, play activities, learning tasks, etc.).

Clinical				
**Parameter**	**Grade 1 Mild**	**Grade 2 Moderate**	**Grade 3 Severe**	**Grade 4 Potentially life-threatening**
Dysphagia-Odynophagia	Symptomatic but able to eat usual diet	Symptoms causing altered dietary intake without medical intervention indicated	Symptoms causing severely altered dietary intake with medical intervention indicated	Life-threatening reduction in oral intake
Mucositis/stomatitis (clinical exam)<br>Indicate site (e.g., larynx, oral)<br>See Genitourinary for Vulvovaginitis<br>See also Dysphagia-Odynophagia and Proctitis	Erythema of the Mucosa	Patchy pseudomembranes or ulcerations	Confluent pseudomembranes or ulcerations OR Mucosal bleeding with minor trauma	Tissue necrosis OR Diffuse spontaneous mucosal bleeding OR Life-threatening consequences (e.g., aspiration, choking)
Nausea	Transient (<24 hours) or intermittent nausea with no or minimal interference with oral intake	Persistent nausea resulting in decreased oral intake for 24 – 48 hours	Persistent nausea resulting in minimal oral intake for >48 hours OR Aggressive dehydration indicated (e.g., IV fluids)	Life-threatening consequences (e.g., hypotensive shock)
Pancreatitis	NA	Symptomatic AND Hospitalisation not indicated (other than emergency room visit)	Symptomatic AND Hospitalisation indicated (other than emergency room visit)	Life-threatening consequences (e.g., circulatory failure, haemorrhage, sepsis)
Proctitis (functional-symptomatic)<br>Also see Mucositis/stomatitis for clinical exam	Rectal discomfort AND No intervention Indicated	Symptoms causing greater than minimal interference with usual social & functional activities OR Medical intervention indicated	Symptoms causing inability to perform usual social & functional activities OR Operative intervention indicated	Life-threatening consequences (e.g., perforation)
Vomiting	Transient or intermittent vomiting with no or minimal interference with oral intake	Frequent episodes of vomiting with no or mild dehydration	Persistent vomiting resulting in orthostatic hypotension OR Aggressive dehydration indicated (e.g., IV fluids)	Life-threatening consequences (e.g., hypotensive shock)

*** Basic Self-care Functions – Adult**: Activities such as bathing, dressing, toileting, transfer/movement, continence, and feeding. **Young Children**: Activities that are age and culturally appropriate (e.g., feeding self with culturally appropriate eating implement).
**** Usual Social & Functional Activities – Adult**: Adaptive tasks and desirable activities, such as going to work, shopping, cooking, use of transportation, pursuing a hobby, etc. **Young Children**: Activities that are age and culturally appropriate (e.g., social interactions, play activities, learning tasks, etc.).

Clinical				
**Parameter**	**Grade 1 Mild**	**Grade 2 Moderate**	**Grade 3 Severe**	**Grade 4 Potentially life-threatening**
**Neurologic**				
Alteration in personality-behaviour or in mood (e.g., agitation, anxiety, depression, mania, psychosis)	Alteration causing no or minimal interference with usual social & functional activities	Alteration causing greater than minimal interference with usual social & functional activities	Alteration causing inability to perform usual social & functional activities	Behaviour potentially harmful to self or others (e.g., suicidal and homicidal ideation or attempt, acute psychosis) OR Causing inability to perform basic self-care functions
Altered Mental Status For Dementia, see Cognitive and behavioral/attentional disturbance (including dementia and attention deficit disorder)	Changes causing no or minimal interference with usual social & functional activities	Mild lethargy or somnolence causing greater than minimal interference with usual social & functional activities	Confusion, memory impairment, lethargy, or somnolence causing inability to perform usual social & functional activities	Delirium OR Obtundation, OR Coma
Ataxia	Asymptomatic ataxia detectable on exam OR Minimal ataxia causing no or minimal interference with usual social & functional activities	Symptomatic ataxia causing greater than minimal interference with usual social & functional activities	Symptomatic ataxia causing inability to perform usual social & functional activities	Disabling ataxia causing inability to perform basic self-care functions
Cognitive and behavioural/attentional disturbance (including dementia and attention deficit disorder)	Disability causing no or minimal interference with usual social & functional activities OR Specialised resources not indicated	Disability causing greater than minimal interference with usual social & functional activities OR Specialised resources on part-time basis indicated	Disability causing inability to perform usual social & functional activities OR Specialised resources on a full-time basis indicated	Disability causing inability to perform basic self-care functions OR Institutionalization indicated
CNS ischemia (acute)	NA	NA	Transient Ischemic Attack	Cerebral Vascular Accident (cerebral vascular accident, stroke) with neurological deficit

*** Basic Self-care Functions – Adult**: Activities such as bathing, dressing, toileting, transfer/movement, continence, and feeding. **Young Children**: Activities that are age and culturally appropriate (e.g., feeding self with culturally appropriate eating implement).
**** Usual Social & Functional Activities – Adult**: Adaptive tasks and desirable activities, such as going to work, shopping, cooking, use of transportation, pursuing a hobby, etc. **Young Children**: Activities that are age and culturally appropriate (e.g., social interactions, play activities, learning tasks, etc.).

Clinical				
**Parameter**	**Grade 1 Mild**	**Grade 2 Moderate**	**Grade 3 Severe**	**Grade 4 Potentially life-threatening**
Developmental delay – Paediatric ≤16 years	Mild developmental delay, either motor or cognitive, as determined by comparison with a developmental screening tool appropriate for the setting	Moderate developmental delay, either motor or cognitive, as determined by comparison with a developmental screening tool appropriate for the setting	Severe developmental delay, either motor or cognitive, as determined by comparison with a developmental screening tool appropriate for the setting	Developmental regression, either motor or cognitive, as determined by comparison with a developmental screening tool appropriate for the setting
Headache	Symptoms causing no or minimal interference with usual social & functional activities	Symptoms causing greater than minimal interference with usual social & functional activities	Symptoms causing inability to perform usual social & functional activities	Symptoms causing inability to perform basic self-care functions OR Hospitalisation indicated (other than emergency room visit) OR Headache with significant impairment of alertness or other neurologic function
Insomnia	NA	Difficulty sleeping causing greater than minimal interference with usual social & functional activities	Difficulty sleeping causing inability to perform usual social & functional activities	Disabling insomnia causing inability to perform basic self-care functions
Neuromuscular weakness (including myopathy & neuropathy)	Asymptomatic with decreased strength on exam OR Minimal muscle weakness causing no or minimal interference with usual social & functional activities	Muscle weakness causing greater than minimal interference with usual social & functional activities	Muscle weakness causing inability to perform usual social & functional activities	Disabling muscle weakness causing inability to perform basic self-care functions OR Respiratory muscle weakness impairing ventilation
Neurosensory alteration (including paresthesia and painful neuropathy)	Asymptomatic with sensory alteration on exam or minimal paresthesia causing no or minimal interference with usual social & functional activities	Sensory alteration or paresthesia causing greater than minimal interference with usual social & functional activities	Sensory alteration or paresthesia causing inability to perform usual social & functional activities	Disabling sensory alteration or paresthesia causing inability to perform basic self-care functions

*** Basic Self-care Functions – Adult**: Activities such as bathing, dressing, toileting, transfer/movement, continence, and feeding. **Young Children**: Activities that are age and culturally appropriate (e.g., feeding self with culturally appropriate eating implement).
**** Usual Social & Functional Activities – Adult**: Adaptive tasks and desirable activities, such as going to work, shopping, cooking, use of transportation, pursuing a hobby, etc. **Young Children**: Activities that are age and culturally appropriate (e.g., social interactions, play activities, learning tasks, etc.).

Clinical				
**Parameter**	**Grade 1 Mild**	**Grade 2 Moderate**	**Grade 3 Severe**	**Grade 4 Potentially life-threatening**
Seizure (new onset): Adult ≥18 years<br>See also Seizure (known pre-existing seizure disorder)	NA	1 seizure	2 – 4 seizures	Seizures of any kind which are prolonged, repetitive (e.g., status epilepticus), or difficult to control (e.g., refractory epilepsy)
Seizure (known preexisting seizure disorder): Adult ≥18 years<br>For worsening of existing epilepsy the grades should be based on an increase from previous level of control to any of these levels.	NA	Increased frequency of pre-existing seizures (non-repetitive) without change in seizure character OR Infrequent breakthrough seizures while on stable medication in a previously controlled seizure disorder	Change in seizure character from baseline either in duration or quality (e.g., severity or focality)	Seizures of any kind which are prolonged, repetitive (e.g., status epilepticus), or difficult to control (e.g., refractory epilepsy)
Seizure: Paediatric <18 years	Seizure, generalised onset with or without secondary generalisation, lasting <5 min with <24 hours post ictal state	Seizure, generalised onset with or without secondary generalisation, lasting 5 – 20 min with <24 hours post ictal state	Seizure, generalised onset with or without secondary generalisation, lasting >20 min	Seizure, generalised onset with or without secondary generalisation, requiring intubation and sedation
Syncope (not associated with a procedure)	NA	Present	NA	NA
Vertigo	Vertigo causing no or minimal interference with usual social & functional activities	Vertigo causing greater than minimal interference with usual social & functional activities	Vertigo causing inability to perform usual social & functional activities	Disabling vertigo causing inability to perform basic self-care functions
**Respiratory**				
Bronchospasm (acute)	Forced Expiratory Volume (FEV) 1 or peak flow reduced to 70 – 80%	FEV1 or peak flow 50 – 69%	FEV1 or peak flow 25 – 49%	Cyanosis OR FEV1 or peak flow <25% OR Intubation
Dyspnea or respiratory distress: Adult ≥14 years	Dyspnea on exertion with no or minimal interference with usual social & functional activities	Dyspnea on exertion causing greater than minimal interference with usual social & functional activities	Dyspnea at rest causing inability to perform usual social & functional activities	Respiratory failure with ventilatory support indicated

*** Basic Self-care Functions – Adult**: Activities such as bathing, dressing, toileting, transfer/movement, continence, and feeding. **Young Children**: Activities that are age and culturally appropriate (e.g., feeding self with culturally appropriate eating implement).

**** Usual Social & Functional Activities – Adult**: Adaptive tasks and desirable activities, such as going to work, shopping, cooking, use of transportation, pursuing a hobby, etc. **Young Children**: Activities that are age and culturally appropriate (e.g., social interactions, play activities, learning tasks, etc.).

Clinical				
**Parameter**	**Grade 1 Mild**	**Grade 2 Moderate**	**Grade 3 Severe**	**Grade 4 Potentially life-threatening**
Dyspnea or respiratory distress: Paediatric <14 Years	Wheezing OR Minimal increase in respiratory rate for age	Nasal flaring OR Intercostal retractions OR Pulse oximetry 90 – 95%	Dyspnea at rest causing inability to perform usual social & functional activities OR Pulse oximetry <90%	Respiratory failure with ventilatory support indicated
**Musculoskeletal**				
Arthralgia<br>See also Arthritis	Joint pain causing no or minimal interference with usual social & functional activities	Joint pain causing greater than minimal interference with usual social & functional activities	Joint pain causing inability to perform usual social & functional activities	Disabling joint pain causing inability to perform basic self-care functions
Arthritis<br>See also Arthralgia	Stiffness or joint swelling causing no or minimal interference with usual social & functional activities	Stiffness or joint swelling causing greater than minimal interference with usual social & functional activities	Stiffness or joint swelling causing inability to perform usual social & functional activities	Disabling joint stiffness or swelling causing inability to perform basic self-care functions
Bone Mineral Loss: Adult ≥21 years	Bone Mineral Density (BMD) t-score<br>-2.5 to -1.0	BMD t-score <-2.5	Pathological fracture (including loss of vertebral height)	Pathologic fracture causing life-threatening consequences
Bone Mineral Loss: Paediatric <21 Years	BMD z-score<br>-2.5 to -1.0	BMD z-score <-2.5	Pathological fracture (including loss of vertebral height)	Pathologic fracture causing life-threatening consequences
Myalgia<br>(non-injection site)	Muscle pain causing no or minimal interference with usual social & functional activities	Muscle pain causing greater than minimal interference with usual social & functional activities	Muscle pain causing inability to perform usual social & functional activities	Disabling muscle pain causing inability to perform basic self-care functions
Osteonecrosis	NA	Asymptomatic with radiographic findings AND No operative intervention indicated	Symptomatic bone pain with radiographic findings OR Operative intervention indicated	Disabling bone pain with radiographic findings causing inability to perform basic self-care functions

*** Basic Self-care Functions – Adult**: Activities such as bathing, dressing, toileting, transfer/movement, continence, and feeding. **Young Children**: Activities that are age and culturally appropriate (e.g., feeding self with culturally appropriate eating implement).
**** Usual Social & Functional Activities – Adult**: Adaptive tasks and desirable activities, such as going to work, shopping, cooking, use of transportation, pursuing a hobby, etc. **Young Children**: Activities that are age and culturally appropriate (e.g., social interactions, play activities, learning tasks, etc.).

**Clinical**				
**Parameter**	**Grade 1 Mild**	**Grade 2 Moderate**	**Grade 3 Severe**	**Grade 4 Potentially life-threatening**
**Genitourinary**				
Cervicitis (symptoms) (For use in studies evaluating topical study agents) For other cervicitis see Infection: Infection (any other than HIV infection)	Symptoms causing no or minimal interference with usual social & functional activities	Symptoms causing greater than minimal interference with usual social & functional activities	Symptoms causing inability to perform usual social & functional activities	Symptoms causing inability to perform basic self-care functions
Cervicitis (clinical exam) (For use in studies evaluating topical study agents) For other cervicitis see Infection: Infection (any other than HIV infection)	Minimal cervical abnormalities on examination (erythema, mucopurulent discharge, or friability) OR Epithelial disruption <25% of total surface	Moderate cervical abnormalities on examination (erythema, mucopurulent discharge, or friability) OR Epithelial disruption of 25 – 49% total surface	Severe cervical abnormalities on examination (erythema, mucopurulent discharge, or friability) OR Epithelial disruption 50 – 75% total surface	Epithelial disruption >75% total surface
Inter-menstrual bleeding (IMB)	Spotting observed by participant OR Minimal blood observed during clinical or colposcopic examination	IMB not greater in duration or amount than usual menstrual cycle	IMB greater in duration or amount than usual menstrual cycle	Haemorrhage with life-threatening hypotension OR Operative intervention indicated
Urinary tract obstruction (e.g., stone)	NA	Signs or symptoms of urinary tract obstruction without hydronephrosis or renal dysfunction	Signs or symptoms of urinary tract obstruction with hydronephrosis or renal dysfunction	Obstruction causing life-threatening consequences
Vulvovaginitis (symptoms) (Use in studies evaluating topical study agents) For other vulvovaginitis see Infection: Infection (any other than HIV infection)	Symptoms causing no or minimal interference with usual social & functional activities	Symptoms causing greater than minimal interference with usual social & functional activities	Symptoms causing inability to perform usual social & functional activities	Symptoms causing inability to perform basic self-care functions
Vulvovaginitis (clinical exam) (Use in studies evaluating topical study agents) For other vulvovaginitis see Infection: Infection (any other than HIV infection)	Minimal vaginal abnormalities on examination OR Epithelial disruption <25% of total surface	Moderate vaginal abnormalities on examination OR Epithelial disruption of 25 - 49% total surface	Severe vaginal abnormalities on examination OR Epithelial disruption 50 - 75% total surface	Vaginal perforation OR Epithelial disruption >75% total surface

*** Basic Self-care Functions – Adult**: Activities such as bathing, dressing, toileting, transfer/movement, continence, and feeding. **Young Children**: Activities that are age and culturally appropriate (e.g., feeding self with culturally appropriate eating implement).
**** Usual Social & Functional Activities – Adult**: Adaptive tasks and desirable activities, such as going to work, shopping, cooking, use of transportation, pursuing a hobby, etc. **Young Children**: Activities that are age and culturally appropriate (e.g., social interactions, play activities, learning tasks, etc.).

Clinical				
**Parameter**	**Grade 1 Mild**	**Grade 2 Moderate**	**Grade 3 Severe**	**Grade 4 Potentially life-threatening**
**Ocular / Visual**				
Uveitis	Asymptomatic but detectable on exam	Symptomatic anterior uveitis OR Medical intervention indicated	Posterior or pan-uveitis OR Operative intervention indicated	Disabling visual loss in affected eye(s)
Visual changes (from baseline)	Visual changes causing no or minimal interference with usual social & functional activities	Visual changes causing greater than minimal interference with usual social & functional activities	Visual changes causing inability to perform usual social & functional activities	Disabling visual loss in affected eye(s)
**Endocrine / Metabolic**				
Abnormal fat accumulation (e.g., back of neck, breasts, abdomen)	Detectable by study participant (or by caregiver for young children and disabled adults)	Detectable on physical exam by health care provider	Disfiguring OR Obvious changes on casual visual inspection	NA
Diabetes mellitus	NA	New onset without need to initiate medication OR Modification of current medications to regain glucose control	New onset with initiation of medication indicated OR Diabetes uncontrolled despite treatment modification	Life-threatening consequences (e.g., ketoacidosis, hyperosmolar nonketotic coma)
Gynecomastia	Detectable by study participant or caregiver (for young children and disabled adults)	Detectable on physical exam by health care provider	Disfiguring OR Obvious on casual visual inspection	NA
Hyperthyroidism	Asymptomatic	Symptomatic causing greater than minimal interference with usual social & functional activities OR Thyroid suppression therapy indicated	Symptoms causing inability to perform usual social & functional activities OR Uncontrolled despite treatment modification	Life-threatening consequences (e.g., thyroid storm)
Hypothyroidism	Asymptomatic	Symptomatic causing greater than minimal interference with usual social & functional activities OR Thyroid replacement therapy indicated	Symptoms causing inability to perform usual social & functional activities OR Uncontrolled despite treatment modification	Life-threatening consequences (e.g., myxedema coma)
Lipoatrophy (e.g., fat loss from the face, extremities, buttocks)	Detectable by study participant (or by caregiver for young children and disabled adults)	Detectable on physical exam by health care provider	Disfiguring OR Obvious on casual visual inspection	NA

*** Basic Self-care Functions – Adult**: Activities such as bathing, dressing, toileting, transfer/movement, continence, and feeding. **Young Children**: Activities that are age and culturally appropriate (e.g., feeding self with culturally appropriate eating implement).
**** Usual Social & Functional Activities – Adult**: Adaptive tasks and desirable activities, such as going to work, shopping, cooking, use of transportation, pursuing a hobby, etc. **Young Children**: Activities that are age and culturally appropriate (e.g., social interactions, play activities, learning tasks, etc.).

Laboratory				
**Parameter**	**Grade 1 Mild**	**Grade 2 Moderate**	**Grade 3 Severe**	**Grade 4 Potentially life-threatening**
**Haematology**				
Absolute CD4+ count: – Adult and Paediatric >13 years (HIV NEGATIVE ONLY)	300 – 400/mm^3<br>300 – 400/µL	200 – 299/mm^3<br>200 – 299/µL	100 – 199/mm^3<br>100 – 199/µL	<100/mm^3<br><100/µL
Absolute lymphocyte count: Adult and Paediatric >13 years (HIV NEGATIVE ONLY)	600 – 650/mm^3<br>0.600 x 10^9 – 0.650 x 10^9/litre (L)	500 – 599/mm^3<br>0.500 x 10^9 – 0.599 x 10^9/L	350 – 499/mm^3<br>0.350 x 10^9 – 0.499 x 0^9/L	<350/mm^3<br><0.350 x 10^9/L
Absolute neutrophil count (ANC): Adult and Paediatric >7 days	1,000 – 1,300/mm^3<br>1.000 x 10^9 – 1.300 x 10^9/L	750 – 999/mm^3<br>0.750 x 10^9 – 0.999 x 10^9/L	500 – 749/mm^3<br>0.500 x 10^9 – 0.749 x 10^9/L	<500/mm^3<br><0.500 x 10^9/L
ANC: Infant•†, 2 – ≤7 days	1,250 – 1,500/mm^3<br>1.250 x 10^9 – 1.500 x 10^9/L	1,000 – 1,249/mm^3<br>1.000 x 10^9 – 1.249 x 10^9/L	750 – 999/mm^3<br>0.750 x 10^9 – 0.999 x 10^9/L	<750/mm^3<br><0.750 x 10^9/L
ANC: Infant•†, 1 day	4,000 – 5,000/mm^3<br>4.000 x 10^9 – 5.000 x 10^9/L	3,000 – 3,999/mm^3<br>3.000 x 10^9 – 3.999 x 10^9/L	1,500 – 2,999/mm^3<br>1.500 x 10^9 – 2.999 x 10^9/L	<1,500/mm^3<br><1.500 x 10^9/L
Fibrinogen, decreased	100 – 200 mg/dL<br>1.00 – 2.00 g/L<br>OR<br>0.75 – 0.99 x LLN	75 – 99 mg/dL<br>0.75 – 0.99 g/L<br>OR<br>0.50 – 0.74 x LLN	50 – 74 mg/dL<br>0.50 – 0.74 g/L<br>OR<br>0.25 – 0.49 x LLN	<50 mg/dL<br><0.50 g/L<br>OR<br><0.25 x LLN<br>OR<br>Associated with gross bleeding
Haemoglobin: Adult and Paediatric ≥57 days (HIV POSITIVE ONLY)	8.5 – 10.0 g/dL<br>1.32 – 1.55 millimole (mmol)/L	7.5 – 8.4 g/dL<br>1.16 – 1.31 mmol/L	6.50 – 7.4 g/dL<br>1.01 – 1.15 mmol/L	<6.5 g/dL<br><1.01 mmol/L
Haemoglobin: Adult and Paediatric ≥57 days (HIV NEGATIVE ONLY)	10.0 – 10.9 g/dL<br>1.55 – 1.69 mmol/L<br>OR<br>Any decrease<br>2.5 – 3.4 g/dL<br>0.39 – 0.53 mmol/L	9.0 – 9.9 g/dL<br>1.40 – 1.54 mmol/L<br>OR<br>Any decrease<br>3.5 – 4.4 g/dL<br>0.54 – 0.68 mmol/L	7.0 – 8.9 g/dL<br>1.09 – 1.39 mmol/L<br>OR<br>Any decrease<br>≥4.5 g/dL<br>≥0.69 mmol/L	<7.0 g/dL<br><1.09 mmol/L
Haemoglobin: Infant•† 36 – 56 days (HIV POSITIVE OR NEGATIVE)	8.5 – 9.4 g/dL<br>1.32 – 1.46 mmol/L	7.0 – 8.4 g/dL<br>1.09 – 1.31 mmol/L	6.0 – 6.9 g/dL<br>0.93 – 1.08 mmol/L	<6.00 g/dL<br><0.93 mmol/L
Haemoglobin: Infant•†, 22 – 35 days (HIV POSITIVE OR NEGATIVE)	9.5 – 10.5 g/dL<br>1.47 – 1.63 mmol/L	8.0 – 9.4 g/dL<br>1.24 – 1.46 mmol/L	7.0 – 7.9 g/dL<br>1.09 – 1.23 mmol/L	<7.00 g/dL<br><1.09 mmol/L

• Values are for term infants.
† Use age and sex appropriate values (e.g., bilirubin), including preterm infants.

Laboratory				
**Parameter**	**Grade 1<br>Mild**	**Grade 2<br>Moderate**	**Grade 3<br>Severe**	**Grade 4<br>Potentially life-threatening**
Haemoglobin:<br>Infant•†, 1 – 21 days<br>(HIV POSITIVE OR NEGATIVE)	12.0 – 13.0 g/dL<br>1.86 – 2.02 mmol/L	10.0 – 11.9 g/dL<br>1.55 – 1.85 mmol/L	9.0 – 9.9 g/dL<br>1.40 – 1.54 mmol/L	<9.0 g/dL<br><1.40 mmol/L
International Normalised Ratio of prothrombin time	1.1 – 1.5 x ULN	1.6 – 2.0 x ULN	2.1 – 3.0 x ULN	>3.0 x ULN
Methemoglobin	5.0 – 10.0%	10.1 – 15.0%	15.1 – 20.0%	>20.0%
Prothrombin Time	1.1 – 1.25 x ULN	1.26 – 1.50 x ULN	1.51 – 3.00 x ULN	>3.00 x ULN
Partial Thromboplastin Time	1.1 – 1.66 x ULN	1.67 – 2.33 x ULN	2.34 – 3.00 x ULN	>3.00 x ULN
Platelets, decreased	100,000 – 124,999/mm^3<br>100.000 x 10^9 – 124.999 x 10^9/L	50,000 – 99,999/mm^3<br>50.000 x 10^9 – 99.999 x 10^9/L	25,000 – 49,999/mm^3<br>25.000 x 10^9 – 49.999 x 10^9/L	<25,000/mm^3<br><25.000 x 10^9/L
WBC, decreased	2,000 – 2,500/mm^3<br>2.000 x 10^9 – 2.500 x 10^9/L	1,500 – 1,999/mm^3<br>1.500 x 10^9 – 1.999 x 10^9/L	1,000 – 1,499/mm^3<br>1.000 x 10^9 – 1.499 x 10^9/L	<1,000/mm^3<br><1.000 x 10^9/L
**Chemistries**				
Acidosis	NA	Potential of hydrogen (pH) <normal, but ≥7.3	pH <7.3 without life-threatening Consequences	pH <7.3 with life-threatening consequences
Albumin, serum, low	3.0 g/dL – <LLN<br>30 g/L – <LLN	2.0 – 2.9 g/dL<br>20 – 29 g/L	<2.0 g/dL<br><20 g/L	NA
Alkaline Phosphatase	1.25 – 2.5 x ULN†	2.6 – 5.0 x ULN†	5.1 – 10.0 x ULN†	>10.0 x ULN†
Alkalosis	NA	pH >normal, but ≤7.5	pH >7.5 without life-threatening Consequences	pH >7.5 with life-threatening consequences
ALT (SGPT)	1.25 – 2.5 x ULN	2.6 – 5.0 x ULN	5.1 – 10.0 x ULN	>10.0 x ULN
AST (SGOT)	1.25 – 2.5 x ULN	2.6 – 5.0 x ULN	5.1 – 10.0 x ULN	>10.0 x ULN
Bicarbonate, serum, low	16.0 milliequivalents (mEq)/L – <LLN<br>16.0 mmol/L – <LLN	11.0 – 15.9 mEq/L<br>11.0 – 15.9 mmol/L	8.0 – 10.9 mEq/L<br>8.0 – 10.9 mmol/L	<8.0 mEq/L<br><8.0 mmol/L
Bilirubin (Total): Adult and Paediatric >14 days	1.1 – 1.5 x ULN	1.6 – 2.5 x ULN	2.6 – 5.0 x ULN	>5.0 x ULN
Bilirubin (Total): Infant•†, ≤ 14 days (non-haemolytic)	NA	20.0 – 25.0 mg/dL<br>342 – 428 micromole (µmol)/L	25.1 – 30.0 mg/dL<br>429 – 513 µmol/L	>30.0 mg/dL<br>>513.0 µmol/L
Bilirubin (Total): Infant•†, ≤14 days (haemolytic)	NA	NA	20.0 – 25.0 mg/dL<br>342 – 428 µmol/L	>25.0 mg/dL<br>>428 µmol/L
Calcium, serum, high (corrected for albumin): Adult and Paediatric ≥7 days	10.6 – 11.5 mg/dL<br>2.65 – 2.88 mmol/L	11.6 – 12.5 mg/dL<br>2.89 – 3.13 mmol/L	12.6 – 13.5 mg/dL<br>3.14 – 3.38 mmol/L	>13.5 mg/dL<br>>3.38 mmol/L
Calcium, serum, high (corrected for albumin): Infant•†, <7 days	11.5 – 12.4 mg/dL<br>2.88 – 3.10 mmol/L	12.5 – 12.9 mg/dL<br>3.11 – 3.23 mmol/L	13.0 – 13.5 mg/dL<br>3.245 – 3.38 mmol/L	>13.5 mg/dL<br>>3.38 mmol/L

• Values are for term infants.
† Use age and sex appropriate values (e.g., bilirubin), including preterm infants.

Laboratory				
**Parameter**	**Grade 1 Mild**	**Grade 2 Moderate**	**Grade 3 Severe**	**Grade 4 Potentially life-threatening**
Calcium, serum, low (corrected for albumin): Adult and Paediatric ≥7 days	7.8 – 8.4 mg/dL<br>1.95 – 2.10 mmol/L	7.0 – 7.7 mg/dL<br>1.75 – 1.94 mmol/L	6.1 – 6.9 mg/dL<br>1.53 – 1.74 mmol/L	<6.1 mg/dL<br><1.53 mmol/L
Calcium, serum, low (corrected for albumin): Infant•†, <7 days	6.5 – 7.5 mg/dL<br>1.63 – 1.88 mmol/L	6.0 – 6.4 mg/dL<br>1.50 – 1.62 mmol/L	5.50 – 5.90 mg/dL<br>1.38 – 1.51 mmol/L	<5.50 mg/dL<br><1.38 mmol/L
Cardiac troponin I	NA	NA	NA	Levels consistent with myocardial infarction or unstable angina as defined by the manufacturer
Cardiac troponin T	NA	NA	NA	≥ 0.20 ng/mL<br>OR<br>Levels consistent with myocardial infarction or unstable angina as defined by the manufacturer
Cholesterol (fasting): Adult ≥18 years	200 – 239 mg/dL<br>5.18 – 6.19 mmol/L	240 – 300 mg/dL<br>6.20 – 7.77 mmol/L	>300 mg/dL<br>>7.77 mmol/L	NA
Cholesterol (fasting): Paediatric <18 years	170 – 199 mg/dL<br>4.40 – 5.15 mmol/L	200 – 300 mg/dL<br>5.16 – 7.77 mmol/L	>300 mg/dL<br>>7.77 mmol/L	NA
Creatine Kinase	3.0 – 5.9 x ULN†	6.0 – 9.9 x ULN†	10.0 – 19.9 x ULN†	≥20.0 x ULN†
Creatinine	1.1 – 1.3 x ULN†	1.4 – 1.8 x ULN†	1.9 – 3.4 x ULN†	≥3.5 x ULN†
Glucose, serum, high: Non fasting	116 – 160 mg/dL<br>6.44 – 8.88 mmol/L	161 – 250 mg/dL<br>8.89 –<br>13.88 mmol/L	251 – 500 mg/dL<br>13.89 –<br>27.75 mmol/L	>500 mg/dL<br>>27.75 mmol/L
Glucose, serum, high: Fasting	110 – 125 mg/dL<br>6.11 – 6.94 mmol/L	126 – 250 mg/dL<br>6.95 –<br>13.88 mmol/L	251 – 500 mg/dL<br>13.89 –<br>27.75 mmol/L	>500 mg/dL<br>>27.75 mmol/L
Glucose, serum, low: Adult and Paediatric ≥1 month	55 – 64 mg/dL<br>3.05 – 3.55 mmol/L	40 – 54 mg/dL<br>2.22 – 3.06 mmol/L	30 – 39 mg/dL<br>1.67 – 2.23 mmol/L	<30 mg/dL<br><1.67 mmol/L
Glucose, serum, low: Infant•†<br><1 month	50 – 54 mg/dL<br>2.78 – 3.00 mmol/L	40 – 49 mg/dL<br>2.22 – 2.77 mmol/L	30 – 39 mg/dL<br>1.67 – 2.21 mmol/L	<30 mg/dL<br><1.67 mmol/L
Lactate	<2.0 x ULN without acidosis	≥2.0 x ULN without acidosis	Increased lactate with pH <7.3 without life-threatening consequences	Increased lactate with pH <7.3 with life-threatening consequences
Low-density lipoprotein (LDL) cholesterol (fasting): Adult ≥18 years	130 – 159 mg/dL<br>3.37 – 4.12 mmol/L	160 – 190 mg/dL<br>4.13 – 4.90 mmol/L	≥190 mg/dL<br>≥4.91 mmol/L	NA
LDL cholesterol (fasting): Paediatric >2 - <18 years	110 – 129 mg/dL<br>2.85 – 3.34 mmol/L	130 – 189 mg/dL<br>3.35 – 4.90 mmol/L	≥190 mg/dL<br>≥4.91 mmol/L	NA
Lipase	1.1 – 1.5 x ULN	1.6 – 3.0 x ULN	3.1 – 5.0 x ULN	>5.0 x ULN

• Values are for term infants.
† Use age and sex appropriate values (e.g., bilirubin), including preterm infants.

**Laboratory**				
**Parameter**	**Grade 1**<br>**Mild**	**Grade 2**<br>**Moderate**	**Grade 3**<br>**Severe**	**Grade 4**<br>**Potentially life-threatening**
Magnesium, serum, low	1.2 – 1.4 mEq/L<br>0.60 – 0.70 mmol/L	0.9 – 1.1 mEq/L<br>0.45 – 0.59 mmol/L	0.6 – 0.8 mEq/L<br>0.30 – 0.44 mmol/L	<0.60 mEq/L<br><0.30 mmol/L
Pancreatic amylase	1.1 – 1.5 x ULN	1.6 – 2.0 x ULN	2.1 – 5.0 x ULN	>5.0 x ULN
Phosphate, serum, low: Adult and Paediatric >14 years	2.5 mg/dL – <LLN<br>0.81 mmol/L – <LLN	2.0 – 2.4 mg/dL<br>0.65 – 0.80 mmol/L	1.0 – 1.9 mg/dL<br>0.32 – 0.64 mmol/L	<1.00 mg/dL<br><0.32 mmol/L
Phosphate, serum, low: Paediatric 1 – 14 years	3.0 – 3.5 mg/dL<br>0.97 – 1.13 mmol/L	2.5 – 2.9 mg/dL<br>0.81 – 0.96 mmol/L	1.5 – 2.4 mg/dL<br>0.48 – 0.80 mmol/L	<1.50 mg/dL<br><0.48 mmol/L
Phosphate, serum, low: Paediatric <1 year	3.5 – 4.5 mg/dL<br>1.13 – 1.45 mmol/L	2.5 – 3.4 mg/dL<br>0.81 – 1.12 mmol/L	1.5 – 2.4 mg/dL<br>0.48 – 0.80 mmol/L	<1.50 mg/dL<br><0.48 mmol/L
Potassium, serum, high	5.6 – 6.0 mEq/L<br>5.6 – 6.0 mmol/L	6.1 – 6.5 mEq/L<br>6.1 – 6.5 mmol/L	6.6 – 7.0 mEq/L<br>6.6 – 7.0 mmol/L	>7.0 mEq/L<br>>7.0 mmol/L
Potassium, serum, low	3.0 – 3.4 mEq/L<br>3.0 – 3.4 mmol/L	2.5 – 2.9 mEq/L<br>2.5 – 2.9 mmol/L	2.0 – 2.4 mEq/L<br>2.0 – 2.4 mmol/L	<2.0 mEq/L<br><2.0 mmol/L
Sodium, serum, high	146 – 150 mEq/L<br>146 – 150 mmol/L	151 – 154 mEq/L<br>151 – 154 mmol/L	155 – 159 mEq/L<br>155 – 159 mmol/L	≥ 160 mEq/L<br>≥ 160 mmol/L
Sodium, serum, low	130 – 135 mEq/L<br>130 – 135 mmol/L	125 – 129 mEq/L<br>125 – 129 mmol/L	121 – 124 mEq/L<br>121 – 124 mmol/L	≤ 120 mEq/L<br>≤ 120 mmol/L
Triglycerides (fasting)	NA	500 – 750 mg/dL<br>5.65 – 8.48 mmol/L	751 – 1,200 mg/dL<br>8.49 – 13.56 mmol/L	>1,200 mg/dL<br>>13.56 mmol/L
Uric acid	7.5 – 10.0 mg/dL<br>0.45 – 0.59 mmol/L	10.1 – 12.0 mg/dL<br>0.60 – 0.71 mmol/L	12.1 – 15.0 mg/dL<br>0.72 – 0.89 mmol/L	>15.0 mg/dL<br>>0.89 mmol/L
URINALYSIS				
Haematuria (microscopic)	6 – 10 RBC/high power field (HPF)	>10 RBC/HPF	Gross, with or without clots OR with RBC casts	Transfusion indicated
Proteinuria, random collection	1 +	2 – 3 +	4 +	NA
Proteinuria, 24 hour collection: Adult and Paediatric ≥10 years	200 – 999 mg/24 hours (h)<br>0.200 – 0.999 g/day (d)	1,000 – 1,999 mg/24 h<br>1.000 – 1.999 g/d	2,000 – 3,500 mg/24 h<br>2.000 – 3.500 g/d	>3,500 mg/24 h<br>>3.500 g/d
Proteinuria, 24 hour collection: Paediatric >3 months - <10 years	201 – 499 mg/m^2/24 h<br>0.201 – 0.499 g/d	500 – 799 mg/m^2/24 h<br>0.500 – 0.799 g/d	800 – 1,000 mg/m^2/24 h<br>0.800 – 1.000 g/d	>1,000 mg/m^2/24 h<br>>1.000 g/d

• Values are for term infants.
† Use age and sex appropriate values (e.g., bilirubin), including preterm infants.

APPENDIX B WORLD MEDICAL ASSOCIATION DECLARATION OF HELSINKI

Ethical Principles for Medical Research Involving Human Subjects

Adopted by the 18th WMA General Assembly, Helsinki, Finland, June 1964, and amended by the:
29th WMA General Assembly, Tokyo, Japan, October 1975
35th WMA General Assembly, Venice, Italy, October 1983
41st WMA General Assembly, Hong Kong, September 1989
48th WMA General Assembly, Somerset West, Republic of South Africa, October 1996
52nd WMA General Assembly, Edinburgh, Scotland, October 2000
53rd WMA General Assembly, Washington 2002 (Note of Clarification on paragraph 29 added)
55th WMA General Assembly, Tokyo 2004 (Note of Clarification on Paragraph 30 added)
59th WMA General Assembly, Seoul, October 2008

A. INTRODUCTION

1. The World Medical Association (WMA) has developed the Declaration of Helsinki as a statement of ethical principles for medical research involving human subjects, including research on identifiable human material and data.
   The Declaration is intended to be read as a whole and each of its constituent paragraphs should not be applied without consideration of all other relevant paragraphs.
2. Although the Declaration is addressed primarily to physicians, the WMA encourages other participants in medical research involving human subjects to adopt these principles.
3. It is the duty of the physician to promote and safeguard the health of patients, including those who are involved in medical research. The physician's knowledge and conscience are dedicated to the fulfilment of this duty.
4. The Declaration of Geneva of the WMA binds the physician with the words, "The health of my patient will be my first consideration," and the International Code of Medical Ethics declares that, "A physician shall act in the patient's best interest when providing medical care."
5. Medical progress is based on research that ultimately must include studies involving human subjects. Populations that are underrepresented in medical research should be provided appropriate access to participation in research.
6. In medical research involving human subjects, the well-being of the individual research subject must take precedence over all other interests.
7. The primary purpose of medical research involving human subjects is to understand the causes, development and effects of diseases and improve preventive, diagnostic and therapeutic interventions (methods, procedures and treatments). Even the best current interventions must be evaluated continually through research for their safety, effectiveness, efficiency, accessibility and quality.
8. In medical practice and in medical research, most interventions involve risks and burdens.
9. Medical research is subject to ethical standards that promote respect for all human subjects and protect their health and rights. Some research populations are particularly vulnerable and need special protection. These include those who cannot give or refuse consent for themselves and those who may be vulnerable to coercion or undue influence.
10. Physicians should consider the ethical, legal and regulatory norms and standards for research involving human subjects in their own countries as well as applicable international norms and standards. No national or international ethical, legal or

regulatory requirement should reduce or eliminate any of the protections for research subjects set forth in this Declaration.

B. BASIC PRINCIPLES FOR ALL MEDICAL RESEARCH

11. It is the duty of physicians who participate in medical research to protect the life, health, dignity, integrity, right to self-determination, privacy, and confidentiality of personal information of research subjects.
12. Medical research involving human subjects must conform to generally accepted scientific principles, be based on a thorough knowledge of the scientific literature, other relevant sources of information, and adequate laboratory and, as appropriate, animal experimentation. The welfare of animals used for research must be respected.
13. Appropriate caution must be exercised in the conduct of medical research that may harm the environment.
14. The design and performance of each research study involving human subjects must be clearly described in a research protocol. The protocol should contain a statement of the ethical considerations involved and should indicate how the principles in this Declaration have been addressed. The protocol should include information regarding funding, sponsors, institutional affiliations, other potential conflicts of interest, incentives for subjects and provisions for treating and/or compensating subjects who are harmed as a consequence of participation in the research study. The protocol should describe arrangements for post-study access by study subjects to interventions identified as beneficial in the study or access to other appropriate care or benefits.
15. The research protocol must be submitted for consideration, comment, guidance and approval to a research ethics committee before the study begins. This committee must be independent of the researcher, the sponsor and any other undue influence. It must take into consideration the laws and regulations of the country or countries in which the research is to be performed as well as applicable international norms and standards but these must not be allowed to reduce or eliminate any of the protections for research subjects set forth in this Declaration. The committee must have the right to monitor ongoing studies. The researcher must provide monitoring information to the committee, especially information about any serious adverse events. No change to the protocol may be made without consideration and approval by the committee.
16. Medical research involving human subjects must be conducted only by individuals with the appropriate scientific training and qualifications. Research on patients or healthy volunteers requires the supervision of a competent and appropriately qualified physician or other health care professional. The responsibility for the protection of research subjects must always rest with the physician or other health care professional and never the research subjects, even though they have given consent.
17. Medical research involving a disadvantaged or vulnerable population or community is only justified if the research is responsive to the health needs and priorities of this population or community and if there is a reasonable likelihood that this population or community stands to benefit from the results of the research.
18. Every medical research study involving human subjects must be preceded by careful assessment of predictable risks and burdens to the individuals and communities involved in the research in comparison with foreseeable benefits to them and to other individuals or communities affected by the condition under investigation.
19. Every clinical trial must be registered in a publicly accessible database before recruitment of the first subject.
20. Physicians may not participate in a research study involving human subjects unless they are confident that the risks involved have been adequately assessed and can be satisfactorily managed. Physicians must immediately stop a study when the risks are found to outweigh the potential benefits or when there is conclusive proof of positive and beneficial results.

21. Medical research involving human subjects may only be conducted if the importance of the objective outweighs the inherent risks and burdens to the research subjects.
22. Participation by competent individuals as subjects in medical research must be voluntary. Although it may be appropriate to consult family members or community leaders, no competent individual may be enrolled in a research study unless he or she freely agrees.
23. Every precaution must be taken to protect the privacy of research subjects and the confidentiality of their personal information and to minimize the impact of the study on their physical, mental and social integrity.
24. In medical research involving competent human subjects, each potential subject must be adequately informed of the aims, methods, sources of funding, any possible conflicts of interest, institutional affiliations of the researcher, the anticipated benefits and potential risks of the study and the discomfort it may entail, and any other relevant aspects of the study. The potential subject must be informed of the right to refuse to participate in the study or to withdraw consent to participate at any time without reprisal. Special attention should be given to the specific information needs of individual potential subjects as well as to the methods used to deliver the information. After ensuring that the potential subject has understood the information, the physician or another appropriately qualified individual must then seek the potential subject's freely-given informed consent, preferably in writing. If the consent cannot be expressed in writing, the non-written consent must be formally documented and witnessed.
25. For medical research using identifiable human material or data, physicians must normally seek consent for the collection, analysis, storage and/or reuse. There may be situations where consent would be impossible or impractical to obtain for such research or would pose a threat to the validity of the research. In such situations the research may be done only after consideration and approval of a research ethics committee.
26. When seeking informed consent for participation in a research study the physician should be particularly cautious if the potential subject is in a dependent relationship with the physician or may consent under duress. In such situations the informed consent should be sought by an appropriately qualified individual who is completely independent of this relationship.
27. For a potential research subject who is incompetent, the physician must seek informed consent from the legally authorized representative. These individuals must not be included in a research study that has no likelihood of benefit for them unless it is intended to promote the health of the population represented by the potential subject, the research cannot instead be performed with competent persons, and the research entails only minimal risk and minimal burden.
28. When a potential research subject who is deemed incompetent is able to give assent to decisions about participation in research, the physician must seek that assent in addition to the consent of the legally authorized representative. The potential subject's dissent should be respected.
29. Research involving subjects who are physically or mentally incapable of giving consent, for example, unconscious patients, may be done only if the physical or mental condition that prevents giving informed consent is a necessary characteristic of the research population. In such circumstances the physician should seek informed consent from the legally authorized representative. If no such representative is available and if the research cannot be delayed, the study may proceed without informed consent provided that the specific reasons for involving subjects with a condition that renders them unable to give informed consent have been stated in the research protocol and the study has been approved by a research ethics committee. Consent to remain in the research should be obtained as soon as possible from the subject or a legally authorized representative.

30. Authors, editors and publishers all have ethical obligations with regard to the publication of the results of research. Authors have a duty to make publicly available the results of their research on human subjects and are accountable for the completeness and accuracy of their reports. They should adhere to accepted guidelines for ethical reporting. Negative and inconclusive as well as positive results should be published or otherwise made publicly available. Sources of funding, institutional affiliations and conflicts of interest should be declared in the publication. Reports of research not in accordance with the principles of this Declaration should not be accepted for publication.

C. ADDITIONAL PRINCIPLES FOR MEDICAL RESEARCH COMBINED WITH MEDICAL CARE

31. The physician may combine medical research with medical care only to the extent that the research is justified by its potential preventive, diagnostic or therapeutic value and if the physician has good reason to believe that participation in the research study will not adversely affect the health of the patients who serve as research subjects.
32. The benefits, risks, burdens and effectiveness of a new intervention must be tested against those of the best current proven intervention, except in the following circumstances:

- The use of placebo, or no treatment, is acceptable in studies where no current proven intervention exists; or
- Where for compelling and scientifically sound methodological reasons the use of placebo is necessary to determine the efficacy or safety of an intervention and the patients who receive placebo or no treatment will not be subject to any risk of serious or irreversible harm. Extreme care must be taken to avoid abuse of this option.

33. At the conclusion of the study, patients entered into the study are entitled to be informed about the outcome of the study and to share any benefits that result from it, for example, access to interventions identified as beneficial in the study or to other appropriate care or benefits.
34. The physician must fully inform the patient which aspects of the care are related to the research. The refusal of a patient to participate in a study or the patient's decision to withdraw from the study must never interfere with the patient-physician relationship.
35. In the treatment of a patient, where proven interventions do not exist or have been ineffective, the physician, after seeking expert advice, with informed consent from the patient or a legally authorized representative, may use an unproven intervention if in the physician's judgement it offers hope of saving life, re-establishing health or alleviating suffering. Where possible, this intervention should be made the object of research, designed to evaluate its safety and efficacy. In all cases, new information should be recorded and, where appropriate, made publicly available.

APPENDIX C AMENDMENT 1

The purpose of this amendment is to include dose escalation stopping criteria and trial halting criteria into the protocol. Dose escalation will occur at the recommendation of the DSMB that will meet to review safety data of subjects prior to the next dose cohort being randomised for treatment. Each change is defined below:

Administrative amendments:

Section	Original Text	Revised to Read	Rationale for Change
Cover page	18 Sep 09	18 Sep 2009	Update format
Cover page	Amendment NA	Amendment One	Amendment of protocol
Cover page	Date: Not applicable (NA) (dd mmm yyyy)	Date: 09 Feb 2010 (dd mmm yyyy)	Amendment date
TOC page			Updated
Glossary of Abbreviations and definition of terms	Beta beta human chorionic gonadotropin	beta human chorionic gonadotropin	Deletion of duplicated word
Section 7.5.1 second paragraph	Opal-HIV-Gag(C)	Opal-HIV-Gag(c)	Correction of typographical error
Section 7.5.3.2 second paragraph	note	not	Correction of typographical error
Section 7.5.3.2 fourth paragraph	Remove a vial of Opal-HIV-Gag(C)	Remove a vial of Opal-HIV-Gag(c)	Correction of typographical error
Appendices	New text		Addition of appendix for amendment 1

Formal amendments:

Section	Original Text	Revised to Read	Rationale for Change
Section 4.1 third paragraph	New text	A minimum number of 7 subjects' completing the dose level for each dose cohort will be required for a DSMB review for dose escalation. A total of 7 subjects will ensure that at least 4 subjects have received	At the request of the Medicines and Healthcare products Regulatory agency. Included to allow a minimum of 4 subjects on active treatment to be evaluated by the DSMB for dose escalation

		administration of Opal-HIV-Gag(c).	
Section 4.2 second paragraph	The remainder of subjects will be randomised no sooner than 24 hours after randomisation of the sentinel subjects.	The remainder of subjects will be randomised no sooner than 2 days after randomisation of the sentinel subjects.	At the request of the local Research Ethics Committee to ensure that at least 2 days lapse between treatment of the sentinel cohort and the remaining subjects in the cohort
Section 7.3	A period of at least 24 hours must elapse between the start of treatment of the Sentinels and the start of treatment of the remaining subjects in the same cohort.	A period of at least 2 days must elapse between the start of treatment of the Sentinels and the start of treatment of the remaining subjects in the same cohort.	Altered in conjunction with the increase time between dosing of the sentinel subjects and remainder of the cohort
Section 10 third paragraph	The DSMB will make its recommendation to the Sponsor about dose escalation. Refer to the DSMB Charter for further details on the review process	The DSMB will make its recommendation to the Sponsor about dose escalation, halting or termination of the clinical trial. At each meeting the DSMB will review all available safety data and the DSMB will recommend to the Sponsor to:<br>• proceed with dose escalation<br>• pause enrolment pending either resolution of specific issues or amendment of the protocol as specified<br>• pause enrolment and all scheduled trial administrations pending either resolution of specific issues or amendment of the protocol as specified<br>• terminate the study<br>The DSMB, following a meeting to review the data, may recommend to the Sponsor and Principal Investigator not to permit further	At the request of the Medicines and Healthcare products Regulatory agency. Included into the clinical study protocol from the DSMB charter. The recommendations for dose escalation and halting of the clinical trial due to serious adverse events has been included into the section

		dose escalation if any of the following conditions are met:<br><br>• a subject experiences a SAE regarded as "probably" or "definitely" related to Opal-HIV-Gag(c) (i.e unblinded review of the event)<br>• any three subjects experience a similar severe adverse event (with a toxicity of at least grade 3) which is "probably" or "definitely" related to Opal-HIV-Gag(c) (i.e. unblinded review of the events)<br>• the Principal Investigator, DSMB and/or the Sponsor feel that consideration by the DSMB of a full interim safety summary is warranted following detection of a pattern of repeated or unresolved clinical AEs or laboratory abnormalities.<br><br>The clinical trial will be halted by the Principal Investigator and/or Sponsor for review and recommendation by the DSMB if:<br>• any three subjects experience a similar severe adverse event (with a toxicity of at least grade 3)	

		which, in the opinion of the Principal Investigator, cannot be attributed to causes other than the IMP<br>• any subject experiences a serious or severe adverse event, which, in the opinion of the Principal Investigator, contraindicates further dosing of additional subjects.<br><br>Refer to the DSMB Charter for further details of the review process.	

APPENDIX D AMENDMENT 2

The purpose of this amendment is to update the storage and transport conditions of the reconstituted IMP and to update the instructions relating to participant fasting for the Opal-HIV-1001. Each change from Amendment 1 is highlighted below.

Administrative amendments:

Section	Original Text	Revised to Read	Rationale for Change
Cover page			Addition of Amendment 2
Page ii	OPAL-HIV-1001, VERSION FINAL INCORPORATING AMENDMENT 1 DATED 09 FEB 2010	OPAL-HIV-1001, VERSION FINAL INCORPORATING AMENDMENT 2 DATED 18 AUG 2010	Updated with Amendment of protocol
Footer	Final Protocol Incorporating Final Amendment 1	Final Protocol Incorporating Final Amendment 2	Updated document title
TOC page			Updated
Section 6.2.2.2	perfomed	performed	Correction of typographical error
Table 3	reconsitiute	reconstitute	Correction of typographical error
Table 3 note	# The administered volume of IMP for all Opal-HIV-Gag(c) and placebo is 800 µL, which will consitute a total of 4% v/v of DMSO added to the 20 mL WBC enriched cells.	# The administered volume of IMP for all Opal-HIV-Gag(c) and placebo is 800 µL, which will constitute a total of 4% v/v of DMSO added to the 20 mL WBC enriched cells.	Correction of typographical error
Footer	09 Feb 2010	18 Aug 2010	Updated to date of Amendment
Footer			Updated page numbers
Appendices	New text		Addition of appendix for amendment 2

Formal amendments:

Section	Original Text	Revised to Read	Rationale for Change
Section 6.2.2	Subjects will be asked to fast from midnight the preceding night and be admitted to the clinic in the morning on D0. Water can be……	Subjects will be asked to fast for at least 4 hours prior to dosing (see section 6.5) and be admitted to the clinic in the morning on D0. Water can be…..	The current approved version of the protocol (Final incorporating Amendment 1, dated 09 Feb 2010) requires that subjects fast from midnight but participants can have water ad libitum. The purpose of the fasting is to determine blood parameters affected by food consumption to be assessed in a standardised manner. The Investigator reported to Medicines Development Limited that due to scheduling and delays in IMP preparation, some participants are not dosed until the afternoon, meaning their fasting period is in excess of that originally envisaged. Apart from comfort for the subjects, it is also potentially a safety concern that the fasting period is so long.<br><br>After consultation with the Medical Monitor on the timing and also in relation to the parameters of the blood safety assessments. We agree that it would be reasonable to reduce the total fasting period prior to the pre-dosing blood draw to be not less than 4 hours. Therefore we suggested allowing subjects to have a light breakfast before 8am, if participants are not having the administration until noon or early afternoon.
Section 6.2.4	Subjects will be asked to fast from midnight the preceding night and be admitted to the clinic in the morning	Subjects will be asked to fast for at least 4 hours prior to dosing (see section 6.5) and be admitted to the	As for change to Section 6.2.2

	on D28 (W4).	clinic in the morning on D28 (W4).	
Section 6.2.5	Subjects will be asked to fast from midnight the preceding night and be admitted to the clinic in the morning on D56 (W8) and D84 (W12) respectively.	Subjects will be asked to fast for at least 4 hours prior to dosing (see section 6.5) and be admitted to the clinic in the morning on D56 (W8) and D84 (W12) respectively...	As for change to Section 6.2.2
Table 2 Laboratory parameters	Haematology …..Ferritin, Vitamin B12 and serum folate will be tested at Baseline	Text removed	Text removed as the parameters tested should be listed in the in the Chemistry section of the table and not the Haematology section
Table 2 Laboratory parameters		Chemistry:…..Ferritin, Vitamin B12 and serum folate will be tested at Screening	New text added to the Chemistry parameters listed. The parametes were incorrectly listed as completed at Baseline. The parameters are tested at the Screening visit assessment.
Section 6	New sub-section	6.5 Fasting<br>Subjects will be asked to fast on D0, W 4, W8 and W12 visits. This will usually be from midnight the preceding evening. However, if dosing Is not scheduled to take place before midday on D0, W4, W8 or W12, a light breakfast is allowed. Subjects must fast at least 4 hours before the pre-dosing blood draw.	New section to clarify the fasting requirements of the protocol
Section 7.5.3.2 Administration of investigational medicinal product.<br>Pharmacy procedure:	The IMP in the dosing syringe is to be kept cold (refrigerated or chilled within a cool box) prior to addition to the WBC component.	The IMP in the dosing syringe is to be kept at room temperature (within an insulated “cool box”) prior to addition to the WBC component.	The text is updated to reflect changes to the storage of reconstituted IMP prior to administration. It was noted that the reconstituted IMP was crystallising in the syringe upon placement on a cold surface, or in the refrigerator or when the Pharmacy services preparation room. The diluent/placebo Dimethyl Sulfoxide (DMSO) being used for IMP

			reconstitution has a freezing point of approx 18.5°C and the recommended storage conditions for DMSO are 20 to 30°C. It was concluded that the DMSO was crystallising during the preparation and subsequent storage at approximately 4°C (in a fridge).<br><br>In response to this occurrence, and to ensure that the freezing/ crystallisation in the syringe does not occur, the pharmacy preparation area will be maintained at a higher temperature and the storage of the reconstituted IMP will be altered to be stored at room temperature (20-25°C) prior to administration. Transport and storage of the material will be continued in the "cool-box" to avoid temperature extremes however, the frozen cooling brick will not be used during the storage or transport. This change was implemented on the 5th July 2010 and there have been no further issues.
Section 7.5.3.2 Administration of investigational medicinal product.<br>Pharmacy procedure:<br>Preparation of placebo dose:<br>Number 2.	Place the syringe in a cool box for transfer to the clinical site	Place the syringe in an insulated "cool box " at room temperature for transfer to the clinical site	Updated text as a result change of storage of reconstituted IMP (see above)
Section 7.5.3.2 Administration of investigational medicinal product.<br>Pharmacy procedure:<br>Preparation of placebo dose:<br><u>Preparation of Opal-HIV-Gag(c)</u>	Place the syringe with capped needle in a cool box (at approximately 4°C) for transfer to the clinical site	Place the syringe with capped needle in a insulated "cool box" at room temperature for transfer to the clinical site	Updated text as a result change of storage of reconstituted IMP (see above)

<u>dose:</u><br>Number C.			
Section 7.5.3.2 Administration of investigational medicinal product.<br>Clinic Procedure<br>IMP storage at the clinic:	The IMP should be retained chilled at approximately 4°C upon arrival at the clinic until required	The IMP should be retained at room temperature in the insulated "cool box" upon arrival at the clinic until required	Updated text as a result change of storage of reconstituted IMP (see above)
Section 7.5.3.2 Administration of investigational medicinal product.<br><br>Clinic Procedure:<br><u>Incubation of IMP and re-infusion:</u><br>Number 8.	Remove the syringe and needle containing the IMP from the chilled environment	Remove the syringe and needle containing the IMP from the insulated cool box	Updated text as a result change of storage of reconstituted IMP (see above)
Table 5	Addition of new text	# The Investigator may continue dosing at their discretion for transient and self limiting events that may be attributed to IMP but are expected for a vaccine (such as fever) : these grade 3 event(s) must be followed to resolution (≤grade 2) before the subject is discharged from the clinic.	Text added for the clarification of transient and self limiting clinical events expected for a vaccine.
10 Data Safety Monitoring Board Review<br>5th Paragraph	Addition of note to existing text:<br>any three subjects experience a similar severe adverse event (with a toxicity of at least grade 3) which, which in the opinion of the Principal Investigator, cannot be attributed to causes other than the IMP	any three subjects experience a similar severe adverse event (with a toxicity of at least grade 3) which, which in the opinion of the Principal Investigator, cannot be attributed to causes other than the IMP (Note: transient and self limiting events 'expected' in response to receipt of vaccines (such as fever) may not result in halting.	Text added for the clarification of transient and self limiting clinical events expected for a vaccine.
Section 12.4.2	<u>Intention-to-treat Population</u>: This	<u>The Intent to Treat population</u> will	The Intent to Treat population was re-

	population will include all subjects who are randomised into the study and receive at least one dose of study medication. The exclusion of subjects who do not receive study product is justified by the fact that the decision to not initiate study medication is made with no knowledge of the randomised treatment. Subjects with major eligibility violations that are identifiable based on pre-randomisation characteristics will be excluded. Subjects who receive study medication other than that intended will be analysed according to the group to which they were randomised.	comprise all randomised subjects.	defined in the statistical analysis plan to be a more inclusive definition of the study population. It is more conservative than the previous definition and considered more appropriate for the analysis to be conducted on the study population.

APPENDIX E AMENDMENT 3

The purpose of this amendment is for administrative changes only.

Administrative amendments:

Section	Original Text	Revised to Read	Rationale for Change
Cover page			Addition of Amendment 3
	Medicines Development Limited<br>Level 10, 459 Collins Street<br>Melbourne, Victoria, Australia	Medicines Development Limited<br>Level 1, 18 Kavanagh St<br>Southbank, Victoria, 3006, Australia	Updated to reflect address change
Page ii	OPAL-HIV-1001, VERSION FINAL INCORPORATING AMENDMENT 2 DATED 18 AUG 2010	OPAL-HIV-1001, VERSION FINAL INCORPORATING AMENDMENT 3, DATED 28 JUN 2011	Updated with amendment of protocol
TOC page			Updated
Page ii	MEDICINES DEVELOPMENT LIMITED<br>Level 10 (South) 459 Collins St.<br>Melbourne, Victoria, Australia	MEDICINES DEVELOPMENT LIMITED	Removed Sponsor address on study acknowledgment page
Footer	Final Protocol Incorporating Final Amendment 2	Final Protocol Incorporating Final Amendment 3	Updated document title
	18 Aug 2010	28 Jun 2011	Updated to date of amendment
			Updated page numbers
Appendices	New text		Addition of Appendix for Amendment 3
